# Association of Non-alcoholic Fatty Liver Disease with Chronic Kidney Disease: A Systematic Review and Meta-analysis

**DOI:** 10.1371/journal.pmed.1001680

**Published:** 2014-07-22

**Authors:** Giovanni Musso, Roberto Gambino, James H. Tabibian, Mattias Ekstedt, Stergios Kechagias, Masahide Hamaguchi, Rolf Hultcrantz, Hannes Hagström, Seung Kew Yoon, Phunchai Charatcharoenwitthaya, Jacob George, Francisco Barrera, Svanhildur Hafliðadóttir, Einar Stefan Björnsson, Matthew J. Armstrong, Laurence J. Hopkins, Xin Gao, Sven Francque, An Verrijken, Yusuf Yilmaz, Keith D. Lindor, Michael Charlton, Robin Haring, Markus M. Lerch, Rainer Rettig, Henry Völzke, Seungho Ryu, Guolin Li, Linda L. Wong, Mariana Machado, Helena Cortez-Pinto, Kohichiroh Yasui, Maurizio Cassader

**Affiliations:** 1 Gradenigo Hospital, University of Turin, Turin, Italy; 2 Dept. of Medical Sciences, San Giovanni Battista Hospital, University of Turin, Turin, Italy; 3 Division of Gastroenterology and Hepatology Mayo Clinic, Rochester, Minnesota, United States of America; 4 Division of Gastroenterology and Hepatology, Linköping University, Linköping, Sweden; 5 Division of Cardiovascular Medicine, Department of Medical and Health Sciences, Linköping University, Linköping, Sweden; 6 Department of Experimental Immunology, World Premier International Immunology Frontier Research Center, Osaka University, Osaka, Japan; 7 Departments of Gastroenterology and Hepatology, Karolinska University Hospital, Karolinska Institutet, Stockholm, Sweden; 8 Division of Hepato-Gastroenterology, Department of Internal Medicine, Kangnam St. Mary Hospital, Catholic University Medical College, Seoul, Korea; 9 Division of Gastroenterology, Medicine Department, Siriraj Hospital, Mahidol University, Bangkoknoi, Bangkok, Thailand; 10 Storr Liver Unit, Westmead Millennium Institute, University of Sydney and Department of Gastroenterology and Hepatology, Westmead Hospital, Westmead, New South Wales, Australia; 11 Dept of Gastroenterology and Hepatology, Landspitali University Hospital, Hringbrau, Reykjavik, Iceland; 12 Centre for Liver Research and NIHR Biomedical Research Unit in Liver Disease, Institute of Biomedical Research, University of Birmingham, Birmingham, United Kingdom; 13 Department of Endocrinology and Metabolism, Zhongshan Hospital, Fudan University, Shanghai, China; 14 Department of Gastroenterology and Hepatology, Antwerp University Hospital, University of Antwerp, Antwerp, Belgium; 15 Department of Endocrinology, Diabetology and Metabolism, Antwerp University Hospital, University of Antwerp, Antwerp, Belgium; 16 Department of Gastroenterology, Marmara University, School of Medicine, Istanbul, Turkey; 17 Institute of Clinical Chemistry and Laboratory Medicine, Ernst-Moritz-Arndt University Greifswald, Greifswald, Germany; 18 Department of Medicine A, University Medicine Greifswald, Greifswald, Germany; 19 Institute of Physiology, Ernst-Moritz-Arndt-University Medicine Greifswald, Karlsburg, Germany; 20 Institute for Community Medicine, Ernst-Moritz-Arndt University Medicine Greifswald, Greifswald, Germany; 21 Department of Occupational and Environmental Medicine, Kangbuk Samsung Hospital, Sungkyunkwan University, School of Medicine, Seoul, Republic of Korea; 22 College of Life Sciences, Hunan Normal University, Changsha, China; 23 John A. Burns School of Medicine at University of Hawaii, Transplant Institute, Hawaii Medical Center, Honolulu, Hawaii, United States of America; 24 Department of Gastroenterology, University Hospital of Santa Maria, Institute of Molecular Medicine, Lisbon, Portugal; 25 Department of Molecular Gastroenterology and Hepatology, Kyoto Prefectural University of Medicine, Japan; The George Institute for Global Health, Australia

## Abstract

**Background:**

Chronic kidney disease (CKD) is a frequent, under-recognized condition and a risk factor for renal failure and cardiovascular disease. Increasing evidence connects non-alcoholic fatty liver disease (NAFLD) to CKD. We conducted a meta-analysis to determine whether the presence and severity of NAFLD are associated with the presence and severity of CKD.

**Methods and Findings:**

English and non-English articles from international online databases from 1980 through January 31, 2014 were searched. Observational studies assessing NAFLD by histology, imaging, or biochemistry and defining CKD as either estimated glomerular filtration rate (eGFR) <60 ml/min/1.73 m^2^ or proteinuria were included. Two reviewers extracted studies independently and in duplicate. Individual participant data (IPD) were solicited from all selected studies. Studies providing IPD were combined with studies providing only aggregate data with the two-stage method. Main outcomes were pooled using random-effects models. Sensitivity and subgroup analyses were used to explore sources of heterogeneity and the effect of potential confounders. The influences of age, whole-body/abdominal obesity, homeostasis model of insulin resistance (HOMA-IR), and duration of follow-up on effect estimates were assessed by meta-regression. Thirty-three studies (63,902 participants, 16 population-based and 17 hospital-based, 20 cross-sectional, and 13 longitudinal) were included. For 20 studies (61% of included studies, 11 cross-sectional and nine longitudinal, 29,282 participants), we obtained IPD. NAFLD was associated with an increased risk of prevalent (odds ratio [OR] 2.12, 95% CI 1.69–2.66) and incident (hazard ratio [HR] 1.79, 95% CI 1.65–1.95) CKD. Non-alcoholic steatohepatitis (NASH) was associated with a higher prevalence (OR 2.53, 95% CI 1.58–4.05) and incidence (HR 2.12, 95% CI 1.42–3.17) of CKD than simple steatosis. Advanced fibrosis was associated with a higher prevalence (OR 5.20, 95% CI 3.14–8.61) and incidence (HR 3.29, 95% CI 2.30–4.71) of CKD than non-advanced fibrosis. In all analyses, the magnitude and direction of effects remained unaffected by diabetes status, after adjustment for other risk factors, and in other subgroup and meta-regression analyses. In cross-sectional and longitudinal studies, the severity of NAFLD was positively associated with CKD stages. Limitations of analysis are the relatively small size of studies utilizing liver histology and the suboptimal sensitivity of ultrasound and biochemistry for NAFLD detection in population-based studies.

**Conclusion:**

The presence and severity of NAFLD are associated with an increased risk and severity of CKD.

*Please see later in the article for the Editors' Summary*

## Abstract 2

In a systematic review and meta-analysis, Giovanni Musso and colleagues examine the association between non-alcoholic fatty liver disease and chronic kidney disease.

*Please see later in the article for the Editors' Summary*

## Editors' Summary

**Background:**

Chronic kidney disease (CKD)—the gradual loss of kidney function—is becoming increasingly common. In the US, for example, more than 10% of the adult population (about 26 million people) and more than 25% of individuals older than 65 years have CKD. Throughout life, the kidneys perform the essential task of filtering waste products (from the normal breakdown of tissues and from food) and excess water from the blood to make urine. CKD gradually destroys the kidneys' filtration units, the rate of blood filtration decreases, and dangerous amounts of waste products build up in the blood. Symptoms of CKD, which rarely occur until the disease is very advanced, include tiredness, swollen feet, and frequent urination, particularly at night. There is no cure for CKD, but progression of the disease can be slowed by controlling high blood pressure and diabetes (two risk factors for CKD), and by adopting a healthy lifestyle. The same interventions also reduce the chances of CKD developing in the first place.

**Why Was This Study Done?:**

CKD is associated with an increased risk of end-stage renal (kidney) disease and of cardiovascular disease. These life-threatening complications are potentially preventable through early identification and treatment of CKD. Because early recognition of CKD has the potential to reduce its health-related burden, the search is on for new modifiable risk factors for CKD. One possible new risk factor is non-alcoholic fatty liver disease (NAFLD), which, like CKD is becoming increasingly common. Healthy livers contain little or no fat but, in the US, 30% of the general adult population and up to 70% of patients who are obese or have diabetes have some degree of NAFLD, which ranges in severity from simple fatty liver (steatosis), through non-alcoholic steatohepatitis (NASH), to NASH with fibrosis (scarring of the liver) and finally cirrhosis (extensive scarring). In this systematic review and meta-analysis, the researchers investigate whether NAFLD is a risk factor for CKD by looking for an association between the two conditions. A systematic review identifies all the research on a given topic using predefined criteria, meta-analysis uses statistical methods to combine the results of several studies.

**What Did the Researchers Do and Find?:**

The researchers identified 33 studies that assessed NAFLD and CKD in nearly 64,000 participants, including 20 cross-sectional studies in which participants were assessed for NAFLD and CKD at a single time point and 13 longitudinal studies in which participants were assessed for NAFLD and then followed up to see whether they subsequently developed CKD. Meta-analysis of the data from the cross-sectional studies indicated that NAFLD was associated with a 2-fold increased risk of prevalent (pre-existing) CKD (an odds ratio [OR]of 2.12; an OR indicates the chance that an outcome will occur given a particular exposure, compared to the chance of the outcome occurring in the absence of that exposure). Meta-analysis of data from the longitudinal studies indicated that NAFLD was associated with a nearly 2-fold increased risk of incident (new) CKD (a hazard ratio [HR] of 1.79; an HR indicates often a particular event happens in one group compared to how often it happens in another group, over time). NASH was associated with a higher prevalence and incidence of CKD than simple steatosis. Similarly, advanced fibrosis was associated with a higher prevalence and incidence of CKD than non-advanced fibrosis.

**What Do These Findings Mean?:**

These findings suggest that NAFLD is associated with an increased prevalence and incidence of CKD and that increased severity of liver disease is associated with an increased risk and severity of CKD. Because these associations persist after allowing for established risk factors for CKD, these findings identify NAFLD as an independent CKD risk factor. Certain aspects of the studies included in this meta-analysis (for example, only a few studies used biopsies to diagnose NAFLD; most used less sensitive tests that may have misclassified some individuals with NAFLD as normal) and the methods used in the meta-analysis may limit the accuracy of these findings. Nevertheless, these findings suggest that individuals with NAFLD should be screened for CKD even in the absence of other risk factors for the disease, and that better treatment of NAFLD may help to prevent CKD.

**Additional Information:**

Please access these websites via the online version of this summary at http://dx.doi.org/10.1371/journal.pmed.1001680.

- The US National Kidney and Urologic Diseases Information Clearinghouse provides information about all aspects of kidney disease; the US National Digestive Diseases Information Clearinghouse provides information about non-alcoholic liver disease
- The US National Kidney Disease Education Program provides resources to help improve the understanding, detection, and management of kidney disease (in English and Spanish)
- The UK National Health Service Choices website provides information for patients on chronic kidney disease, including some personal stories, and information on non-alcoholic fatty liver disease
- The US National Kidney Foundation, a not-for-profit organization, provides information about chronic kidney disease (in English and Spanish)
- The not-for-profit UK National Kidney Federation provides support and information for patients with kidney disease and for their carers
- The British Liver Trust, a not-for-profit organization, provides information about non-alcoholic fatty liver disease, including a patient story

## Introduction

Chronic kidney disease (CKD) affects 4%–13% of the Western adult population and over 25% of individuals older than 65 years [1]. CKD prevalence is continuously rising in concert with the rising epidemic of its risk factors including ageing, diabetes, obesity, metabolic syndrome, smoking, and hypertension [2]–[4]. In the United States, over 400,000 people currently receive some form of renal replacement therapy, and this number is expected to reach 2.2 million by 2030 [2]. Beside being a risk factor for end-stage renal disease (ESRD), CKD is an important cardiovascular disease (CVD) risk factor, and most patients with CKD die from CVD before any renal replacement therapy is initiated [5].

Early recognition and treatment of CKD aimed at reducing renal disease progression and CVD complications may limit its health-related burden [4]. In particular, patients with stage 3 CKD benefit the most from early referral strategies [6]. Despite these premises, CKD often goes unrecognized: in the Third National Health and Nutrition Survey (NHANES III), among all individuals with stage 3 CKD, the awareness was only 8.2% [7].

The high morbidity, mortality, and health care costs associated with CKD have led investigators to seek novel modifiable risk factors. Non-alcoholic fatty liver disease (NAFLD), the hepatic manifestation of the metabolic syndrome, affects 30% of the general adult population and up to 60%–70% of diabetic and obese patients [8]. NAFLD encompasses a histological spectrum ranging from simple steatosis to non-alcoholic steatohepatitis (NASH), the latter with or without advanced fibrosis. NAFLD confers an increased risk of cirrhosis, largely limited to NASH, and of CVD, independently of metabolic syndrome and traditional risk factors and through mechanisms which remain unclear [9]. Growing experimental and epidemiological evidence suggests that NAFLD and CKD share common pathogenic mechanisms and interactions [10]. However, evidence of a link between NAFLD and CKD is uncertain due to the small study populations and the borderline associations between NAFLD and traditional risk factors for CKD in the published literature. A meta-analysis on the association of NAFLD and CKD has not been conducted to date. We therefore analysed the evidence regarding two research questions: (1) Does NAFLD affect the risk of CKD independent of major confounders? (2) Is NAFLD severity associated with the severity of CKD?

## Methods

### Data Sources and Searches

We searched English and non-English language publications on MEDLINE, Ovid MEDLINE In-Process, EMBASE, ISI Web of Science, and Cochrane Library, and abstracts from the annual American Association for the Study of Liver Disease (AASLD), the American Gastroenterological Association (AGA), the European Association for the Study of the Liver (EASL), the Digestive Disease Week (DDW), and the American Society of Nephrology (ASN) Kidney Week meetings from 1980 through January 31, 2014. Search terms were: chronic kidney disease OR CKD OR kidney function OR kidney failure OR renal disease OR renal insufficiency OR renal failure OR glomerular filtration rate (GFR) OR estimated glomerular filtration rate (eGFR) OR creatinine OR albuminuria OR microalbuminuria OR macroalbuminuria OR proteinuria OR kidney injury AND NASH OR NAFLD OR non-alcoholic steatohepatitis OR non-alcoholic fatty liver disease OR fatty liver OR liver fat OR steatosis OR liver enzymes OR transaminase OR ALT OR AST OR GGT OR severity of liver disease OR fibrosis. A full list of the search strategies in different databases is reported in Text S2.

### Study Selection

#### Inclusion criteria

Criteria were observational studies including adult (age ≥18 y) populations of any sex or ethnicity, with a diagnosis of NAFLD and CKD. NAFLD had to be diagnosed by (1) liver histology, (2) imaging (ultrasound, computer tomography, magnetic resonance imaging, or spectroscopy), or (3) biochemistry (elevations in serum AST, ALT, or GGT). Competing causes of steatosis, including alcohol consumption and viral hepatitis infection had to be excluded according to standard guidelines [8]. The presence of CKD had to be defined by (1) persistent (>3 months) GFR<60 ml/min/1.73 m^2^, as estimated using the creatinine-based Modified Diet in Renal Disease (MDRD) or Chronic Kidney Disease Epidemiology Collaboration (CKD-EPI) equations [11],[12] or cystatin C–based equation [13], (2) by creatinine clearance <60 ml/min per 1.73 m^2^ using 24-hour urinary studies, (3) persistent (>3 months) kidney damage (regardless of GFR), as defined by proteinuria (microalbuminuria or macroalbuminuria using albumin-to-creatinine ratio, 24-h albumin excretion rate, or proteinuria on fresh morning urine dipstick), (4) other abnormalities due to tubular disorders or structural abnormalities detected by electrolyte or urinary sediment alterations, histology, imaging, or (5) history of kidney transplantation [2].

#### Exclusion criteria

Excluded from the meta-analysis were nnon-human studies, letters/case reports, studies including fewer than ten individuals, articles not reporting outcomes of interest or primary data (editorials, reviews), or using inadequate case definitions. In particular, studies were excluded that did not adequately consider competing causes of hepatic steatosis including alcohol, or viral hepatitis, or that enrolled a mixed population of cirrhotic and non-cirrhotic individuals (due to the potential confounding effects of cirrhosis *per se* on GFR).

### Outcome Measures

#### Primary outcome measures

Primary outcome measures were differences in the prevalence or incidence of CKD. We compared the risk of primary outcomes between individuals with NAFLD and without NAFLD as well as across the main histological subtypes of NAFLD, since NASH and advanced fibrosis carry a significantly worse prognosis than steatosis and milder fibrosis stages, respectively [9]. The impact of NAFLD and of NAFLD histological subtypes (NASH, advanced fibrosis) on eGFR, treated as a continuous variable, and on proteinuria, was also examined.

#### Secondary outcome measures

The severity of CKD was the secondary outcome measure. We estimated the effect of the severity of liver disease in NAFLD, as defined by NASH or advanced fibrosis, on the stage of CKD. CKD stage was categorized by GFR according to recent guidelines into CKD stage 3b (eGFR 30–44 ml/min/1.73 m^2^, CKD stage 4 (eGFR 15–29 ml/min/1.73 m^2^), and CKD stage 5 (eGFR<15 ml/min/1.73 m^2^) [2].

### Data Extraction and Quality Assessment

Data were extracted from each study independently and in duplicate by two authors (GM, RG), using a predefined protocol (supplied in Text S2) and a data extraction sheet based on the Cochrane Handbook for Systematic Reviews of Intervention [14]. The analysis was carried out in concordance with the Cochrane Handbook of Systematic Reviews and reported according to PRISMA guidelines (Text S1) [15]. The initial agreement between the two reviewers for selection and validity assessment of the studies was evaluated by the Kappa coefficient. Discrepancies between the reviewers were resolved by joint discussion and mutual agreement.

Methodological quality of studies was assessed by the 22-item STROBE score [16], with two items additionally incorporated into the checklist. For imaging assessment of NAFLD, examiners had to be blinded to clinical data, and the exam had to follow pre-specified, standardized criteria to detect steatosis [17]. For histological assessment of NAFLD, adequate biopsy specimens with a fragment length ≥1.5 cm with more than six portal tracts had to be obtained and scored by a blinded pathologist according to standard criteria [8].

### Data Synthesis and Analysis

For all included studies, individual participant data (IPD) was solicited from principal investigators (PIs). PIs were asked to provide the most complete and updated data, even if the follow-up was longer than that used for their respective publications. The quality of the submitted IPD was assessed using pre-specified methods (see protocol in Text S2), and any inconsistencies were clarified with the PIs.

Data not available upon database closure, either because the IPD had not been provided or because full manuscripts had not been published, were not included in our analyses.

For all analyses, we combined studies providing IPD and studies providing aggregate data (AD) into a pooled effect measure using the two-stage method [18],[19]: first, the available IPD were reduced to AD in each study, then these AD (from the IPD studies) were combined with the existing AD (from the AD studies) using standard meta-analysis techniques.

In reducing IPD to AD, for dichotomous outcomes we used multivariate logistic regression in cross-sectional studies to obtain log odds ratio (OR) with its standard error (SE), and Cox proportional hazard model in longitudinal studies (all providing time-to-event data) to obtain log hazard ratio (HR) and its SE separately for each study. We then combined individual ORs (for cross-sectional studies) and HRs (for longitudinal studies) and their 95% CIs from all included studies. Associations with continuous outcome variables were expressed as weighted mean differences (WMD) with 95% CI. Only the most adjusted risk estimates that were reported in the studies were included in the analysis. All measures of dispersion were converted to standard deviations (SDs).

The study-specific risk estimates were pooled using random-effects model, because this approach provides a more conservative assessment of the average effect size than fixed-effects model. Significance was set at *p* = 0.05.

The I^2^ statistic and its 95% CI [20]were calculated to assess statistical heterogeneity across studies: 0% suggests no heterogeneity, 0%–25% very low heterogeneity, 25%–50% low heterogeneity, 50%–75% moderate heterogeneity, and a value of >75% high heterogeneity [14]. In case of I^2^ values ≥50%, we explored individual study characteristics and those of subgroups of the main body of evidence.

We separately analyzed cross-sectional and longitudinal studies. Furthermore, for each outcome, the results of studies defining NAFLD by histology, imaging, or liver enzyme elevation are presented separately.

Sensitivity analysis was performed by repeating the meta-analysis after one study at a time was removed to assess whether any one study significantly affected pooled estimates. Additionally, a number of subgroup analyses were planned *a priori*. These subgroup analyses included repeated analysis after excluding studies not fulfilling each STROBE item, and separate analyses for the following items: diabetes: we examined the effect of NAFLD on CKD in non-diabetic versus diabetic individuals, to assess if the presence of diabetes affects the association of NAFLD with CKD; studies simultaneously adjusting versus studies not adjusting for all the following risk factors for CKD: age, body mass index (BMI), metabolic syndrome (overall or each of its components), hypertension, smoking status; study design: population-based versus hospital-based; ethnicity (Asian versus non-Asian population), as defined by the investigators. As highlighted by the recent report of the Third Asian Forum of Chronic Kidney Disease Initiatives, there are striking differences in risk factors for CKD between Asian ancestry and the remaining ethnicities: as an example, chronic glomerulonephritis due to IgA nephropathy is among the three leading causes of CKD in Asians, while it is far less common in the rest of the world. Different susceptibility loci involved in innate and adaptive immunity have been identified in recent genome-wide association studies (GWAS), but also environmental factors like infections and regular consumption of herbal remedies, may underlie these epidemiological differences [21]–[23]. Similarly, in Asians NAFLD is often encountered in the absence of obesity and metabolic syndrome and a different genetic background (including different Apolipoprotein C3 gene variants as an example), has been proposed to account for these ethnic differences [24].

We therefore explored whether differences in epidemiology of NAFLD and CKD between Asian and non-Asian populations affect the association of NAFLD with CKD studies including exclusively non-cirrhotic patients versus studies including exclusively cirrhotic patients; methods used to estimate GFR; outcomes related to CKD: studies assessing both eGFR and proteinuria versus studies assessing solely eGFR or proteinuria; study data availability: studies providing IPD versus studies providing exclusively AD.

When eight or more comparisons were available, the effect of continuous variables including age, whole-body and abdominal obesity (as estimated by BMI and by waist circumference, respectively) [25], insulin resistance (estimated by homeostasis model assessment of insulin resistance [HOMA-IR] index), and duration of follow-up (for longitudinal studies) on the association between NAFLD and CKD was evaluated by meta-regression analysis (random effects model, within-study variance estimated with the unrestricted maximum-likelihood method).

Small study bias was examined by constructing funnel plots and by performing the Egger's test and the trim-and-fill analysis [26].

Additionally, for the primary end-point we separately performed a one-stage meta-analysis of studies providing IPD, to examine how the association of NAFLD with CKD was altered when individual patient level covariates were accounted for [19],[20]. In this analysis, data from all studies providing IPD were pooled together into a single dataset and effect estimates were calculated using multivariate logistic regression (cross-sectional studies) or Cox proportional hazard models (longitudinal studies). In these models, studies were incorporated as cluster and treated as random-effect, while covariates were treated as fixed-effect. The covariates entered in the models were age, BMI, metabolic syndrome, diabetes, hypertension, smoking status, ethnicity (Asian versus non-Asian population), presence of cirrhosis, waist circumference, HOMA-index, duration of follow-up (for longitudinal studies. We first analyzed the influence of each single pre-specified covariate on the association of NAFLD with CKD with NAFLD and covariate as fixed-effect and the study as random-effects. In a second step, we did a complete case multivariable analysis with respect to NAFLD and all pre-specified covariates.

We used RevMan 5.2 (Nordic Cochrane Center) and SAS 9.2 (SAS Institute) for additional analyses that could not be done with RevMan. The trim-and-fill analysis was performed with Comprehensive Meta-analysis 2.0 (Biostat).

## Results

**Figure 1:**
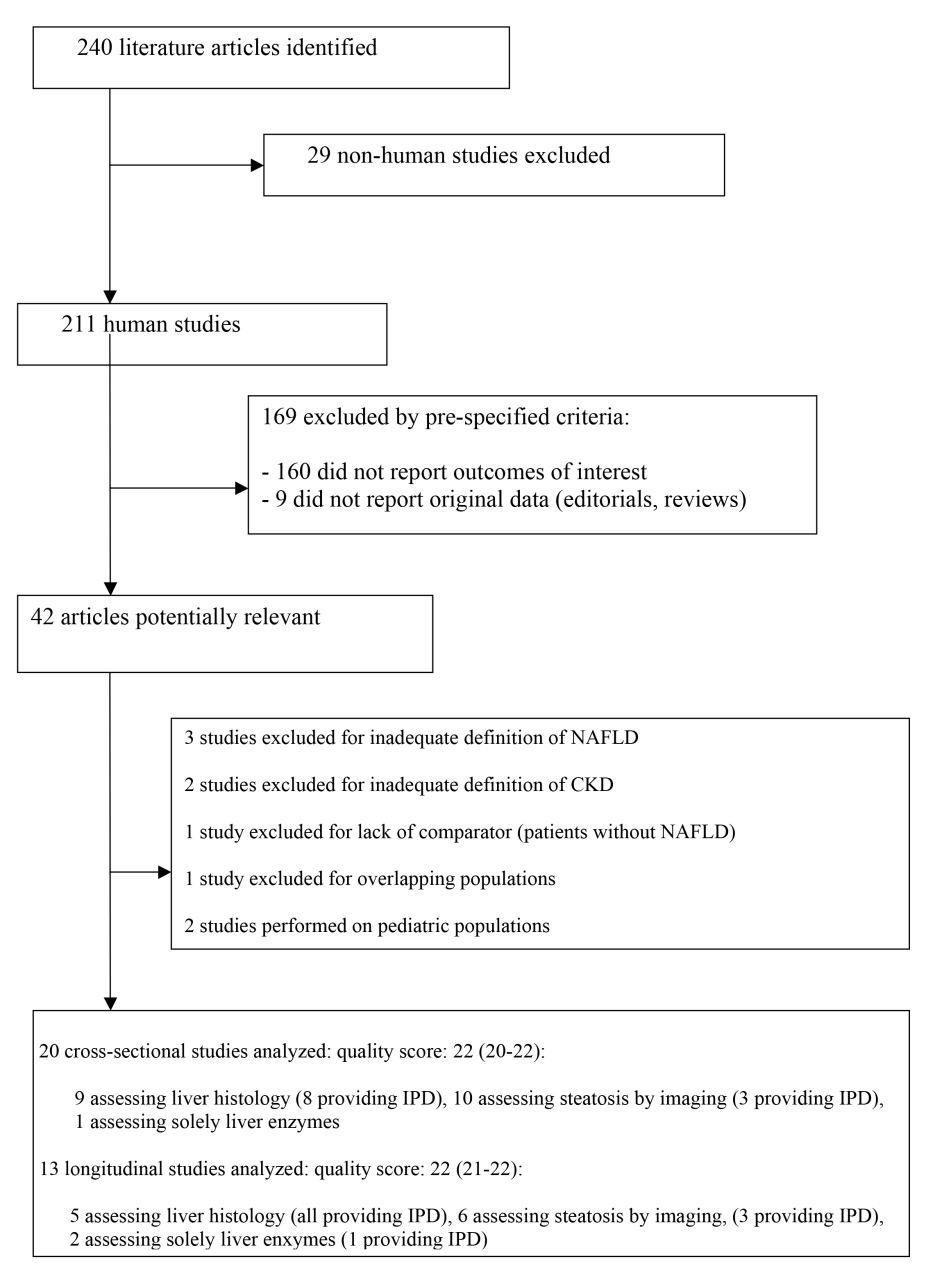
Flow of study selection. STROBE score of included studies is provided as median (range).

The mean (standard deviation [SD]) agreement between the two reviewers for study selection and for quality assessment were 0.89 (0.02) and 0.91 (0.04), respectively. The flow of study selection is reported in Figure 1.

Thirty-three studies (63,902 participants, 16 population-based and 17 hospital-based, 20 cross-sectional and 13 longitudinal) were included (Tables 1 and 2) [27]–[59]. Twenty studies (34,939 participants) were cross-sectional and evaluated the association of NAFLD with prevalent CKD [27]–[46]; 13 studies (28,963 participants) were longitudinal (mean duration of follow-up ranging three to 27 years) and evaluated the association of NAFLD with new-onset CKD [47]–[59].

**Table 1:** Cross-sectional studies connecting NAFLD to chronic kidney disease included in the meta-analysis.

Author [REF]	Study characteristics^a^	CVD risk factors	Liver disease diagnosis and prevalence	CKD diagnosis and prevalence	Adjustments	Study Data [STROBE score]^b^
Campos [27]	Hospital; n = 197; mean age 43 y; male 16%; Asian 0%	Smokers 26%; DM 26%; HTN 56%; Mean BMI 48 kg/m^2^; Met Sy 24%	Histology; NAFLD 63%, NASH 32%	eGFR<60 ml/min/1.73 m^2^ (CKD-EPI); 10%	Age, gender, BMI, waist circumference, HTN, Met sy	IPD [22]
Yilmaz [28]	Hospital; n = 87; mean age 47 y; male 55%; Asian 0%	Smokers 16%; DM 0%; HTN 30%; Mean BMI 30 kg/m^2^; Met Sy 37%	Histology; NAFLD 100%,NASH 67%	eGFR<60 ml/min/1.73 m^2^ (CKD-EPI) or AER 30–300 mg/d; 16%	Age, gender, BMI, waist circumference, BP, Tg, HDL-C, HOMA, smoking prediabetes, Met Sy	IPD [21(v)]
Targher 2010 [29]	Hospital; n = 160; mean age 51 y; male 63%; Asian 0%	Smokers 21%; DM 6%; HTN 60%; Mean BMI 27 kg/m^2^; Met Sy 29%	Histology; NASH 100%	eGFR<60 ml/min/1.73 m^2^ (MDRD) or ACR≥30 mg/g; 14%	Age, gender, BMI, waist circumference, Tg, smoking, HOMA, Met Sy diabetes, BP	AD [22]
Park 2011 [30]	Hospital; n = 562; mean age 53 y; male 68%; Asian 56%	Smokers 53%; DM 25%; HTN 30%; Mean BMI 30 kg/m^2^; Met Sy NA	All cirrhotic:12% NASH-related; 88% of other aetiologies;Matched for MELD and Child-Pugh score	eGFR<60 ml/min/1.73 m^2^ (MDRD); 17%	Obesity, DM, HTN, smoking, cardiovascular disease	IPD [21(s)]
Yasui [31]	Hospital; n = 169; mean age 54 y; male 59%; Asian 100%	Smokers 23%; DM 31%; HTN 34%; Mean BMI 26 kg/m^2^; Met Sy 30%	Histology;NAFLD 100%, NASH 53%	eGFR<60 ml/min/1.73 m^2^ (CKD-EPI) or morning dipstick proteinuria ≥1+; 14%	BMI, HTN, waist circumference, low HDL-C, high Tg, smoking, DM	IPD [22]
Musso [32]	Hospital; n = 80; mean age 48 y; male 67%; Asian 0%	Smokers 28%; DM 0%; HTN 52%; Mean BMI 25 kg/m^2^; Met Sy 31%	Histology;NAFLD 50%, NASH 25%	eGFR<60 ml/min/1.73 m^2^ (CKD-EPI) or AER≥30 mg/d; 20%	Age, gender, BMI, waist circumference, HTN, smoking, Met Sy	IPD [22]
Francque [33]	Hospital; n = 230; mean age 48 y; male 37%; Asian 0%	Smokers 25%; DM 0%; HTN 50%; Mean BMI 39 kg/m^2^; Met Sy 47%	HistologyNAFLD 100%NASH 52%	eGFR<60 ml/min/1.73 m^2^ (CKD-EPI) or overt proteinuria (>300 mg/d); 9%	Age, BMI, HTN, waist circumference, smoking, Met Sy	IPD [22]
Machado [34]	Hospital; n = 144; mean age 42 y; male 16%; Asian 0%	Smokers 28%; DM 26%; HTN 54%; Mean BMI 46 kg/m^2^; Met Sy 48%	HistologyNAFLD 100%NASH 25%	eGFR<60 ml/min/1.73 m^2^ (CKD-EPI); 6%	Age, AST, GGT, OSAS, BMI, waist circumference, HTN, smoking, Met Sy	IPD [22]
Kim [35]	Hospital; n = 96; mean age 39 y; male 71%; Asian 100%	Smokers 31%; DM 0%; HTN 54%; Mean BMI 28.5 kg/m^2^; Met Sy 56%	HistologyNAFLD 100%NASH 56%	eGFR<60 ml/min/1.73 m^2^ (modified MDRD) or morning dipstick proteinuria ≥1+; 25%	Age, BMI, HTN waist circumference, smoking, Met Sy, dyslipidaemia	IPD [22]
Targher Diabetologia [36]	Population; n = 2,103; mean age 61 y; male 62%; Asian 0%	Smokers 23%; DM 100%; HTN 66%; Mean BMI 27 kg/m^2^; Met Sy 52%	Ultrasound;67%	eGFR<60 ml/min/1.73 m^2^ (MDRD) or ACR≥30 mg/g; 13.5%	Age, gender, BMI, waist circumference, HTN, smoking, LDL.C, Tg, DM duration, HbA1c, medications, microalbuminuria, retinopathy	AD [22]
Casoinic [37]	Hospital; n = 145; mean age 61 y; male 59%; Asian 0%	Smokers 28%; DM 100%; HTN 55%; Mean BMI 28 kg/m^2^; Met Sy 80%	Ultrasound;51%	eGFR<60 ml/min/1.73 m^2^ (CKD-EPI) or ACR 30–300 mg/g; 10%	Age, gender, C-reactive protein	AD [21(p)]
Hwang [38]	Population; n = 1,361; mean age 48 y; male 71%; Asian 100%	Smokers 43%; DM 30%; HTN 15%; Mean BMI 25 kg/m^2^; Met Sy 21%	Ultrasound;43%	eGFR<60 ml/min/1.73 m^2^ (CKD-EPI) or ACR 30–300 mg/g; 16%	Age, gender, BMI, waist circumference, Tg, LDL-C, AST, ALT, GGT, HOMA,HTN, HbA1c, smoking, Met Sy	AD [22]
Targher Diab Med [39]	Hospital; n = 343; mean age 44 y; male 45%; Asian 0%	Smokers 23%; DM 100%; HTN 43%; Mean BMI 24 kg/m^2^; Met Sy 46%	Ultrasound;53%	eGFR<60 ml/min/1.73 m^2^ (MDRD) or ACR≥30 mg/g; 40%	Age, gender, BMI, physical activity, family history of CVD, sys BP, Tg, HDL-C, smoking, DM duration, HbA1c, medications, microalbuminuria, eGFR	AD [22]
Sirota [40]	Population; n = 11,469; mean age 42 y; male 45%; Asian 3.6%	Smokers 24%; DM 7%; HTN 25%; Mean BMI 25 kg/m^2^; Met Sy 28%	Ultrasound;36%	eGFR<60 ml/min/1.73 m^2^ (MDRD) or ACR≥30 mg/g; 25%	Age, gender, race, HTN, DM, sys BP, waist circumference, Tg, HDL-C, HOMA	AD [21(g)]
Li [41]	Population; n = 1,412; mean age 43 y; male 64%; Asian 100%	Smokers 42%; DM 0%; HTN 17%; Mean BMI 24 kg/m^2^; Met Sy 11%	Ultrasound;33%	eGFR<60 ml/min/1.73 m^2^ (CKD-EPI) or morning dipstick proteinuria ≥1+; 5%	Age, gender, BMI, alcohol intake, smoking, sleep quality, physical activity, BP, Tg, cholesterol, Met Sy, AST, ALT	IPD [20(s, t)]
Armstrong [42]	Population; n = 146; mean age 57 y; male 38%; Asian 5%	Smokers NA; DM 0%; HTN 36%; Mean BMI 28.8 kg/m^2^; Met Sy NA	Ultrasound;50%	eGFR<60 ml/min/1.73 m^2^ (CKD-EPI); 25%	BMI, HTN	IPD [22]
Xia [43]	Population; n = 1,141; mean age 62 y; male 43%; Asian 100%	Smokers 15%; DM 19%; HTN 38%; Mean BMI 24 kg/m^2^; Met Sy 32%	Ultrasound;41%	eGFR<60 ml/min/1.73 m^2^ (MDRD) or ACR>30 mg/g; 12%	Age, BMI, smoking, HTN, Met Sy, uric acid	IPD [22]
Ahn [44]	Populatiion; n = 1,706; mean age 58 y; male 55%; Asian 100%	Smokers 15%; DM 9%; HTN 38%; Mean BMI 24 kg/m^2^; Met Sy 26%	Ultrasound;32%	eGFR<60 ml/min/1.73 m^2^ (MDRD) or morning dipstick proteinuria ≥1+; 25%	Age, gender, BMI, smoking, waist circumference, AST, ALT, GGT, HTN, high TG, low HDL-C, DM	AD [21(v)]
Anjaneya [45]	Hospital; n = 200; mean age 50 y; male 50%; Asian 100%	Smokers 17%; DM 0%; HTN 32%; Mean BMI 23 kg/m^2^; Met Sy 22%	Ultrasound;50%	eGFR<60 ml/min/1.73 m^2^ (MDRD) or AER 30–300 mg/d; 47%	No adjustment	AD [20(p, s)]
Targher NMCD 2010 [46]	Population; n = 13,188; mean age 43 y; male 47%; Asian 4%	Smokers 24%; DM 8%; HTN 28%; Mean BMI 25 kg/m^2^; Met Sy 27%	Liver enzyme (GGT) elevation;10%	eGFR<60 ml/min/1.73 m^2^ (MDRD) or ACR≥30 mg/d; 14%	Age, gender, ethnicity, smoking, HTN, DM, lipid-lowering medications, BMI, waist circumference, fasting plasma glucose, total cholesterol, LDL-C, HDL-C, Tg, AST, ALT, alcohol intake, HOMA	AD [22]

Studies with different definitions of NAFLD (histology, imaging, liver enzyme elevation) were analyzed separately and are grouped together.

a Asian ethnicity was defined by birth within boundaries delineated West by the Red Sea, the Suez Canal, the Dardanelles strait, the Bosphorus the Caucasus and the Urals and East by the Bering Sea, the Japan and Indonesian archipelagos.

b Modified 25-item **STROBE score**, with the item(s) not satisfied by the study indicated in parentheses: (a) title and abstract informative and balanced; (b) background/rationale stated in the introduction; (c) objective(s) specified in the introduction; (d) study design correctly and presented early in the paper; (e) setting, locations, and relevant dates described; (f) eligibility criteria, methods of selection, and follow-up described; (g) diagnostic criteria, outcomes, exposures, predictors, potential confounders, and effect modifiers for all variables clearly defined. Specifically, regarding the definition of NAFLD: for radiological assessment: radiological exam performed by radiologists blinded to clinical data and following pre-specified, standardized criteria to detect steatosis; for histological assessment of NAFLD: adequate biopsy specimen (fragment length ≥1.5 cm with >6 portal tracts) and liver biopsy processed and scored by blinded pathologist according to standard criteria; (h) sources of data and details of methods of measurement given for each variable of interest; (i) any efforts to address potential sources of bias described; (j) how the study size was arrived at clearly explained; (k) how quantitative variables were handled in the analyses clearly explained; (l) all statistical methods, how missing data and loss to follow-up were addressed, any sensitivity analyses clearly described; (m) numbers of individuals at each stage of study reported; (n) characteristics of study participants, number of participants with missing data, average, and total follow-up time clearly described; (o) outcome events or summary measures over time reported; (p) unadjusted and confounder-adjusted estimates and their precision (e.g., 95% CI) reported; (q) analyses of subgroups and interactions, and sensitivity analyses reported; (r) key results with reference to study objectives summarised; (s) limitations of the study discussed; (t) cautious overall interpretation of results given; (u) generalizability (external validity) of the study results discussed; (v) source of funding and role of the funders described.

ACR, albumin-to-creatinine ratio; AER, albumin excretion rate; ALT, alanine aminotransferase; AST, aspartate aminotransferase; BP, blood pressure; CKD-EPI, Chronic Kidney Disease Epidemiology Collaboration; DM, diabetes mellitus; GGT, gamma-glutamyltransferase; HDL-C, high density lipoprotein cholesterol; HTN, hypertension; LDL-C, low density lipoprotein cholesterol; MELD, model for end-stage liver disease; Met Sy, metabolic syndrome; NA, not available; OSAS, obstructive sleep apnoea; Tg, triglycerides.

**Table 2:** Longitudinal studies connecting NAFLD to chronic kidney disease included in the meta-analysis.

Author [REF]	Study characteristics^a^	Duration of follow-up	CVD risk factors	Liver disease diagnosis and prevalence	CKD diagnosis and prevalence	Adjustments	Study Data [STROBE score]^b^
Adams [47]	Hospital; n = 251; mean age 47 y; male 54%; Asian 3%	14.2 years	Smokers 14%; DM 0%; HTN 26%; Mean BMI 33 kg/m^2^; Met Sy 36%	Ultrasound;Histology for 20% participants, NASH 56%	eGFR<60 ml/min/1.73 m^2^ (CKD-EPI) or ACR≥30 mg/d; 22%	Age, gender, BMI, HTN, smoking, Met Sy	IPD [22]
Ekstedt [48]	Hospital; n = 63; mean age 47 y; male 73%; Asian 0%	13.7 years	Smokers 17%; DM 0%; HTN 69%; Mean BMI 27 kg/m^2^; Met Sy 23%	Histology;NAFLD 100%NASH 51%	eGFR<60 ml/min/1.73 m^2^ (CKD-EPI); 19%	Age, BMI, HTN, high Tg, low HDL-C, Met Sy, use of statins, smoking	IPD [22]
Soderberg [49]	Hospital; n = 125; mean age 45 y; male 72%; Asian 0%	27.1 years	Smokers 34%; DM 24%; HTN 37%; Mean BMI 28 kg/m^2^; Met Sy 31%	HistologyNAFLD 67%NASH 33%	eGFR<60 ml/min/1.73 m^2^ (CKD-EPI); 27%	Age, BMI, HTN, smoking, DM, Met Sy	IPD [22]
Wong [50]	Hospital; n = 51; mean age 44 y; male 65%; Asian 100%	3.0 years	Smokers 14%; DM 50%; HTN 51%; Mean BMI 27 kg/m^2^; Met Sy 65	Histology;NAFLD 100%NASH 33%	eGFR<60 ml/min/1.73 m^2^ (CKD-EPI) or ACR≥30 mg/g; 8%	Age, BMI, DM, HTN, waist circumference, Met Sy, smoking	IPD [22]
Angulo [51]	Hospital; n = 191; mean age 51 y; male 35%; Asian 27%	12.4 years	Smokers 23%; DM 17%; HTN 32%; Mean BMI 28 kg/m^2^; Met Sy 25%	Histology;NAFLD 100%NASH 46%	eGFR<60 ml/min/1.73 m^2^ (CKD-EPI) or morning dipstick proteinuria ≥1+; 18%	Age, BMI, DM, HTN, smoking, dyslipidaemia, Met Sy	IPD [22]
Hamaguchi [52]	Population; n = 853; mean age 48 y; male 63%; Asian 100%	5.0 years	Smokers 44%; DM 0%; HTN 9%; Mean BMI 22 kg/m^2^; Met Sy 11%	Ultrasound;20%	eGFR<60 ml/min/1.73 m^2^ (Japanese MDRD) or morning dipstick proteinuria ≥1+; 28%	Age, BMI, smoking, Met Sy, sys BP, LDL-C	IPD [22]
Chang [53]	Population; n = 8,329; mean age 37 y; male 100%; Asian 100%	3.2 years	Smokers 43%; DM 0%; HTN 0%; Mean BMI 24 kg/m^2^; Met Sy 6%	Ultrasound;30%	eGFR<60 ml/min/1.73 m^2^ (MDRD) or morning dipstick proteinuria ≥1+; 4%	Age, eGFR, HOMA, dyslipidaemia, BMI, C-reactive protein, Met Sy, sys BP	IPD [22]
Targher JASN 2008 [54]	Population; n = 1,760; mean age 61 y; male 61%; Asian 0%	6.5 years	Smokers 22%; DM 100%; HTN 65%; Mean BMI 26 kg/m^2^; Met Sy 55%	Ultrasound;73%	eGFR<60 ml/min/1.73 m^2^ (MDRD) or ACR≥300 mg/g; 31%	Age, gender, BMI, waist circumference, BP, LDL-C, Tg, smoking, DM duration, HbA1c, medications, microalbuminuria, baseline eGFR	AD [22]
Lau [55]	Population; n = 2,858; mean age 48 y; male 46%; Asian 0%	5.3 years	Smokers 28%; DM 8.9%; HTN 47%; Mean BMI 27 kg/m^2^; Met Sy 24%	Ultrasound;30%	eGFR<60 ml/min/1.73 m^2^ (CKD-EPI) or ACR≥30 mg/g; 8%	Age, BMI, Met Sy, HTN, dyslipidaemia, smoking	IPD [22]
Athyros [56]	Population; n = 720; mean age 59 y; male 63%; Asian 0%	3.0 years	Smokers 7%; DM 19%; HTN 44%; Mean BMI 26 kg/m^2^; Met Sy 31%	Ultrasound;29%	eGFR<60 ml/min/1.73 m^2^ (MDRD); 2%	No adjustments	AD [21(p)]
El Azeem [57]	Population; n = 747; mean age 51 y; male 49%; Asian 0%	3.0 years	Smokers 22%; DM 57%; HTN 32%; Mean BMI 34 kg/m^2^; Met Sy 67%	Ultrasound;35%	eGFR<60 ml/min/1.73 m^2^ (MDRD) or ACR≥30 mg/g; 29%	Age, BMI, HTN, dyslipidaemia, smoking, Met Sy	AD [22]
Lee [58]	Population; n = 2,478; mean age 25 y; male 45%; Asian NA	10 years	Smokers 27%; DM 1%; HTN 14%; Mean BMI 30 kg/m^2^; Met Sy NA	Liver enzyme (GGT) elevation;25%	ACR>25 mg/g; 10%	Age, gender, race, BMI, smoking, physical exercise, education, HDL-C, LDL-C, Tg	AD [20(s, t)]
Ryu [59]	Population; n = 10,337; mean age 37 y; male 100%; Asian 100%	3.5 years	Smokers 47%%; DM 0%; HTN 0%; Mean BMI 24 kg/m^2^; Met Sy 7%	Liver enzyme (GGT) elevation;24%	eGFR<60 ml/min/1.73 m^2^ (MDRD) or morning dipstick proteinuria ≥1+; 3.5%	Age, baseline eGFR, BMI, sys BP, fasting plasma glucose, total cholesterol, HDL-C, Tg, uric acid, HOMA, smoking, C-reactive protein, Met Sy, incident DM, incident HTN	IPD [21(v)]

Studies with different definitions of NAFLD (histology, imaging, liver enzyme elevation) were analyzed separately and are grouped together.

a Asian ethnicity was defined by birth within boundaries delineated West by the Red Sea, the Suez Canal, the Dardanelles strait, the Bosphorus the Caucasus and the Urals and East by the Bering Sea, the Japan and Indonesian archipelagos.

b Modified 25-item **STROBE score**, with the item(s) not satisfied by the study indicated in parentheses: (a) title and abstract informative and balanced; (b) background/rationale stated in the introduction; (c) objective(s) specified in the introduction; (d) study design correctly and presented early in the paper; (e) setting, locations, and relevant dates described; (f) eligibility criteria, methods of selection, and follow-up described; (g) diagnostic criteria, outcomes, exposures, predictors, potential confounders, and effect modifiers for all variables clearly defined. Specifically, regarding the definition of NAFLD: for radiological assessment: radiological exam performed by radiologists blinded to clinical data and following pre-specified, standardized criteria to detect steatosis; for histological assessment of NAFLD: adequate biopsy specimen (fragment length ≥1.5 cm with >6 portal tracts) and liver biopsy processed and scored by blinded pathologist according to standard criteria; (h) sources of data and details of methods of measurement given for each variable of interest; (i) any efforts to address potential sources of bias described; (j) how the study size was arrived at clearly explained; (k) how quantitative variables were handled in the analyses clearly explained; (l) all statistical methods, how missing data and loss to follow-up were addressed, any sensitivity analyses clearly described; (m) numbers of individuals at each stage of study reported; (n) characteristics of study participants, number of participants with missing data, average, and total follow-up time clearly described; (o) outcome events or summary measures over time reported; (p) unadjusted and confounder-adjusted estimates and their precision (e.g., 95% CI) reported; (q) analyses of subgroups and interactions, and sensitivity analyses reported; (r) key results with reference to study objectives summarised; (s) limitations of the study discussed; (t) cautious overall interpretation of results given; (u) generalizability (external validity) of the study results discussed; (v) source of funding and role of the funders described.

ACR, albumin-to-creatinine ratio; AER, albumin excretion rate; ALT, alanine aminotransferase; AST, aspartate aminotransferase; BP, blood pressure; CKD-EPI, Chronic Kidney Disease Epidemiology Collaboration; DM, diabetes mellitus; GGT, gamma-glutamyltransferase; HDL-C, high density lipoprotein cholesterol; HTN, hypertension; LDL-C, low density lipoprotein cholesterol; MELD, model for end-stage liver disease; Met Sy, metabolic syndrome; NA, not available; OSAS, obstructive sleep apnoea; Tg, triglycerides.

We obtained IPD for 20 studies (61% of included studies, 29,282 participants), including 11 cross-sectional studies (5,145 participants) (Table 1) [27],[28],[30]–[35],[41]–[43] and nine longitudinal studies (24,137 participants) (Table 2) [47]–[53],[55],[59].

NAFLD was defined by liver histology in 13 studies (2,205 participants) [27]–[35],[48]–[51], by ultrasound in 17 studies (35,694 participants) [36]–[45],[52]–[57], and exclusively by liver enzyme elevation in three studies (26,003 participants) (Tables 1 and 2) [46],[58],[59].

Overall, the methodological quality of the studies was good: the median (range) STROBE score was 21 (20–22). Three studies did not report confounder-adjusted estimates and their precision (STROBE item [p]) [37],[45],[56], four studies did not discuss their limitations (item [s]) [30],[41],[45],[58], three studies did not disclose funding sources and role of the funders (item [v]) [28],[44],[59], two studies did not give a cautious overall interpretation of results (item [t]) [41],[58], and one study set the diagnosis of steatosis retrospectively based on archived images of gallbladder ultrasound examinations (item [g]) (Tables 1 and 2; Figure S1 within Text S3) [40].

Twelve studies enrolled exclusively non-diabetic individuals [28],[32],[33],[35],[41],[42],[45],[47],[48],[52],[52,59], four studies enrolled exclusively diabetic patients [36],[37],[39],[54], 11 studies evaluated diabetic and non-diabetic participants separately [27],[30],[31],[34],[38],[43],[46],[49],[50],[51],[55]. Overall, separate risk estimates for diabetic and non-diabetic individuals were obtained in 27 studies (82%, 47342 participants).

Twenty-eight studies (85% of all studies, 97% of participants) adjusted for potential confounders, including all of the following: age, BMI, metabolic syndrome (overall and each component), hypertension, and smoking (Tables 1, 2, and 3) [27]–[29],[31]–[36],[38]–[41],[43],[44],[46]–[55],[57]–[59].

**Table 3:** Results of subgroup analysis for the outcome: chronic kidney disease.

Outcome	Item Assessed in Analysis	Study Feature	Cross-sectional Studies	Longitudinal Studies
			OR (95% CI), I^2^ (95% CI), *p*-Value, n-Comparisons, Participants	HR (95% CI), I^2^ (95%CI), *p*-Value, n-Comparisons, Participants
**CKD in NAFLD vs. non-NAFLD**	**STROBE score**	**Item (g) fulfilled**	2.11 (1.82–2.44) I^2^ = 29% (21%–34%), *p*<0.00001, *n* = 16, 15,543 participants	1.79 (1.65–1.95), I^2^ = 0% (0%–18%), *p*<0.00001, *n* = 12, 28,680 participants
		**Item (g) not fulfilled**	1.04 (0.89–1.21), I^2^ = NA, *p* = 0.62, *n* = 1, 11,469 participants	No study
		**Item (p) fulfilled**	2.09 (1.65–2.65), I^2^ = 78% (70%–83%), *p*<0.00001, *n* = 12, 26,667 participants	1.79 (1.65–1.95), I^2^ = 0% (0%–10%), *p*<0.00001, *n* = 11, 27,960 participants
		**Item (p) not fulfilled**	2.61(1.44–4.76) I^2^ = 0% (0%–21%), *p* = 0.002, *n* = 2, 345 participants	1.94 (0.53–7.16), I^2^ = NA, *p* = 0.32, *n* = 1, 720 participants
		**Item (s) fulfilled**	2.09 (1.61–2.70), I^2^ = 79% (71%–85%), *p*<0.00001, *n* = 14, 24,838 participants	1.79(1.64–1.95), I^2^ = 0% (0%–13%), *p*<0.00001, *n* = 11, 26,202 participants
		**Item (s) not fulfilled**	2.26 (1.62–3.15), I^2^ = 0% (0%–12%), *p*<0.00001, *n* = 3, 2,174 participants	1.87 (1.31–2.67), I^2^ = NA, *p* = 0.0005, *n* = 1, 2,478 participants
		**Item (t) fulfilled**	2.14 (1.68–2.72), I^2^ = 78% (71%–84%), *p*<0.00001, *n* = 16, 25,600 participants	1.79 (1.64–1.95), I^2^ = 0% (0%–9%), *p*<0.00001, *n* = 11, 26,202 participants
		**Item (t) not fulfilled**	2.00 (1.22–3.26), I^2^ = NA, *p*<0.00001, *n* = 1, 1,412 participants	1.87 (1.31–2.67), I^2^ = NA, *p* = 0.0005, *n* = 1, 2,478 participants
		**Item (v) fulfilled**	2.21 (1.71–2.86), I^2^ = 78% (71%–84%), *p*<0.00001, *n* = 16, 25,306 participants	1.78 (1.62–1.95), I^2^ = 0% (0%–10%), *p*<0.00001, *n* = 11, 18,343 participants
		**Item (v) not fulfilled**	1.69 (1.34–2.12), I^2^ = NA, *p*<0.00001, *n* = 1, 1,706 participants	1.85 (1.50–2.28), I^2^ = NA, *p*<0.00001, *n* = 1, 10,337 participants
	**Presence of diabetes**	**Non-diabetic participants**	2.37 (1.92–2.93), I^2^ = 23% (11%–31%), *p*<0.00001, *n* = 9, 9,687 participants	1.85 (1.22–2.28), I^2^ = 0% (0%–9%), *p*<0.00001, *n* = 7, 25,166 participants
		**Diabetic participants**	1.84 (1.43–2.37), I^2^ = 24% (18%–29%), *p* = 0.0001, *n* = 8, 4,149 participants	1.67 (1.47–1.91), I^2^ = 0% (0%–11%), *p*<0.00001, *n* = 4, 2,046 participants
	**Adjustment for age ** ***and*** ** BMI ** ***and*** ** Met Sy ** ***and*** ** hypertension ** ***and*** ** smoking**	**Studies adjusting**	2.06 (1.59–2.66), I^2^ = 81% (73%–87%), *p*<0.00001, *n* = 10, 25,959 participants	1.79 (1.64–1.95), I^2^ = 0% (0–10%), *p*<0.00001, *n* = 10, 25,482 participants
		**Studies not adjusting**	2.45 (1.69–3.54), I^2^ = 0% (0%–10%), *p*<0.00001, *n* = 4, 1,053 participants	1.88 (1.33–2.65), I^2^ = 0% (0%–11%), *p* = 0.0003, *n* = 2, 3,198 participants
	**Study design**	**Population-based**	1.96 (1.49–2.59), I^2^ = 85% (77%–90%), *p*<0.00001, *n* = 8, 25,179 participants	1.78 (1.63–1.93), I^2^ = 0% (0%–11%), *p*<0.00001, *n* = 9, 28,077 participants
		**Hospital-based**	2.37 (1.80–3.13), I^2^ = 0% (0%–14%), *p*<0.00001, *n* = 9, 1,833 participants	2.15 (1.49–3.15), I^2^ = 0% (0%–9%), *p*<0.0001, *n* = 3, 607 participants
	**Ethnicity**	**Non-Asian**	1.97 (1.71–2.27), I^2^ = 0% (0%–13%), *p*<0.00001, *n* = 11, 3,418 participants	1.70 (1.49–1.96), I^2^ = 0% (0%–10%), *p*<0.00001, *n* = 7, 5,937 participants
		**Asian**	2.32 (1.74–3.09), I^2^ = 61% (53%–68%), *p*<0.00001, *n* = 7, 6,131 participants	1.84 (1.65–2.06), I^2^ = 0% (0%–9%), *p*<0.00001, *n* = 4, 20,257 participants
	**Presence of cirrhosis**	**Studies of non-cirrhotic participants**	2.12 (1.67–2.69), I^2^ = 78% (66%–86%), *p*<0.00001, *n* = 16, 26,450 participants	1.79 (1.65–1.95), I^2^ = 0% (0%–18%, *p*<0.00001, *n* = 12, 28,680 participants
		**Studies of cirrhotic participants**	2.20 (1.22–3.95), I^2^ = NA, *p* = 0.008, *n* = 1, 562 participants	No study
	**Equation used to estimate GFR**	**Studies using MDRD equation**	1.80 (1.38–2.34), I^2^ = 83% (76%–88%), *p*<0.0001, *n* = 9, 23,109 participants	1.75 (1.58–1.95), I^2^ = 0% (0%–35%), *p*<0.00001, *n* = 6, 22,737 participants
		**Studies using CKD-EPI equation**	2.82 (2.15–3.69), I^2^ = 0% (0%–11%), *p*<0.00001, *n* = 8, 3,941 participants	1.99 (1.60–2.46), I^2^ = 0% (0%–10%), *p*<0.00001, *n* = 5, 3,465 participants
	**Outcomes related to CKD**	**Studies assessing both eGFR and proteinuria**	2.08 (1.62–2.68), I^2^ = 81% (76%–85%), *p*<0.00001, *n* = 13, 26,107 participants	1.78 (1.63–1.95), I^2^ = 0% (0%–11%), *p*<0.00001, *n* = 9, 25,357 participants
		**Studies assessing only eGFR**	2.39 (1.55–3.68), I^2^ = 0% (0%–36%), *p*<0.00001, *n* = 4, 905 participants	1.95 (1.02–3.71), I^2^ = 0% (0%–11%), *p* = 0.04, *n* = 2, 845 participants
		**Studies assessing only proteinuria**	No study	1.87 (1.31–2.67), I^2^ = NA, *p* = 0.0005, *n* = 1, 1,508 participants
	**Study data**	**IPD**	1.99 (1.56–2.52), I^2^ = 0% (0%–13%), *p*<0.00001, *n* = 7, 3,538 participants	1.89 (1.70–2.11), I^2^ = 0% (0%–8%) *p*<0.00001, *n* = 8, 22,984 participants
		**AD**	2.14 (1.59–2.89) I^2^ = 76% (80%–90%), *p*<0.00001, *n* = 10, 23,474 participants	1.64 (1.43–1.88) I^2^ = 0% (0%–10%), *p*<0.00001, *n* = 4, 5,696 participants
**CKD in NASH vs. simple steatosis**	**STROBE item**	**Item (v) fulfilled**	2.85 (1.72–4.72), I^2^ = 0% (0%–10%), *p*<0.0001, *n* = 7, 800 participants	2.12 (1.42–3.17), I^2^ = 0% (0%–19%), *p* = 0.0002, *n* = 7, 429 participants
		**Item (v) not fulfilled**	1.22 (0.35–4.31), I^2^ = NA, *p* = 0.75, *n* = 1, 87 participants	No study
	**Presence of diabetes**	**Non-diabetic participants**	2.26 (1.37–3.73), I^2^ = 0% (0%–13%), *p* = 0.001, *n* = 7, 769 participants	2.03 (1.30–3.17), I^2^ = 0% (0%–12%), *p* = 0.002, *n* = 5, 334 participants
		**Diabetic participants**	3.80 (1.47–9.81), I^2^ = 0% (0%–10%), *p* = 0.003, *n* = 3, 119 participants	2.54 (1.05–6.17), I^2^ = 0% (0%–10%), *p* = 0.04, *n* = 3, 96 participants
	**Adjustment for age ** ***and*** ** BMI ** ***and*** ** Met Sy ** ***and*** ** hypertension ** ***and*** ** smoking**	**Studies adjusting**	2.53 (1.58–4.05), I^2^ = 0% (0%–14%), *p* = 0.0001, *n* = 8, 887 participants	2.12 (1.42–3.17), I^2^ = 0% (0%–19%), *p* = 0.0002, *n* = 7, 429 participants
		**Studies not adjusting**	No study	No study
	**Study design**	**Population-based**	No study	No study
		**Hospital-based**	2.53 (1.58–4.05), I^2^ = 0% (0%–14%), *p* = 0.0001, *n* = 8, 887 participants	2.12 (1.42–3.17), I^2^ = 0% (0%–19%), *p* = 0.0002, *n* = 7, 429 participants
	**Ethnicity**	**Non-Asian**	2.53 (1.35–4.73), I^2^ = 0% (0%–12%), *p* = 0.004, *n* = 6, 622 participants	1.98 (1.28–3.06), I^2^ = 0% (0%–10%), *p* = 0.002, *n* = 5, 327 participants
		**Asian**	2.64 (1.05–6.62), I^2^ = 40% (37%–46%), *p* = 0.04, *n* = 2, 263 participants	3.08 (1.09–8.72), I^2^ = 0% (0%–9%), *p* = 0.03, *n* = 2, 102 participants
	**Presence of cirrhosis**	**Studies of non- cirrhotic participants**	2.53 (1.58–4.05), I^2^ = 0% (0%–14%), *p* = 0.0001, *n* = 8, 887 participants	2.12 (1.42–3.17), I^2^ = 0% (0%–19%), *p* = 0.0002, *n* = 7, 429 participants
		**Studies of cirrhotic participants**	No study	No study
	**Equation used to estimate GFR**	**Studies using MDRD equation**	No study	No study
		**Studies using CKD-EPI equation**	2.53 (1.58–4.05), I^2^ = 0% (0%–14%), *p* = 0.0001, *n* = 8, 887 participants	2.12 (1.42–3.17), I^2^ = 0% (0%–19%), *p* = 0.0002, *n* = 7, 429 participants
	**Outcomes related to CKD**	**Both eGFR and proteinuria**	2.37 (1.40–4.01), I^2^ = 0% (0%–13%), *p* = 0.0001, *n* = 5, 622 participants	2.01 (1.16–3.48), I^2^ = 0% (0%–10%), *p* = 0.01, *n* = 4, 282 participants
		**Only eGFR**	3.25 (1.18–8.98), I^2^ = 0% (0%–19%), *p* = 0.02, *n* = 3, 265 participants	2.26 (1.26–4.05), I^2^ = 0% (0%–12%), *p* = 0.006, *n* = 3, 147 participants
		**Only proteinuria**	No study	No study
	**Study data**	**IPD**	2.53 (1.58–4.05), I^2^ = 0% (0%–14%), *p* = 0.0001, *n* = 8, 887 participants	2.12 (1.42–3.17), I^2^ = 0% (0%–19%), *p* = 0.0002, *n* = 7, 429 participants
		**AD**	No study	No study
**CKD in advanced (stage F3) vs. non-advanced (stage F0–2) fibrosis**	**STROBE item**	**Item (v) fulfilled**	4.97 (2.89–8.55), I^2^ = 0% (0%–12%), *p*<0.00001, *n* = 8, 882 participants	3.29 (2.30–4.71), I^2^ = 0% (0%–18%), *p*<0.00001, *n* = 7, 429 participants
		**Item (v) not fulfilled**	6.94 (1.73–17.76), I^2^ = NA, *p* = 0.006, *n* = 1, 87 participants	No study
	**Presence of diabetes**	**Non-diabetic participants**	5.84(3.25–10.49), I^2^ = 0% (0%–12%), *p*<0.00001, *n* = 8, 844 participants	2.82 (1.86–4.28), I^2^ = 0% (0%–11%), *p*<0.00001, *n* = 4, 307 participants
		**Diabetic participants**	5.01 (1.46–17.21), I^2^ = 0% (0%–13%), *p* = 0.01, *n* = 3, 120 participants	4.19 (2.10–8.38), I^2^ = 0% (0%–11%), *p*<0.0001, *n* = 3, 97 participants
	**Adjustment for age ** ***and*** ** BMI ** ***and*** ** Met Sy ** ***and*** ** hypertension ** ***and*** ** smoking**	**Studies adjusting**	5.20 (3.14–8.61), I^2^ = 0% (0%–17%), *p*<0.00001, *n* = 9, 969 participants	3.29 (2.30–4.71), I^2^ = 0% (0%–18%), *p*<0.00001, *n* = 7, 429 participants
		**Studies not adjusting**	No study	No study
	**Study design**	**Population-based**	No study	No study
		**Hospital-based**	5.20 (3.14–8.61), I^2^ = 0% (0%–17%), *p*<0.00001, *n* = 9, 969 participants	3.29 (2.30–4.71), I^2^ = 0% (0%–18%), *p*<0.00001, *n* = 7, 429 participants
	**Ethnicity**	**Non-Asian**	6.00 (3.15–11.43), I^2^ = 0% (0%–10%), *p*<0.00001, *n* = 7, 704 participants	2.86 (1.93–4.22), I^2^ = 0% (0%–11%), *p*<0.00001, *n* = 5, 317 participants
		**Asian**	4.15 (1.85–9.32), I^2^ = 0% (0%–9%), *p* = 0.0006, *n* = 2, 265 participants	6.01 (2.25–16.09), I^2^ = 34% (27%–39%), *p* = 0.004, *n* = 2, 102 participants
	**Presence of cirrhosis**	**Studies of non- cirrhotic participants**	5.20 (3.14–8.61), I^2^ = 0% (0%–17%), *p*<0.00001, *n* = 9, 969 participants	3.29 (2.30–4.71), I^2^ = 0% (0%–18%), *p*<0.00001, *n* = 7, 429 participants
		**Studies of cirrhotic participants**	No study	No study
	**Equation used for estimating GFR**	**MDRD-equation**	4.07 (1.52–10.09), I^2^ = 0% (0%–11%), *p* = 0.005, *n* = 2, 176 participants	No study
		**CKD-EPI equation**	5.67 (3.15–10.20), I^2^ = 0% (0%–19%), *p*<0.00001, *n* = 7, 793 participants	3.29 (2.30–4.71), I^2^ = 0% (0%–18%), *p*<0.00001, *n* = 7, 429 participants
	**Outcomes related to CKD**	**Both eGFR and p proteinuria**	5.05 (2.95–8.66), I^2^ = 0% (0%–9%), *p*<0.00001, *n* = 6, 702 participants	3.56 (2.05–6.17), I^2^ = 16% (10%–21%), *p*<0.0001, *n* = 4, 282 participants
		**Studies assessing only eGFR**	6.36 (1.50–26.91), I^2^ = 0% (0%–16%), *p* = 0.01, *n* = 3, 267 participants	3.11 (1.85–5.22), I^2^ = 0% (0%–11%), *p*<0.0001, *n* = 3, 147 participants
		**Studies assessing only proteinuria**	No study	No study
	**Study data**	**IPD**	5.28 (3.06–9.12), I^2^ = 0% (0%–10%) *p*<0.00001, *n* = 8, 889 participants	3.29 (2.30–4.71), I^2^ = 0% (0%–18%), *p*<0.00001, *n* = 7, 429 participants
		**AD**	4.71 (1.25–17.72), I^2^ = NA, *p* = 0.02, *n* = 1, 80 participants	No study

Subgroup analysis was planned a priori to assess the impact of the following items on the association between NAFLD and CKD: (1) Fulfilment of STROBE items: we planned to repeat the analysis after excluding studies not fulfilling each STROBE item (different STROBE items are described in footnote to Table 1). (2) Diabetes: studies including exclusively non-diabetic individuals versus studies including diabetic individuals. (3) Studies simultaneously adjusting versus studies not adjusting for all the following risk factors for CKD: age and BMI and metabolic syndrome (overall or each of its components) and hypertension and smoking. (4) Study design (population-based versus community-based). (5) Ethnicity (Caucasian versus Asian). (6) Studies including only non-cirrhotic patients versus studies including cirrhotic patients. (7) Studies using the CKD-EPI versus studies using the MDRD equation to estimate GFR. (8) Outcomes related to CKD: studies assessing both eGFR and proteinuria versus studies assessing either eGFR or proteinuria. (9) Type of data available: studies with IPD versus studies with AD.

Eleven studies enrolled exclusively Asian populations [31],[35],[38],[41],[43],[44],[45],[50],[52],[53],[59], 15 studies enrolled exclusively non-Asian individuals [27]–[29],[32]–[34],[36],[37],[39],[48],[49],[54]–[57], four studies evaluated separately Asian and non-Asian participants [27],[30],[42],[47],[51]. Overall, separate risk estimates for Asian and non-Asian individuals were obtained in 30 studies (91%, 36,767 participants).

All studies included non-cirrhotic participants, except one cross-sectional study comparing NASH-related cirrhosis with cirrhosis of other aetiologies, matched for Child-Pugh and Model for End-stage Liver Disease-(MELD) scores [30].

GFR was estimated with the CKD-EPI equation in 16 studies [27],[28],[31]–[34],[37],[38],[41],[42],[47]–[51],[55] and with the MDRD equation in 17 studies [29],[30],[35],[36],[39],[40],[43]–[46],[52]–[54],[56],[57],[59]. One study assessed only proteinuria and not eGFR [58], while seven studies (21%) evaluated only eGFR and not proteinuria [27],[30],[34],[42],[48],[49],[56].

### NAFLD and Prevalent/Incident CKD

**Figure 2:**
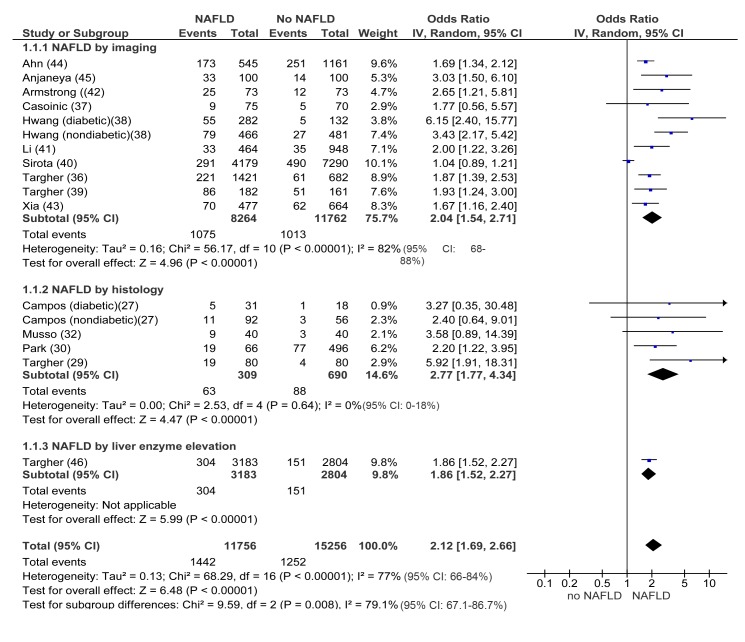
Forest plot of comparison. NAFLD versus non-NAFLD, outcome: prevalent chronic kidney disease in cross-sectional studies. Studies assessing NAFLD by imaging, histology or liver enzyme elevation were considered separately.

In cross-sectional studies, pooled OR for the presence of CKD of NAFLD versus non-NAFLD was 2.12 (95% CI 1.69–2.66, I^2^ = 77% [95% CI 66%–84%], N-comparisons = 17, *p*<0.00001) (Figure 2). The magnitude and direction of the effect were similar across different NAFLD definitions (Figure 2). Heterogeneity was high, due to the high heterogeneity in studies assessing NAFLD by ultrasound, but fell after excluding one study [40], where the diagnosis of steatosis was made retrospectively on the basis of archived videotapes of gallbladder ultrasound examinations, while pooled OR remained similar in magnitude and direction of the overall effect (OR 2.11, 95% CI 1.82–2.44; I^2^ = 29% [95% CI 15%–35%], *n* = 16, *p*<0.00001) (Table 3).

**Figure 3:**
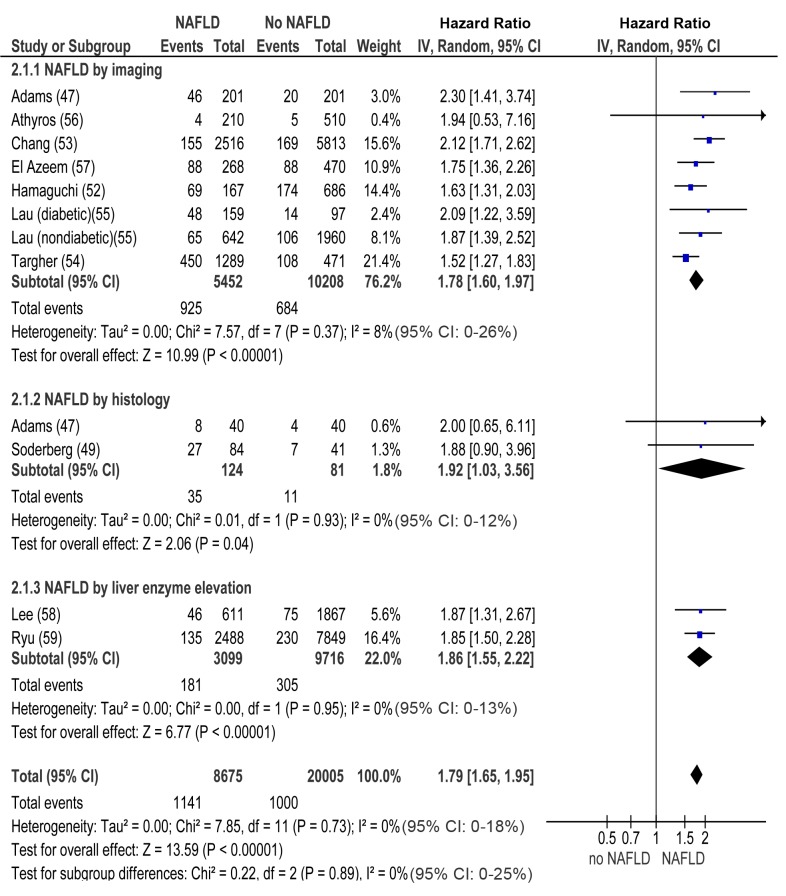
Forest plot of comparison. NAFLD versus non-NAFLD, outcome: incident chronic kidney disease in prospective studies. NAFLD was defined by imaging, histology, or liver enzyme elevation. Studies assessing NAFLD by imaging, histology, or liver enzyme elevation were considered separately.

In longitudinal studies, pooled HR for incident CKD of NAFLD versus non-NAFLD was 1.79 (95% CI 1.65–1.95, I^2^ = 0% [95% CI 0%–18%], *n* comparisons = 13, *p*<0.00001) (Figure 3). There was no heterogeneity in the meta-analysis of overall events, suggesting a consistent disease effect.

In both cross-sectional and longitudinal studies, the difference between NAFLD and non-NAFLD patients remained statistically significant even when considering eGFR as a continuous variable or when considering only proteinuria as outcome (Figures S2A, S2B, S3A, and S3B within Text S3).

In both cross-sectional and longitudinal studies, meta-regression analysis found no association between CKD and age (cross-sectional studies: β = 0.004, 95% CI −0.023 to 0.031, *p* = 0.772; longitudinal studies: β = 0.005, 95% CI −0.014 to 0.021, *p* = 0.207), BMI (cross-sectional studies: β = 0.013, 95% CI −0.034 to 0.059, *p* = 0.592; longitudinal studies: β = 0.003, 95% CI −0.019 to 0.026, *p* = 0.786), waist circumference (cross-sectional studies: β = −0.003, 95% CI −0.023 to 0.031, *p* = 0.772; longitudinal studies: β = −0.003, 95% CI −0.016 to 0.011, *p* = 0.686), HOMA-IR index (cross-sectional studies: β = 0.089, 95% CI −0.210 to 0.388, *p* = 0.559; longitudinal studies: β = −0.041, 95% CI −0.171 to 0.087, *p* = 0.524), and duration of follow-up (longitudinal studies: β = 0.002, 95% CI −0.022 to 0.026, *p* = 0.880).

The Egger's test found no strong evidence for small study bias and the trim-and-fill analysis did not appreciably attenuate the strength of the association (Figures S4A and S4B within Text S3).

### NAFLD Histological Subtypes and the Risk of CKD in Non-cirrhotic NAFLD

#### Cross-sectional studies

**Figure 4:**
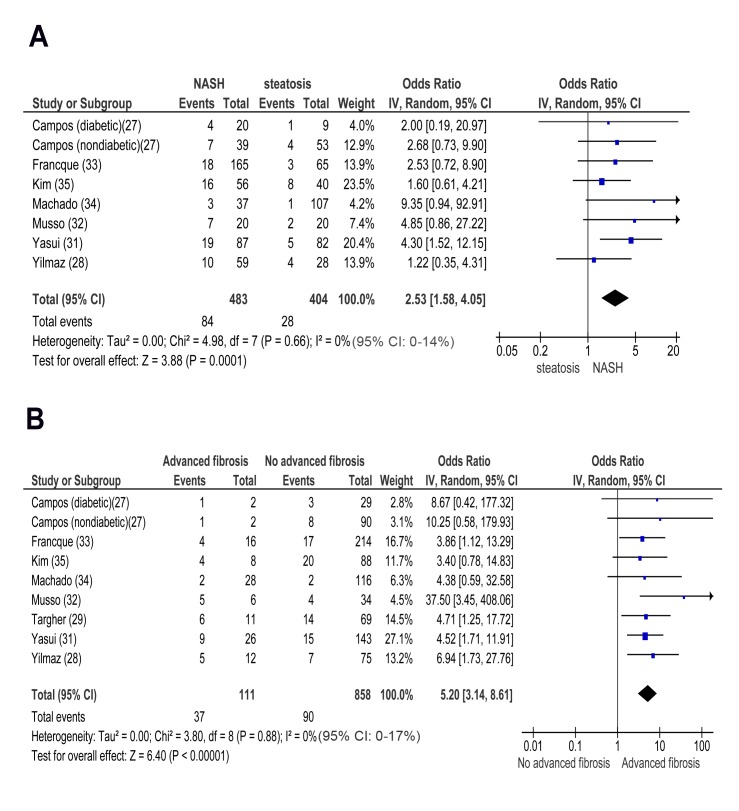
Forest plot of comparison. (A) NASH versus simple steatosis in biopsy-proven non-cirrhotic NAFLD; outcome: prevalent chronic kidney disease in cross-sectional studies. (B) Advanced (stage F3) fibrosis versus no-advanced (stage F0–F2) fibrosis in biopsy-proven non-cirrhotic NAFLD, outcome: prevalent CKD in cross-sectional studies.

In cross-sectional studies, pooled OR for CKD of NASH versus steatosis was 2.53 (95% CI 1.58–4.05, I^2^ = 0% [95% CI 0%–14%], *n*-comparisons = 8, *p* = 0.0001) (Figure 4). Pooled OR for CKD of advanced (stage F3) versus non-advanced (stage F0–F2) fibrosis was 5.20 (95% CI 3.14–8.61, I^2^ = 0% [95% CI 0%–17%], *n* = 9, *p*<0.00001) (Figure 4). There was no heterogeneity in the meta-analyses of overall events, suggesting a consistent disease effect.

NASH and advanced fibrosis were also associated with higher ORs for proteinuria and with a lower eGFR than steatosis and non-advanced fibrosis, respectively (Figures S5A, S5B, S6A, and S6B within Text S3).

Meta-regression analysis found no association between CKD and age (for NASH: β = 0.050, 95% CI −0.039 to 0.140, *p* = 0.269; for advanced fibrosis: β = 0.002, 95% CI −0.101 to 0.105, *p* = 0.964), BMI (for NASH: β = 0.003, 95% CI −0.049 to 0.056, *p* = 0.896; for advanced fibrosis: β = 0.002, 95% CI −0.007 to 0.065, *p* = 0.949), waist circumference (for NASH: β = 0.004, 95% CI −0.031 to 0.040, *p* = 0.812; for advanced fibrosis: β = −0.004, 95% CI −0.043 to 0.034, *p* = 0.820), and HOMA-IR index (for NASH: β = −0.231, 95% CI −0.691 to 0.229, *p* = 0.324; for advanced fibrosis: β = −0.161, 95% CI −0.705 to 0.383, *p* = 0.562).

The Egger's test found no strong evidence for small study bias and the trim-and-fill analysis did not appreciably attenuate the strength of the association (Figures S4A–S4D within Text S3).

#### Longitudinal studies

**Figure 5:**
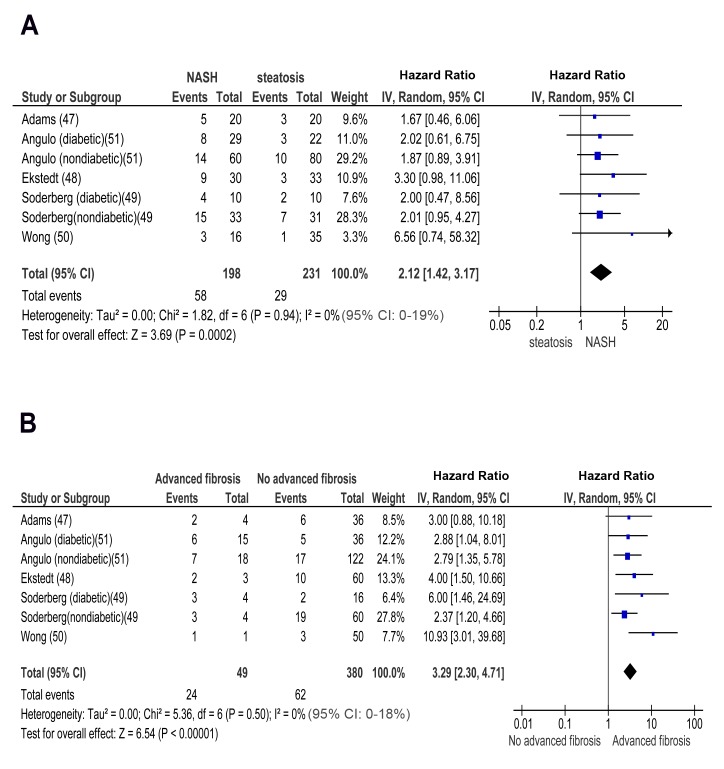
Forest plot of comparison. (A) NASH versus simple steatosis in biopsy-proven noncirrhotic NAFLD; outcome: incident CKD in prospective studies. (B) Advanced (stage F3) fibrosis versus no-advanced (stage F0–F2) fibrosis in biopsy-proven non-cirrhotic NAFLD, outcome: incident CKD in prospective studies.

In longitudinal studies, pooled HR for incident CKD of NASH versus simple steatosis was 2.12 (95% CI 1.42–3.17, I^2^ = 0% [95% CI 0%–19%, *n*-comparisons = 7, *p* = 0.0002) (Figure 5). Pooled HR for CKD of advanced fibrosis versus non-advanced fibrosis was 3.29 (95% CI 2.30–4.71, I^2^ = 0% [95% CI 0%–18%], *n* = 7, *p*<0.00001) (Figure 5). There was no heterogeneity in the meta-analyses of overall events, again suggesting a consistent disease effect.

NASH and advanced fibrosis were also associated with a higher OR for incident proteinuria and with more severe eGFR reduction than steatosis and non-advanced fibrosis, respectively (Figures S7A, S7B, S8A, and S8B within Text S3).

Meta-regression analysis found no association between CKD and age (for NASH: β = −0.019, 95% CI −0.113 to 0.774, *p* = 0.681; for advanced fibrosis: β = −0.007, 95% CI −0.088 to 0.074, *p* = 0.868), BMI (for NASH: β = −0.106, 95% CI −0.366 to 0.154, *p* = 0.425; for advanced fibrosis: β = −0.075, 95% CI −0.307 to 0.158, *p* = 0.529), waist circumference (for NASH: β = −0.026, 95% CI −0.116 to 0.060, *p* = 0.559; for advanced fibrosis: β = −0.026, 95% CI −0.101 to 0.050, *p* = 0.508), HOMA-IR index (for NASH: β = 0.167, 95% CI −0.153 to 0.487, *p* = 0.306; for advanced fibrosis: β = 0.048, 95% CI −0.376 to 0.472, *p* = 0.825) and duration of follow-up (for NASH: β = −0.012, 95% CI −0.067 to 0.043, *p* = 0.675; for advanced fibrosis: β = −0.006, 95% CI −0.058 to 0.046, *p* = 0.817).

The Egger's test found no strong evidence for small study bias and the trim-and-fill analysis did not appreciably attenuate the strength of the association (Figures S4E and S4F within Text S3).

### NAFLD Histological Subtypes and the Stage of CKD in Non-cirrhotic NAFLD

#### Cross-sectional studies

In cross-sectional studies, pooled OR for CKD stage 3b of NASH versus steatosis was 3.38 (95% CI 1.11–10.31, I^2^ = 0% [95% CI 0%–17%], *n*-comparisons = 8, *p* = 0.03) (Figure S9A within Text S3). Pooled OR for CKD stage 3b of advanced versus non-advanced fibrosis was 26.98 (95% CI 9.12–79.84, I^2^ = 0% [95% CI 0%–21%], *n* = 9 *p*<0.00001) (Figure S9B within Text S3). There was no heterogeneity in the meta-analysis of overall events, suggesting a consistent disease effect.

The presence of serum creatinine elevation, configuring severely decreased renal function (CKD stage 4) or renal failure (CKD stage 5), was an exclusion criterion in cross-sectional studies, which focused on the association of NAFLD with clinically unrecognized (stage 1–3) CKD.

#### Longitudinal studies

**Figure 6:**
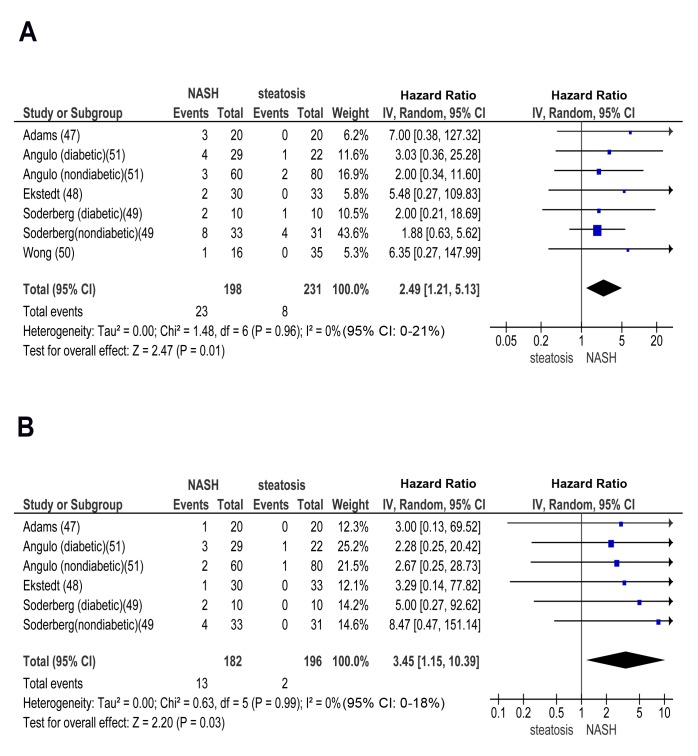
Forest plot of comparison. (A) NASH versus simple steatosis in biopsy-proven non-cirrhotic NAFLD; outcome: incident CKD stage 3b in prospective studies. (B) NASH versus simple steatosis in biopsy-proven non-cirrhotic NAFLD; outcome: incident CKD stage 4 in prospective studies.

**Figure 7:**
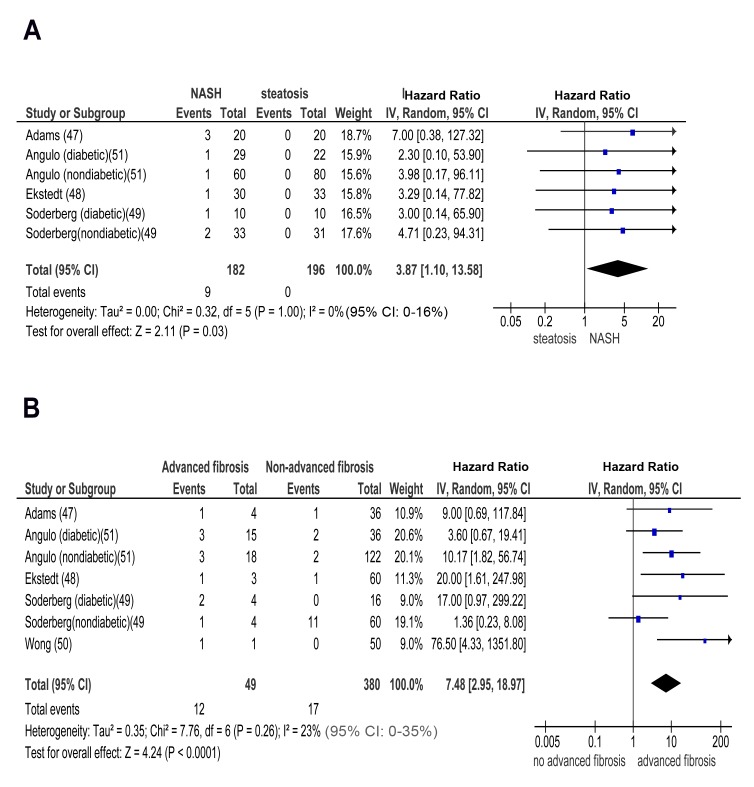
Forest plot of comparison. (A) NASH versus simple steatosis in biopsy-proven non-cirrhotic NAFLD; outcome: incident CKD stage 5 (renal failure) in prospective studies. (B) Advanced (stage F3) fibrosis versus no advanced (stage F0–F2) fibrosis in biopsy-proven non-cirrhotic NAFLD; outcome: incident CKD stage 3b in prospective studies.

In longitudinal studies, pooled HR for CKD stage 3b, 4, and 5 (renal failure) was significantly higher in NASH versus steatosis: OR for CKD stage 3b: 2.49 (95% CI 1.21–5.13, I^2^ = 0% [95% CI 0%–21%], n-comparisons = 7, *p* = 0.01); OR for CKD stage 4: 3.45 (95% CI 1.15–10.39, I^2^ = 0% [95% CI 0%–18%], n-comparisons = 6, *p* = 0.03); OR for CKD stage 5: 3.87 (95% CI 1.10–13.58, I^2^ = 0% [95% CI 0%–16%], n-comparisons = 6, *p* = 0.03) (Figures 6 and 7).

**Figure 8:**
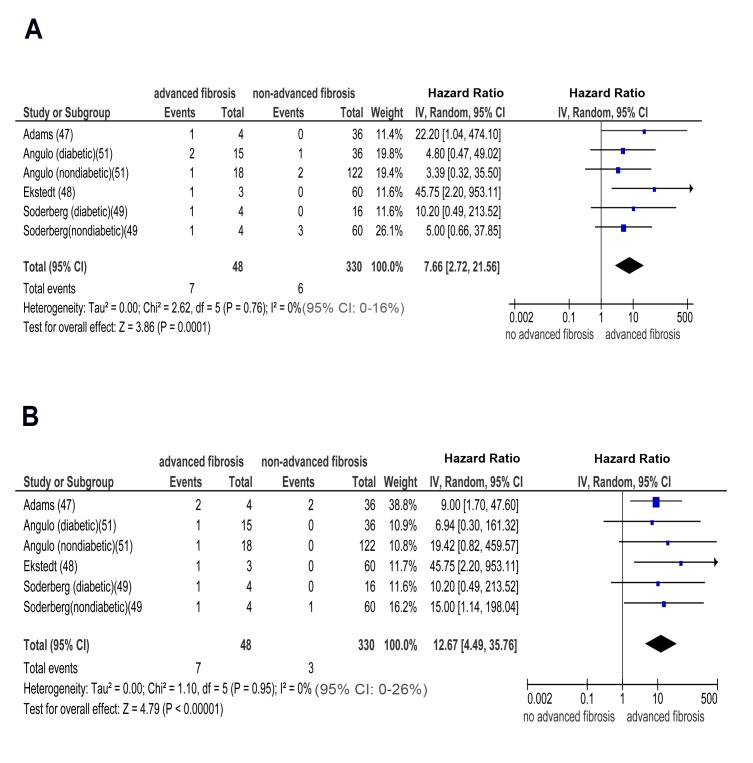
Forest plot of comparison. (A) advanced (stage F3) fibrosis versus no advanced (stage F0–F2) fibrosis in biopsy-proven non-cirrhotic NAFLD; outcome: incident CKD stage 4 in prospective studies. (B) Advanced (stage F3) fibrosis versus no advanced (stage F0–F2) fibrosis in biopsy-proven non-cirrhotic NAFLD; outcome: incident (CKD) stage 5 (renal failure) in prospective studies.

Similarly, pooled HR for CKD stage 3b, 4, and 5 (renal failure) was significantly higher in advanced versus non-advanced fibrosis: OR for CKD stage 3b: 7.48 (95% CI 2.95–18.97, I^2^ = 23% [95% CI 0%–35%], n-comparisons = 7, *p*<0.0001); OR for CKD stage 4: 7.66 (95% CI 2.72–21.56, I^2^ = 0% [95% CI 0%–16%], n-comparisons = 6, *p* = 0.0001); OR for CKD stage 5: 12.67 (95% CI 4.49–35.76, I2 = 0% [95% CI 0%–26%], n-comparisons = 6, *p*<0.00001) (Figures 7 and 8).

There was no heterogeneity in the meta-analysis of overall events, suggesting a consistent disease effect.

### Subgroup Analyses

#### NAFLD and prevalent/incident CKD

The magnitude and direction of the associations were unaltered across studies fulfilling different STROBE score items in non-diabetic individuals (Figures S10–S14 and S23–S26 within Text S3) versus diabetic individuals (Figures S15 and S27 within Text S3), when the analysis was restricted to studies adjusting for age and BMI and metabolic syndrome and hypertension and smoking status (Figures S16 and S28 within Text S3), in population-based versus hospital-based studies (Figures S17 and S29 within Text S3), in studies including Asian versus non-Asian individuals (Figures S18 and S30 within Text S3), in studies using CKD-EPI versus studies using the MDRD equation (Figures S20 and S31 within Text S3), after exclusion of studies assessing only eGFR or proteinuria (Figures S21 and S32 within Text S3), and in studies providing IPD versus studies providing exclusively AD (Figures S22 and S33 within Text S3). Furthermore, the main results remained largely unaltered after excluding the only cross-sectional study including cirrhotic individuals (Figure S19 within Text S3), while no prospective study enrolled subjects with cirrhosis at baseline. Subgroup analyses are summarized in Table 3.

### NAFLD Histological Subtypes and the Risk of CKD

#### NASH/advanced fibrosis and prevalent CKD

The magnitude and direction of the effect were unaltered across studies fulfilling different STROBE score items (Figures S34 and S38 within Text S3) in non-diabetic versus diabetic individuals (Figures S35 and S39 within Text S3), in studies including Asian versus non-Asian individuals (Figures S36 and S40 within Text S3), in studies using CKD-EPI versus studies using the MDRD equation (Figure S41 within Text S3), after exclusion of studies assessing only eGFR or proteinuria (Figures S37 and S42 within Text S3), and in studies providing IPD versus studies providing exclusively AD (Figure S43 within Text S3).

All studies adjusted for traditional risk factors for CKD, were hospital-based and enrolled non-cirrhotic patients.

#### NASH/advanced fibrosis and incident CKD

The magnitude and direction of the effect remained unaltered in non-diabetic versus diabetic individuals (Figures S44 and S47 within Text S3), in Asian versus non-Asian individuals (Figures S45 and S48 within Text S3) and after exclusion of studies assessing only eGFR (Figures S46 and S49 within Text S3). All studies satisfied all STROBE score items, were hospital-based, enrolled non-cirrhotic individuals, adjusted for traditional risk factors for CKD, and used CKD-EPI equation to estimate GFR.

### One-Stage Individual Participant Data Meta-analysis

Twenty studies (29,282 participants, 11 cross-sectional studies, nine longitudinal studies) were included in this analysis. We first analyzed the influence of each single pre-specified individual patient level covariate on the association of NAFLD with CKD with NAFLD and covariate as fixed-effect and the study as random-effects. In a second step, we did a complete case multivariable analysis with respect to NAFLD and all pre-specified covariates. The covariates entered in the models were age, BMI, metabolic syndrome (presence versus absence), diabetes (presence versus absence), hypertension (presence versus absence), smoking status (current smokers versus non-smokers), ethnicity (Asian versus non-Asian population), cirrhosis (presence versus absence), waist circumference, HOMA-index, duration of follow-up (for longitudinal studies).

The magnitude of the effect of NAFLD on CKD remained largely unaffected after adjusting for the covariates separately and in the fully adjusted models (Table 4).

**Table 4:** Adjusted effect estimates for non-alcoholic fatty liver disease, non-alcoholic steato-hepatitis, advanced (stage F3) fibrosis and prevalent/incident chronic kidney disease, based on individual participant data meta-analysis from 20 studies (29,282 participants).

Outcome	Cross-sectional Studies			Longitudinal Studies		
Covariate	Participants	OR (95% CI)	*p*-Value	Participants	HR (95% CI)	*p*-Value
**CKD in NAFLD vs. non-NAFLD**						
**Diabetes**	507 diabetic 3,031 non-diabetic	2.62 (1.95–3.57)	0.00001	2,046 diabetic, 39,365 non-diabetic	1.99 (1.83–2.25)	0.00001
**Age (y)** a	51 (18–89)	2.39 (1.93–2.99)	0.00003	38 (20–80)	2.10 (1.74–2.63)	0.000006
**BMI (kg/m^2^)** a	26 (16–71)	2.21 (1.85–2.67)	0.00006	24 (16–47)	2.40 (1.94–2.99)	0.00001
**Met sy**	885 with Met Sy, 2,654 without Met Sy	2.43 (1.95–3.06)	0.00001	3,758 with Met Sy, 19,226 without Met Sy	2.13 (1.77–2.71)	0.00009
**HTN**	1,061 with HTN, 2,477 without HTN)	2.19 (1.78–2.61)	0.00002	4,367 with HTN, 18,617 without HTN	2.01 (1.69–2.77)	0.00002
**Smoking**	1,168 smokers, 2,370 non-smokers	2.11 (1.75–2.58)	0.00005	9,653 smokers, 13,331 non-smokers	2.28 (1.87–2.89)	0.00008
**Ethnicity**	2,867 Asians, 1,071 non-Asian	2.25 (1.87–2.70)	0.00002	18,519 Asians, 6,937 non-Asians	2.32 (1.32–4.10)	0.00004
**Cirrhosis**	2,976 non-cirrhotic, 562 cirrhotic^b^	2.34 (1.92–2.85)	0.00001	No cirrhotic participant	**—**	**—**
**Waist (cm)** a	91 (54–153)	2.63 (2.02–3.12)	0.00007	89 (51–150)	2.52 (2.00–3.02)	0.00007
**HOMA-IR** a	1.8 (1.0–15.1)	2.55 (2.00–2.98)	0.00001	1.6 (0.6–10.3)	2.12 (1.76–2.54)	0.0009
**Duration of follow-up** a	**—**	**—**	**—**	5 (1–29)	2.48 (1.98–2.97)	0.0001
**All covariates**	3,538 individuals	1.95 (1.55–2.71)	0.00001	22,984 individuals	1.91 (1.68–2.21	0.00001
**CKD in NASH vs. simple steatosis**						
**Diabetes**	119 diabetic, 769 non-diabetic	2.77 (1.81–4.24)	0.0004	96 diabetic, 333 non-diabetic	2.32 (1.55–3.48)	0.00009
**Age (y)** a	46 (18–80)	3.01 (2.50–3.72)	0.00001	47 (18–67)	2.61 (1.71–3.24)	0.00004
**BMI (kg/m^2^)** a	33 (18–59)	2.78 (2.09–3.24)	0.0003	27 (18–47)	2.32 (1.59–3.15)	0.00006
**Met sy**	355 with Mey Sy, 532 without Met Sy	2.66 (2.05–3.12)	0.00008	136 with Met Sy, 293 without Met Sy	2.71 (2.18–3.59)	0.0001
**HTN**	408 with HTN, 479 without HTN	2.59 (1.97–3.18)	0.00002	172 with HTN, 257 without HTN	2.58 (1.99–3.11)	0.00007
**Smoking**	222 smokers, 665 non-smokers	2.69 (1.63–3.82)	0.00001	103 smokers, 326 non-smokers	2.56 (1.54–3.13)	0.0002
**Ethnicity**	263 Asians, 624 non-Asians	3.14 (2.44–4.96)	0.00001	102 Asians, 327 non-Asians	2.37 (1.41–3.98)	0.0001
**Cirrhosis**	No cirrhotic participant	—	—	No cirrhotic participant	—	—
**Waist (cm)** a	110 (51–162)	2.62 (2.01–3.31)	0.0006	99 (70–115)	2.76 (2.01–3.48)	0.0003
**HOMA-IR** a	3.1 (0.3–28.1)	2.54 (1.98–2.96)	0.00002	2.7 (0.8–3.9)	2.24 (1.55–2.46)	0.00001
**Duration of follow-up** a	—	—	—	14 (3–30)	2.31 (1.42–2.87)	0.00007
**All covariates**	887 individuals	2.42 (1.80–3.84)	0.0001	429 individuals	2.01 (1.40–2.98)	0.0001
**CKD in advanced (stage F3) vs. non-advanced (stage F0–F2) fibrosis**						
**Diabetes**	119 diabetic, 769 non-diabetic	5.39 (3.66–8.20)	0.0001	96 diabetic, 333 non-diabetic	3.88 (2.97–5.23)	0.00001
**Age (y)** a	46 (18–80)	5.12 (3.71–6.99)	0.00009	47 (18–67)	3.91 (2.56–4.98)	0.00007
**BMI (kg/m^2^)** a	33 (18–59)	4.89 (3.94–5.98)	0.00001	27 (18–47)	3.45 (2.51–4.02)	0.00004
**Met sy**	355 with Mey Sy, 532 without Met Sy	5.00 (4.11–5.97)	0.00007	136 with Met Sy, 293 without Met Sy	3.28 (2.39–4.52)	0.00006
**HTN**	408 with HTN, 479 without HTN	4.98 (3.81–6.02)	0.0003	172 with HTN, 257 without HTN	4.11 (2.88–5.51)	0.0001
**Smoking**	222 smokers, 665 non-smokers	5.27 (3.94–6.42)	0.00008	103 smokers, 326 non-smokers	3.29 (2.24–4.43)	0.00002
**Ethnicity**	263 Asians, 624 non-Asians	5.18 (3.57–6.82)	0.00001	102 Asians, 327 non-Asians	3.70 (1.46–9.39)	0.0006
**Cirrhosis**	No cirrhotic participant	—	—	No cirrhotic participant	—	—
**Waist (cm)** a	110 (51–162)	5.39 (4.21–6.13)	0.0006	99 (70–115)	3.39 (2.12–4.51)	0.00001
**HOMA-IR** a	3.1 (0.3–28.1)	5.13 (4.01–6.29)	0.00004	2.7 (0.8–3.9)	3.70 (2.12–4.91)	0.00003
**Duration of follow-up** a	—	—	—	14 (3–30)	3.58 (2.46–5.89)	0.0009
**All covariates**	887 individuals	4.86 (3.54–6.69)	0.00001	429 participants	3.00 (2.08–4.33)	0.0001

Data from all studies providing IPD were pooled together into a single dataset and effect estimates were calculated using multivariate logistic regression (cross-sectional studies) or Cox proportional hazard models (longitudinal studies). In these models, studies were incorporated as cluster and treated as random-effect, while covariates were treated as fixed-effect. The individual patient covariates entered in the models were: age, BMI, metabolic syndrome, hypertension, smoking status, diabetes, ethnicity (Asian versus non-Asian population), presence of cirrhosis, waist circumference, HOMA-IR index, duration of follow-up (for longitudinal studies). Finally, a fully adjusted model was run, with all covariates entered.

HTN, hypertension; Met Sy, metabolic syndrome; waist, waist circumference.

a For continuous variables, median (range) of values is reported.

b All cirrhotic individuals derive from the study by Park et al. [30].

## Discussion

The main results of our analysis are the following: (1) NAFLD was associated with an increased prevalence and incidence of CKD; (2) liver disease severity in NAFLD was associated with an increased risk and severity of CKD; (3) these associations remained statistically significant in diabetic and non-diabetic individuals, as well as in studies adjusting for traditional risk factors for CKD, and were independent of whole body/abdominal obesity and insulin resistance.

The prevalence of CKD is rapidly growing and in the United States over 1.1 million individuals are estimated to have ESRD by the year 2015 [60]. In addition to progressing to ESRD, CKD is also a major risk factor for CVD, and most individuals with CKD die from CVD before they develop ESRD. Therefore, the search for modifiable risk factors for CKD is attracting much attention.

NAFLD is an emerging risk factor for end-stage liver disease and CVD: the frequency of NASH as the primary indication for liver transplantation has increased from 1.2% to 9.7% in the last decade, becoming the third most common indication for liver transplantation in the United States [61]. Furthermore, the number of combined liver and kidney transplants has been increasing exponentially in the last 5 years [62], thereby challenging cost-effective resource utilization in the treatment of end-stage organ disease. For these reasons, establishing a link between liver and kidney injury would enhance earlier identification of kidney disease and allow for the selection of treatments targeting both liver disease and CKD progression in individuals with NAFLD, with potentially relevant preventive and therapeutic implications. Our analysis disclosed an association between the presence and severity of NAFLD and the risk and severity of CKD. This association remained robust in cross-sectional and longitudinal studies, across different definitions (imaging, histology, biochemistry) of NAFLD and after taking different confounders into account. Notably, heterogeneity across cross-sectional studies evaluating NAFLD by ultrasound was abated after excluding data from analysis of the NHANES III 1988–1994 cohort, which failed to find an association between NAFLD and CKD [40]. This finding may be at least partially explained by the protocol used in that study: NHANES III was not originally designed to study hepatic steatosis and the authors diagnosed NAFLD retrospectively, on the basis of archived videotapes of gallbladder ultrasound examinations. In 2009–2010, trained ultrasound readers examined the protocol used in that study and found only modest intra- and inter-reliability for the presence of hepatic steatosis, i.e., 0.77 (95% CI 0.73–0.82) and 0.70 (95% CI 0.64–0.76), respectively [63]–[65]. This flaw may have further diluted the disease effect on CKD through misclassification of NAFLD cases as non-NAFLD, since mild steatosis, often present in progressive NASH and advanced fibrosis, is frequently missed by ultrasound.

### Implications for Practice

Current guidelines do not recommend screening for CKD in the absence of traditional risk factors for CKD [66]. Our data suggest that individuals with NAFLD should be screened for CKD by estimation of GFR and urinalysis even in the absence of classical risk factors for CKD, particularly if NASH and/or advanced fibrosis are suspected. Early recognition of impaired kidney function in NAFLD may also allow drug dosage adjustment, thus preventing drug accumulation, especially in those being treated for obesity-associated comorbidities.

From a therapeutic standpoint, there is a considerable potential for improving the current care of NAFLD patients with CKD: with respect to lifestyle interventions, smoking cessation should be more vigorously pursued, as cigarette smoking is an established risk factor for CKD, and may also aggravate NAFLD [67],[68]. Among pharmacological options, preliminary data from the GREACE and FANTASY randomized trials suggest statins and angiotensin receptor blockers (ARBs) may improve both liver and kidney disease in NAFLD [56],[69]–[71]. Beside statins and ARBs, other agents, including pentoxifylline and ω-3 polyunsaturated fatty acids, improved surrogate markers of NAFLD and CKD in distinct NALFD-associated settings like obesity, diabetes, and hypertension, and their impact on CKD in NAFLD warrants future assessment [72]–[76].

### Implications for Research

Further research is required to unravel the specific cascades linking NAFLD and kidney disease. NAFLD and CKD share common risk factors and therefore both liver and kidney injury may be driven by obesity-associated mechanisms of disease, including lipotoxicity, oxidative stress, enhanced pro-inflammatory cytokine, and renin-angiotensin-aldosterone system (RAAS) axis activation [10],[77]–[80]. However, our analysis of longitudinal studies suggests NAFLD may promote CKD independently of coexisting risk factors. Consistently, recent data suggest the steatotic and inflamed liver may be a relevant source of pro-inflammatory, pro-fibrogenic, and anti-fibrinolytic molecules, including fetuin-A, fibroblast growth factor (FGF)-21, tumor necrosis factor (TNF)-α, transforming growth factor (TGF)-β, and plasminogen activator inhibitor-1, all having the ability to promote kidney injury [81]–[85]. Furthermore, fatty liver may damage the kidney through VLDL lipoprotein over-secretion and induction of atherogenic dyslipidemia [86],[87], as triglyceride-rich lipoproteins and oxidized LDLs promote glomerular injury and mesangial cell proliferation [77].

We also found a cross-sectional association between NAFLD and CKD, implying that even mild renal dysfunction may promote liver disease, in a mutual negative loop with detrimental cardio-metabolic consequences Consistently, uni-nephrectomized rats developed body fat redistribution from adipose depots to non-adipose tissues, profound dysregulation of hepatic fatty acid metabolism, steatohepatitis, insulin resistance, hyperglycaemia, and dyslipidaemia early after uni-nephrectomy and long before glomerulosclerosis and chronic renal failure occur [88],[89]. Notably, all renal and metabolic changes were prevented by angiotensin converting enzyme (ACE) inhibitors in this experimental model, indicating the involvement of the RAAS [74].

Lastly, since NAFLD is an emerging risk factor for liver-related and cardiovascular complications, the hypothesis that the presence of CKD may represent a simple, cost-effective tool to predict increased liver-related and CVD risk in NAFLD is intriguing and warrants assessment in large-scale prospective studies. Currently, in fact, there is no validated non-invasive marker to predict the risk of both liver disease progression and future CVD, the main causes of death in NAFLD patients [9].

Our findings may also have therapeutic research implications. Future trials will need to evaluate the impact of experimental treatments on kidney-related outcomes. Notably, only two of the available randomized controlled trials in NAFLD reports the effect of drugs on eGFR and proteinuria and none has adequate size and duration to evaluate the impact of treatments on kidney-related clinical outcomes.

Our analysis has limitations, which are intrinsic to the nature of included studies and provide the basis for future research. Studies with biopsy-proven NAFLD were by their own nature less numerous and smaller than those adopting ultrasonographic/biochemical definitions of NAFLD, leaving the possibility of small study bias that is not detected by current tests. Furthermore, these studies were performed in tertiary centers with the possibility of selection bias. Conversely, ultrasound/liver enzyme elevations are relatively insensitive to detect NAFLD, with possible misclassification of individuals with NASH/advanced fibrosis as healthy controls and underestimation of the strength of the association between NAFLD and CKD. However, there was no heterogeneity between studies: disease effect on CKD.

Finally, despite our best efforts IPD were unavailable from as much as 39% of relevant studies, representing 54% of participant population. While meta-analysis of AD has several limitations, including ecological bias and study-level confounding, excluding such a substantial proportion of relevant literature from our analysis would have raised the concern of data availability bias [90]. Furthermore, the methodological quality of the 13 studies providing exclusive AD was generally good, 77% of them adjusted for all potential confounders, and 69% of them allowed separate risk estimates for individual patient level covariates (including diabetes, cirrhosis, ethnicity).

Balancing all these reasons, we presented all relevant literature evidence by combining IPD with AD in the main analysis, and separately analyzed studies providing IPD with a one-stage method: notably, the two analyses yielded similar results, further supporting the robustness of overall findings.

In conclusion, our analysis shows that the presence and severity of NAFLD are associated with an increased risk and severity of CKD and may be a target for the prevention and treatment of CKD. Future research should evaluate strategies and interventions to prevent renal disease progression in individuals with NAFLD.

## Supporting Information

Text S1 **PRISMA checklist.**
(PDF)

Text S2 **Search strategies and protocol.**
(PDF)

Text S3 **Additional analyses.**
(DOC)

## Abbreviations

AD: aggregate data
BMI: body mass index
CKD: chronic kidney disease
CVD: cardiovascular disease
ESRD: end-stage renal disease
GFR: glomerular filtration rate
eGFR: estimated glomerular filtration rate
HOMA-IR: homeostasis model assessment of insulin resistance
HR: hazard ratio
IPD: individual participant data
MDRD: Modified Diet in Renal Disease
NAFLD: non-alcoholic fatty liver disease
NASH: non-alcoholic steatohepatitis
OR: odds ratio
