# Supplementary material for: Association of Non-alcoholic Fatty Liver Disease with Chronic Kidney Disease: A Systematic Review and Meta-analysis
Source: PLoS Med. 2014 Jul 22;11(7):e1001680. doi: 10.1371/journal.pmed.1001680 (PMC4106719; doi:10.1371/journal.pmed.1001680)
Supplement: Text S2 — Search strategies and protocol. (PDF) [file pmed.1001680.s002.pdf]

Online Appendix 1

Content:

-literature search strategies in different databases

-study protocol

Literature search strategies in different databases

Online search strategy run in MEDLINE database:

“chronic kidney disease” [All fields] OR “kidney, disease”[MeSH Terms] OR “renal”[All fields] AND “disease”[All fields] AND “chronic” [All fields] OR “renal”[All fields] AND “insufficiency”[All fields] OR “kidney”[All fields] AND “failure” [All fields] OR “kidney”[All fields] AND “damage” [All fields] OR “kidney”[All fields] AND “injury” [All fields] OR “glomerular filtration rate”[All fields] OR “eGFR”[All fields] OR “creatinine” [All fields] OR “proteinuria”[All fields] OR “albuminuria”[All fields] OR “microalbuminuria”[All fields] OR “macroalbuminuria”[All fields] OR “renal” [All fields] AND “function” [All fields] AND “decreased”[All fields] OR “impaired renal function” [All fields] OR “chronic kidney failure” [MeSH Terms] OR “Renal Replacement”[All fields] OR “dialysis” [All fields] OR “emodialysis” [All fields] OR “emodiafiltration” [All fields] OR “emofiltration” [All fields]

AND

“liver”[MeSH Terms] OR “liver”[All fields] AND “fat” [All fields] OR “fatty liver” [All fields] OR “fatty liver” [MeSH Terms] OR “non-alcoholic” [All fields] OR “steatosis” [All fields] OR “steatosis” [MeSH Terms] OR steatohepatitis” [All fields] OR “steatohepatitis” [MeSH Terms] OR “NAFLD” [All fields] OR “NAFLD” [MeSH Terms] OR “NASH” [All fields] OR “NASH” [MeSH Terms] OR “liver histology”

[Subheading] OR “enzymes” [All fields] OR “enzymes” [MeSH Terms] OR “transaminases”[All fields] OR “transaminases” [MeSH Terms] OR “enzymology”[Subheading] OR “enzymology” [All fields]

Online search strategy run in EMBASE database:

"chronic kidney disease" OR "chronic" AND "kidney" AND "disease" OR

"chronic" AND "renal" AND "disease" OR

"chronic kidney disease" NEAR/5 "chronic" NEAR/5 "kidney" NEAR/5 "disease" OR

"chronic" NEAR/5 "renal" NEAR/5 "disease" OR

"renal" AND "insufficiency" OR

"renal" NEAR/5 "insufficiency" OR

"kidney"[All fields] AND "failure" [All fields] OR

"kidney"[All fields] NEAR/5 "failure" [All fields] OR

"kidney" AND "damage" OR

"kidney" NEAR/5 "damage" OR

"kidney" AND "injury" OR

"kidney" NEAR/5 "injury" OR

OR "glomerular filtration rate" OR "eGFR" OR "creatinine" OR "proteinuria" OR "albuminuria" OR

"microalbuminuria" OR "macroalbuminuria" OR "decreased renal function" OR

"renal" AND "function" AND "decreased" OR

"renal" NEAR/5 "function" NEAR/5 "decreased" OR

"impaired renal function" OR "impaired" AND "function" AND "renal" OR

"impaired" NEAR/5 "function" NEAR/5 "renal" OR

"Renal Replacement" OR "Renal" AND "Replacement" OR "Renal" NEAR/5 "Replacement" OR

"dialysis" OR "emodialysis" OR "emodiafiltration" OR "emofiltration"

AND

"liver" OR "liver" AND "fat" OR "fatty liver" OR "fatty" NEAR/5 "liver" OR

"non-alcoholic" OR "steatosis" OR "non-alcoholic" AND "steatosis" OR "non-alcoholic" NEAR/5 "steatosis" OR "non-alcoholic" NEAR/5 "liver"OR

"non-alcoholic" AND "steatohepatitis" OR "non-alcoholic" NEAR/5 "steatohepatitis" OR

"NAFLD" OR "NASH" OR "liver histology" OR "liver" AND histology" OR "liver" NEAR/5 histology" OR "transaminases" OR "gammaglutamyl-transferase" OR "imaging" OR "liver" AND "imaging" OR "liver" NEAR/5 "imaging" OR "liver" AND "ultrasound" OR "liver" NEAR/5 "ultrasound" OR ""liver" AND "enzymes" OR "liver" NEAR/5 "enzymnes" OR "enzymology"

Online search strategy run in Ovid MEDLINE In-Process

"chronic kidney disease" OR "kidney, disease" OR "renal" AND "disease" AND "chronic" OR "renal" AND "insufficiency" OR "kidney" AND "failure" OR "kidney" AND "damage" OR "kidney" AND "injury" OR "glomerular filtration rate" OR "eGFR" OR "creatinine" OR "proteinuria" OR "albuminuria" OR "microalbuminuria" OR "renal" AND "function" AND "decreased" OR "impaired renal function" OR "chronic kidney failure" OR "Renal Replacement" OR "dialysis" OR "emodialysis" OR "emodiafiltration" OR "emofiltration"

AND

"liver" OR "liver" AND "fat" OR "fatty liver" OR "fatty liver" OR "non-alcoholic" OR "steatosis" OR steatohepatitis" OR "NAFLD" OR "NASH" OR "liver histology" OR "enzymes" OR "enzymes" OR "transaminases" OR "transaminases" OR "enzymology" OR "enzymology".

Online search strategy run in ISI Web of Science

"chronic kidney disease" OR "chronic" AND "kidney" AND "disease" OR "chronic" AND "renal" AND "disease" OR "chronic kidney disease" NEAR/5 "chronic" NEAR/5 "kidney" NEAR/5 "disease" OR "chronic" NEAR/5 "renal" NEAR/5 "disease" OR "renal" AND "insufficiency" OR "renal" NEAR/5 "insufficiency" OR "kidney" AND "failure" OR "kidney" NEAR/5 "failure" [All fields] OR "kidney" AND "damage" OR "kidney" NEAR/5 "damage" OR

"kidney" AND "injury" OR "kidney" NEAR/5 "injury" OR "glomerular filtration rate" OR "eGFR" OR "creatinine" OR "proteinuria" OR "albuminuria" OR "microalbuminuria" OR "macroalbuminuria" OR "decreased renal function" OR "renal" AND "function" AND "decreased" OR

"renal" NEAR/5 "function" NEAR/5 "decreased" OR "impaired renal function" OR "impaired" AND "function" AND "renal" OR "impaired" NEAR/5 "function" NEAR/5 "renal" OR

"Renal Replacement" OR "Renal" AND "Replacement" OR "Renal" NEAR/5 "Replacement" OR

"dialysis" OR "emodialysis" OR "emodiafiltration" OR "emofiltration"

AND

"liver" OR "liver" AND "fat" OR "fatty liver" OR "fatty" NEAR/5 "liver" OR

"non-alcoholic" OR "steatosis" OR "non-alcoholic" AND "steatosis" OR "non-alcoholic" NEAR/5 "steatosis" OR "non-alcoholic" NEAR/5 "liver"OR

"non-alcoholic" AND "steatohepatitis" OR "non-alcoholic" NEAR/5 "steatohepatitis" OR "NAFLD" OR "NASH" OR "liver histology" OR "liver" AND histology" OR "liver" NEAR/5 histology" OR "transaminases" OR "gammaglutamyl-transferase" OR "imaging" OR "liver" AND "imaging" OR "liver" NEAR/5 "imaging" OR "liver" AND "ultrasound" OR "liver" NEAR/5 "ultrasound" OR

""liver" AND "enzymes" OR "liver" NEAR/5 "enzymnes" OR "enzymology"

Online search strategy run in Cochrane Library (all sections)

#1

"liver" OR "liver" AND "fat" OR "fatty liver" OR "non-alcoholic" OR "steatosis" OR "non-alcoholic" AND "steatosis" OR "non-alcoholic" AND "steatohepatitis" OR "NAFLD" OR "NASH" OR "liver histology" OR "liver" AND histology" OR "transaminases" OR "gammaglutamyl-transferase" OR "liver" AND "imaging" OR "liver" AND "ultrasound" OR "liver" AND "radiology" OR ""liver" AND "enzymes" OR ""liver" AND "disease"

#2

"chronic kidney disease" OR "chronic" AND "kidney" AND "disease" OR "chronic" AND "renal" AND "disease" OR "chronic kidney disease" OR "renal" AND "insufficiency" OR "kidney" AND "failure" OR "kidney" AND "damage" OR "kidney" NEAR/5 "damage" OR "kidney" AND "injury" OR "glomerular filtration rate" OR "eGFR" OR "creatinine" OR "proteinuria" OR "albuminuria" OR "microalbuminuria" OR "macroalbuminuria" OR "decreased renal function" OR "renal" AND "function" AND "decreased" OR "impaired renal function" OR "impaired" AND "function" AND "renal" OR "Renal Replacement" OR "Renal" AND "Replacement" OR "dialysis" OR "emodialysis" OR "emodiafiltration" OR "emofiltration"

#3 (#1 AND #2)

Study protocol

BACKGROUND

Chronic kidney disease (CKD) is a common condition: it affects 4-13% of the Western adult population and its prevalence is continuously rising along with the epidemic of its risk factors, including ageing, diabetes, obesity, metabolic syndrome, smoking and hypertension [James MT, Lancet 2010; Stevens PE, Ann Int Med 2012, McCullough K, Nephrol Dial Transplant. 2012]. Beside being a risk factor for end-stage renal disease (ESRD), CKD is an important cardiovascular disease (CVD) risk factor, and most patients with CKD die from CVD before any renal replacement therapy is initiated [Herzog Ca, Kidney Int 2011]. Early recognition and treatment of CKD, particularly at its early stage (stage 3) aimed at reducing renal disease progression and CVD complications may effectively limit its health-related burden [James MT, Lancet 2010; Black C, Health Technol Assess 2010]. The high morbidity, mortality and health-care costs associated with CKD have led the investigators to seek novel modifiable risk factors. Non-alcoholic fatty liver disease (NAFLD), the hepatic manifestation of the metabolic syndrome, affects 30% of the general adult population and up to 70% of diabetic and obese patients [Chalasani N, Gastroenterology 2012]. NAFLD encompasses a histological spectrum ranging from simple steatosis to non-alcoholic steatohepatitis (NASH), the latter with or without advanced fibrosis. NAFLD confers an increased risk of cirrhosis, largely limited to NASH, and of CVD, independently of metabolic syndrome and traditional risk factors, through still unclear mechanisms [Musso G, Ann Med 2011]. Growing experimental and epidemiological evidence suggests that NAFLD and CKD share common pathogenic mechanisms and mutual interactions [Ix IH, JASN 2010].

WHY IS IMPORTANT TO DO THIS REVIEW

CKD is an emerging problem for public health. Its health-related burden could be alleviated if CKD could be recognized at its early stages (stage 3) and treated. NAFLD is a common condition, as well, whose prevalence is rapidly growing, along with the epidemic of its risk factors obesity and diabetes. Growing

experimental evidence connects NAFLD to CKD and suggests several therapeutic strategies may benefit both liver and kidney disease in NAFLD. However, current search for evidence of a pathogenic link between NAFLD and CKD is inconclusive due to small study populations and borderline associations between NAFLD and traditional risk factors for CKD.

O B J E C T I V E S

To meta-analyse the evidence regarding two research questions:

1) Does NAFLD affect the risk of CKD independently from major confounders?

2) Is NAFLD severity associated with the severity of CKD?

METHODS

Criteria for considering studies for this review

Types of studies

The review will include only observational studies.

Types of participants

To be included in the review, studies will need to be conducted on patients fulfilling the following criteria:

-NAFLD defined according to standard guidelines (Chalasani N, Gastroenterology 2012)

-CKD as defined to standard guidelines (Stevens PE, Ann Int Med 2012)

Types of outcome measures

Primary outcomes

Primary outcome measures will be differences in the prevalence or incidence of CKD. We will compare the risk of primary outcomes between NAFLD patients and individuals without NAFLD as well as across the main histological subtypes of NAFLD, as NASH and advanced fibrosis carry a significantly worse prognosis than steatosis and milder fibrosis stages, respectively. The impact of NAFLD and of NAFLD histological subtypes (NASH, advanced fibrosis) on eGFR, treated as a continuous variable, and on proteinuria, will also be examined.

Secondary outcomes

The impact of the severity of NAFLD, as defined by NASH or advanced fibrosis, on the severity of CKD, as defined by the stage of CKD, will be also assessed.

CKD stage will be categorized by GFR according to recent guidelines into CKD stage 3b (eGFR 30-44 ml/min/1.73 m^2, CKD stage 4 (eGFR 15-29 ml/min/1.73 m^2) and CKD stage 5 (eGFR<15 ml/min/1.73 m^2)[(Stevens PE, Ann Int Med 2012)].

Search methods for identification of studies

Electronic searches

We will perform a computerized literature search in:

MEDLINE, Ovid MEDLINE In-Process, EMBASE, ISI Web of Science, and Cochrane Library through January 2014. The search starts on November 2012 and will be re-run periodically every 3 month until January 2014.

Searching other resources

We will hand-search the abstracts from the annual American Association for the Study of Liver Disease (AASLD)(published in Hepatology), American Gastroenterological Association (AGA)(published in Gastroenterology), European Association for the Study of the Liver (EASL)(published in J Hepatology), Digestive Disease Week (DDW) (published in Gastroenterology and American Society of Nephrology (ASN) Kidney Week (published in J American society of Nephrology) meetings 2005 through January 2014. We asked the authors of study reports published only as abstracts or of ongoing studies to contribute IPD or completed papers.

Data collection and analysis

Selection of studies

Data will be extracted from each study independently and in duplicate by two authors (GM, RG), using a data extraction sheet based on the Cochrane Handbook for Systematic Reviews of Intervention They will assess title, keywords, and abstracts of all studies retrieved with the search strategies described above. If, based on this information, the authors believe studies meet the defined inclusion criteria, they will retrieve and further assess the full text and make a final decision on whether to include a study. Arbitration of a third author (MC) is foreseen in cases where one author believes a specific study meets the inclusion criteria for the review whereas the other author does not.

Data extraction and management

The review authors will use a standardized data extraction form to compile and document relevant facts on general study characteristics, methodological quality, patients' characteristics, and outcomes as specified above. This data extraction will be performed independently.

The data extraction form compiles the following items.

1. General information on the study: title, authors, contact address, funding sources, language, publication status, year of publication, place(s), and year(s) of study conduction.

2. Study design issues: methods used to define NAFLD and CKD, methodological quality according to the modified STROBE checklist, inclusion and exclusion criteria,

3. Loss to follow-up for longitudinal studies

4. Outcomes in each group: odds ratios (ORs) and confidence intervals (CIs) for cross-sectional studies, hazard ratios (HRs) and confidence intervals (CIs) for longitudinal studies

Consultation of a third author for arbitration, which is foreseen for cases where no consensus will be reached.

Individual patient data (IPD)

For all included studies, individual participant data (IPD) will be solicited from principal investigators (PIs). PIs will be asked to provide the most complete and updated data, even if the follow-up is longer than that used for respective publications. Contact information for study authors will be identified from PubMed or from the Internet and authors listed as contact authors will be e-mailed or contacted by phone

to tell them about our IPD meta-analysis, and to ask if they were willing to share their study data. Up to 3 tries within a month will be made: If there will be no response from the contact author, another investigator from the study will be contacted with the same modalities for up to 3 times in 1 month. If no response is obtained, a third investigator from the same study will be contacted with the same modalities. If we have no response after the attempts to contact 3 different investigators from the same study, we will assume those investigators were not interested in sharing the IPD from their study.

Data not available upon database closure (January 2014), either because IPD had not been provided or because full manuscripts had not been published, will not included in our analyses.

Type of individual participant data collection from studies:

The solicited databases from each study will be collected in a pre-specified excel spreadsheet with one subject per row, and variables listed in columns. If in doubt, we will contact primary study authors for clarification and/or discuss within the collaborative group. After data have been received, they will be stored on a secure institutional server.

Requested variables will be: age (at beginning of follow-up for longitudinal studies), gender, race, BMI, waist circumference, NAFLD (yes/no; specify mode of diagnosis), NASH (yes/no, specify criteria for diagnose NASH), fibrosis stage(range 0-4; exclude patients with stage 4 fibrosis, i.e. cirrhosis), AST, ALT, ALP, GGT, date of beginning of follow-up (for longitudinal studies), date of diagnosis of NAFLD, serum creatinine at the beginning of follow-up, proteinuria (present/absent) at the beginning of follow-up(specify method used to detect proteinuria), hypertension(yes/no) at the beginning of follow-up, diabetes(yes/no) at the beginning of follow-up, systolic BP, diastolic BP, HDL-C, triglyceride, LDL-C, total cholesterol, fasting plasma glucose, fasting serum insulin, HOMA-IR score, date of first diagnosis of CKD or of end of follow-up(for longitudinal studies), serum creatinine at first diagnosis of CKD or at the end of follow-up (for longitudinal studies), proteinuria (yes/no) at first diagnosis of CKD or at the end of follow-up (for longitudinal studies), smoking status (smoker/non-smoker), metabolic syndrome (yes/no), lost to follow up (yes/no).

Quality control of submitted IPD

We will assess the quality of the submitted IPD from the single studies in several ways.

1. We will compare the number of individual participant data sets with the population reported in publications.

2. We will screen data sets for obvious duplicates or omissions (e.g. by checking patient IDs).

3. For each participant, we will recalculate eGFR from raw data (age, gender, race, serum creatinine) using standard equations, will check methods and cut-offs for defining proteinuria and will compare our findings with those provided by investigators in each dataset

4. We will check plausibility of the values supplied for each variable by looking for extreme outliers.

5. We will assess missing observations for each variable and checked against the original publication.

6. We will compare summary measures calculated from the data set with data reported in publications, by reproducing the statistical methods as reported by the study authors.

7. We will discuss and clarify any discrepancies or missing information between our results and those presented in each original study

8. Once data checks are complete and satisfactory, individual study datasets will be combined to form a new master dataset with a variable added to indicate the original study.

Dealing with missing data

For missing data, we will contact the authors of the single studies and ask them for the specific values. Our database closed on 31 Jan 2014. Any data not available at that date, either because it was not provided by the PIs as IPD or because results of the respective trial had not been published as full manuscripts, will not be included in our analyses.

Measures of treatment effect

We will measure the effect of NAFLD on the risk of CKD using multivariate logistic regression in cross-sections studies and Cox proportional hazards model in longitudinal studies, obtaining odds ratios (ORs) and hazard ratios(HR) as measures of effect size. If possible the HR will be based on IPD. If IPD will not be available we will calculate the HR

1. from the published reports, using methods described in Parmar et al (Parmar 1998) and Tierney et al (Tierney 2007)

2. from binary data (CKD present/absent) at the end of follow-up.

For each longitudinal study we will estimate log HRs and the standard errors of log HR using the following methods (based on those reported by Parmar, Tierney, and Williamson; Parmar 1998; Tierney 2007; Williamson 2002), listed in order of preference:

1. HR and confidence interval calculated directly from IPD.

2. O-E and variance of the log hazard ratio: log hazard ratio and its standard error estimated directly.

3. HR reported with confidence interval or log-rank P value: standard error estimated from confidence interval or P value (confidence interval used if both available). This is the preferred indirect method since the HR is directly extracted and the standard error is estimated very accurately.

4. Adjusted HR reported with confidence interval or Cox proportional hazards P value: standard error estimated from confidence interval or P value (confidence interval used if both available). This will generally give an estimate close to the unadjusted HR, but different studies adjust for different factors, and the choice of adjustment factors could be data-driven, leading to bias.

5. Numbers of events reported with log-rank P value: HR estimated from numbers of events, standard error estimated from this estimated HR and P value. This gives a indirect estimate of the HR since all events are considered, but may not be close to the actual HR, particularly if the hazards are not proportional.

6. Kaplan-Meier survival curve.

7. Actuarial rates at fixed follow-up and log-rank P value. This gives an estimate of the HR similar to that of method (3), but only events up to the fixed follow-up time are considered.

When time-to-event data is unknown, we will use multivariate logistic regression analysis of binary data at the end of follow-up

Assessment of heterogeneity

We will assess heterogeneity clinically through the calculation of an I^2 (95%CIs calculated with the method provided by Higgins, Higgins stat Med 2002) statistic which is a measure for the percentage of the variability in effect estimates attributed to heterogeneity rather than sampling error. If heterogeneity between the effects found in single trials is shown to be too large, i.e. an I^2 above 0.5, we will explore the sources of heterogeneity.

Assessment of reporting biases

To assess possible publication bias, if the number of included studies is sufficient, we will create a funnel plot using the different outcomes and evaluated funnel asymmetry with Egger's test.

Data synthesis

For all outcomes, we planned to perform a meta-analysis based on exclusive IPD if the number of studies providing IPD is ≥90% of included studies; otherwise, we plan to combine IPD and aggregate data using the two-stage method (Riley 2007). This implies that from those studies for which IPD are available, we will calculate the outcome measure, as defined above, from the provided data. For studies where IPD will not be available, we will use the aggregate outcome measure derived from the pertinent publication. The estimated logORs or logHRs will combined using the generic inverse-variance method, the result of which will be presented as pooled OR (for cross-sectional studies)or HR (for longitudinal studies) with 95% confidence intervals. The pooled OR or HR represents the overall risk of disease (CKD) for NAFLD (or NAFLD histological subtypes) versus controls (i.e. non-NAFLD or other histological subtypes of NAFLD). We will use a random-effects model for all meta-analyses. The usage of a random-effects model is preferred to that of a fixed-effect model because we assume

the existence of non-explainable heterogeneity between the 'true' effects of the different treatment regimens implied in the studies. In all tests of significance we will calculate a two-sided P value. We will use RevMan 5.2 (Nordic Cochrane Center, Copenhagen, Denmark) and SAS 9.2 (SAS Institute. Cary, North Carolina, USA) for additional analyses that could not be done with RevMan.

Sensitivity analysis

For all outcomes, we will conduct sensitivity analyses by repeating the meta-analysis after one study at a time was removed to assess whether any one study significantly affected pooled estimates.

Subgroup analysis and investigation of heterogeneity

We will separately analyze cross-sectional and longitudinal studies; furthermore, for each outcome, the results of studies defining NAFLD by histology, imaging, or liver enzyme elevation will be presented separately, as these methods have different accuracy for detecting NAFLD..

We planned a number of subgroup analyses *a priori*. These subgroup analyses include repeated analysis after excluding studies not fulfilling each STROBE item, and separate analyses for the following items:

-diabetes: we will examine the effect of NAFLD on CKD in non-diabetic vs. diabetic individuals, to assess if the presence of diabetes affects the association of NAFLD with CKD.

-studies simultaneously adjusting vs. studies not adjusting for all the following risk factors for CKD: age, BMI, metabolic syndrome (overall or each of its components), hypertension, smoking status.

-study design (population-based vs. hospital-based)

-ethnicity (Asian vs. non-Asian population), to explore whether differences in risk factors and epidemiology of NAFLD and CKD between Asian and non-Asians populations affect the association of NAFLD with CKD.

-studies enrolling exclusively non-cirrhotic patients vs. studies enrolling exclusively cirrhotic patients

-methods used to estimate GFR

-outcomes related to CKD: studies assessing both eGFR and proteinuria vs. studies assessing solely eGFR or proteinuria

-study data availability: IPD vs. AD.

Additionally, for the primary end-point we will separately perform a one-stage meta-analysis of studies providing IPD, to examine how the association of NAFLD with CKD is altered when individual patient level covariates are accounted for. In this analysis, data from all studies providing IPD will be pooled together into a single dataset and effect estimates will be calculated using multivariate logistic regression (cross-sectional studies) or Cox proportional hazard models (longitudinal studies). In these models, studies will be incorporated as cluster and treated as random-effect, while covariates are treated as fixed-effect. The covariates entered in the models will be age, BMI, metabolic syndrome, diabetes, hypertension, smoking status. ethnicity (Asian vs. non-Asian population), presence of cirrhosis, waist circumference, HOMA-index, duration of follow-up (for longitudinal studies. We will first analyze the influence of each single pre-specified covariates on the association of NAFLD with CKD with NAFLD and covariate as fixed-effect and the study as random-effects. In a second step, we will do a complete case multivariable analysis with respect to NAFLD and all pre-specified covariates.

Meta-regression analysis

When ≥8 comparisons will be available, the effect of age, whole-body and abdominal obesity (as estimated by body mass index, BMI, and by waist circumference), insulin resistance (estimated by homeostasis model assessment of insulin resistance, HOMA-IR index), and of duration of follow-up (for longitudinal studies) on the association between NAFLD and CKD will be evaluated by meta-regression analysis (random effects model, within-study variance estimated with the unrestricted maximum-likelihood method).

DEFINITIONS USED IN THE META-ANALYSIS

CKD: diagnosis according to the Kidney Disease: Improving Global Outcomes (KDIGO) organization Guidelines (Ann Intern Med. 2013;158:825-830): either an eGFR<60 mL/min/1.73 m2 or proteinuria (see below)

CKD stage:

CKD stage 3a (eGFR 45-59 ml/min/1.73 m²,

CKD stage 3b(eGFR 30-44 ml/min/1.73 m²,

CKD stage 4(eGFR 15-29 ml/min/1.73 m^2)

CKD stage 5(eGFR<15 ml/min/1.73 m^2).

Proteinuria. tick the box according to the method you used to define proteinuria:

dipstick proteinuria □ ≥ +1 on fresh morning urine

albumin/creatinine ratio: □<30-300 mg/g □ <300 mg/g

albumin excretion rate □30-300 mg/d, □>300 mg/d,

Metabolic syndrome: metabolic syndrome (according to the joint statement of AHA, IDF and NHLBI Circulation. 2009;120:1640-5), requires the presence of ≥3 of the following criteria:

-abdominal obesity: waist circumference≥102 cm(males) and ≥88 cm(females)

-high triglycerides: ≥150 mg/dL or on drug treatment for elevated triglycerides

-low HDL-C: <40 mg/dL (males) or <50 mg/dL (females) or on drug treatment for reduced HDL-C

-hypertension: systolic BP≥130 and/or diastolic BP≥85 mm Hg or on drug treatment

-high fasting plasma glucose (FPG): FPG ≥100 mg/dL or on drug treatment for elevated glucose

Hypertension (JNC-7, Hypertension 2003 ;42:1206-52): systolic BP≥ 140 mmHg and/or diastolic BP≥90 mmHg on tow or more occasions or on antihypertensive drugs

Diabetes: diagnosis made with the 2013 American Diabetes Association guidelines fasting plasma glucose≥126 mg/dL or 2h-OGTT plasma glucose >200 mg/dl or Hba1c≥6.5% or on antidiabetic medications

Cirrhosis clinical or histological diagnosis (American Association for the Study of Liver Disease guidelines, Hepatology. 2009 ;49:2087-107
