# Supplementary material for: Association of Non-alcoholic Fatty Liver Disease with Chronic Kidney Disease: A Systematic Review and Meta-analysis
Source: PLoS Med. 2014 Jul 22;11(7):e1001680. doi: 10.1371/journal.pmed.1001680 (PMC4106719; doi:10.1371/journal.pmed.1001680)

**Online Appendix 2: additional analyses**

**Figure S1:** fulfilment of modified STROBE score criteria by included studies

The X axis report the 22 items of modified STROBE score; the Y axis the percentage of studies included in the analysis that fulfilled the criterion specified in the X axis.

**Modified STROBE score**:

(a) title and abstract informative and balanced;

(b) background/rationale stated in the introduction

(c ) objective(s) specified in the introduction

(d) study design correctly and presented early in the paper

(e) setting, locations, and relevant dates described

(f ) eligibility criteria, methods of selection and follow-up described

(g) diagnostic criteria, outcomes, exposures, predictors, potential confounders, and effect modifiers

for all variables clearly defined. Specifically, regarding the definition of NAFLD

-for radiological assessment: radiological exam performed by radiologists blinded to

clinical data and following pre-specified, standardized criteria to detect steatosis

-for histological assessment of NAFLD: adequate biopsy specimen (fragment length≥1.5 cm with

>6 portal tracts) and liver biopsy processed and scored by blinded pathologist according to

standard criteria

(h) sources of data and details of methods of measurement given for each variable of interest

(i) any efforts to address potential sources of bias described

(j) how the study size was arrived at clearly explained

(k) how quantitative variables were handled in the analyses clearly explained

(l) all statistical methods, how missing data and loss to follow-up were addressed, any sensitivity analyses clearly described

(m) numbers of individuals at each stage of study reported

(n) characteristics of study participants, number of participants with missing data, average and total

follow-up time clearly described

(o) outcome events or summary measures over time reported

(p) unadjusted and confounder-adjusted estimates and their precision (eg, 95% CI) reported

(q) analyses of subgroups and interactions, and sensitivity analyses reported

(r) key results with reference to study objectives summarised

(s) limitations of the study discussed

(t) cautious overall interpretation of results given

(u) generalizability (external validity) of the study results discussed

(v) source of funding and role of the funders described

**Figure S2A**. Forest plot of comparison: NAFLD vs. no-NAFLD, outcome: eGFR in cross sectional studies. NAFLD was defined by imaging, histology or liver enzyme elevation

(95% CI: 64-72%)

(95% CI: 0-6%)

(95% CI: 76-88%)

(95% CI: 90.2-97.1%)

**Figure S2B**. Forest plot of comparison: NAFLD vs. non-NAFLD, outcome: prevalent proteinuria

in cross-sectional studies. NAFLD was defined by imaging, histology or liver enzyme elevation.

(95% CI: 69.2-82.1%)

(95% CI: 69-83%)

(95% CI: 0-8%)

(95% CI: 70-87%)

**Figure S3A**. Forest plot of comparison: NAFLD vs. no-NAFLD, outcome: eGFR changes in longitudinal studies. NAFLD was defined by imaging, histology or liver enzyme elevation

(95% CI: 90-98%)

(95% CI: 90-98%)

(95% CI: 78.2-88.1%)

**Figure S3B**. Forest plot of comparison: NAFLD vs. non-NAFLD, outcome: incident proteinuria

in longitudinal studies. NAFLD was defined by imaging, histology or liver enzyme elevation.

**Hazard Ratio**

**Hazard Ratio**

(95% CI: 27-48%)

(95% CI: 0-12%)

(95% CI: 22-40%)

(95% CI: 31.1-42.0%)

**Figure S4A**. Funnel plot of comparison: NAFLD vs. non-NAFLD, outcome: prevalent chronic kidney disease (CKD) in cross-sectional studies. Oblique diverging lines represent pseudo 95% confidence limits. The empty diamond represents the observed Log odds ratio; the black filled diamond represents the adjusted Log odds ratio after the trim and fill procedure. The black dots represent the studies that had to be imputed during the same procedure.

Egger’s test

p-value = 0.473

Observed OR (95%CI): 2.12 (1.69-2.66)

Adjusted OR (95%CI): 2.01 (1.67-2.60)

Studies trimmed: 2

**Figure S4B**. Funnel plot of comparison: NAFLD vs. non-NAFLD, outcome: incident chronic kidney disease (CKD) in longitudinal studies. Oblique diverging lines represent pseudo 95% confidence limits. The empty diamond represents the observed Log odds ratio; the black filled diamond represents the adjusted Log odds ratio after the trim and fill procedure.

Egger’s test

p-value = 0.644

**Log hazard ratio**

**Funnel Plot of Standard Error by Log hazard ratio**

Observed HR (95%CI): 1.79 (1.65-1.95)

Adjusted HR (95%CI): 1.79 (1.65-1.95)

Studies trimmed: 0

**Figure S4C**. Funnel plot of comparison: NASH vs. steatosis, outcome: prevalent chronic kidney disease (CKD) in cross-sectional studies. Oblique diverging lines represent pseudo 95% confidence limits. The empty diamond represents the observed Log odds ratio; the black filled diamond represents the adjusted Log odds ratio after the trim and fill procedure. The black dot represents the study that had to be imputed during the same procedure.

Egger’s test

p-value = 0.346

Observed OR (95%CI): 2.53 (1.58-4.05)

Adjusted OR (95%CI): 2.39 (1.51-3.78)

Studies trimmed: 1

**Figure S4D**. Funnel plot of comparison: advanced fibrosis vs. no-advanced fibrosis, outcome: prevalent chronic kidney disease (CKD) in cross-sectional studies. Oblique diverging lines represent pseudo 95% confidence limits. The empty diamond represents the observed Log odds ratio; the black filled diamond represents the adjusted Log odds ratio after the trim and fill procedure. The black dot represents the study that had to be imputed during the same procedure.

Egger’s test

p-value = 0.126

Observed OR (95%CI): 5.20 (3.14-8.61)

Adjusted OR (95%CI): 4.80 (2.96-7.75)

Studies trimmed: 1

**Figure S4E**. Funnel plot of comparison: NASH vs. steatosis, outcome: incident chronic kidney disease (CKD) in longitudinal studies. Oblique diverging lines represent pseudo 95% confidence limits. The empty diamond represents the observed Log hazard ratio; the black filled diamond represents the adjusted Log hazard ratio after the trim and fill procedure.

Egger’s test

p-value = 0.309

**Funnel Plot of Standard Error by Log hazard ratio**

**Log hazard ratio**

Observed HR (95%CI): 2.12 (1.42-3.17)

Adjusted HR (95%CI): 2.12 (1.42-3.17)

Studies trimmed: 0

**Figure S4F**. Funnel plot of comparison: advanced fibrosis vs. no-advanced fibrosis, outcome: incident chronic kidney disease (CKD) in longitudinal studies. Oblique diverging lines represent pseudo 95% confidence limits. The empty diamond represents the observed Log hazard ratio; the black filled diamond represents the adjusted Log hazard ratio after the trim and fill procedure.

Egger’s test

p-value = 0.248

**Funnel Plot of Standard Error by Log hazard ratio**

**Log hazard ratio**

Observed HR (95%CI): 3.29 (2.30-4.71)

Adjusted HR (95%CI): 3.29 (2.30-4.71)

Studies trimmed: 0

**Figure S5A**. Forest plot of comparison: NASH vs. simple steatosis in biopsy-proven NAFLD; outcome: prevalent proteinuria in cross-sectional studies.

(95% CI: 0-12%)

**Figure S5B**. Forest plot of comparison: NASH vs. simple steatosis in biopsy-proven NAFLD; outcome: eGFR in cross-sectional studies.

(95% CI: 0-15%)

**Figure S6A**. Forest plot of comparison: advanced (stage F3) fibrosis vs. not-advanced (stage F0-2) fibrosis in biopsy-proven NAFLD, outcome: prevalent proteinuria in cross-sectional studies.

(95% CI: 0-16%)

**Figure S6B**. Forest plot of comparison: advanced (stage F3) fibrosis vs. not-advanced (stage F0-2) fibrosis in biopsy-proven NAFLD, outcome: eGFR in cross-sectional studies

(95% CI: 0-15%)

**Figure S7A**. Forest plot of comparison: NASH vs. simple steatosis in biopsy-proven NAFLD; outcome: incident proteinuria in longitudinal studies.

**Hazard Ratio**

**Hazard Ratio**

(95% CI: 0-14%)

**Figure S7B**. Forest plot of comparison: NASH vs. simple steatosis in biopsy-proven NAFLD; outcome: eGFR changes in longitudinal studies.

(95% CI: 0-11%)

**Figure S8A**. Forest plot of comparison: advanced (stage F3) fibrosis vs. not-advanced (stage F0-2) fibrosis in biopsy-proven NAFLD, outcome: incident proteinuria in longitudinal studies.

**Hazard Ratio**

**Hazard Ratio**

(95% CI: 30-48%)

**Figure S8B** . Forest plot of comparison: advanced (stage F3) fibrosis vs. not-advanced (stage F0-2) fibrosis in biopsy-proven NAFLD, outcome: eGFR changes in longitudinal studies.

(95% CI: 0-9%)

**Figure S9A**. Forest plot of comparison: NASH vs. simple steatosis in biopsy-proven NAFLD; outcome: prevalent chronic kidney disease (CKD) stage 3b in cross-sectional studies.

(95% CI: 0-17%)

**Figure S9B**. Forest plot of comparison: advanced (stage F3) fibrosis vs. no-advanced (stage F0-2) fibrosis in biopsy-proven NAFLD, outcome: prevalent chronic kidney disease (CKD) stage 3b in cross-sectional studies.

(95% CI: 0-21%)

**Figure S10.** Forest plots of subgroup analyses for the outcome: prevalent chronic kidney disease (CKD) in cross-sectional studies. STROBE score item (g) fulfilment

**
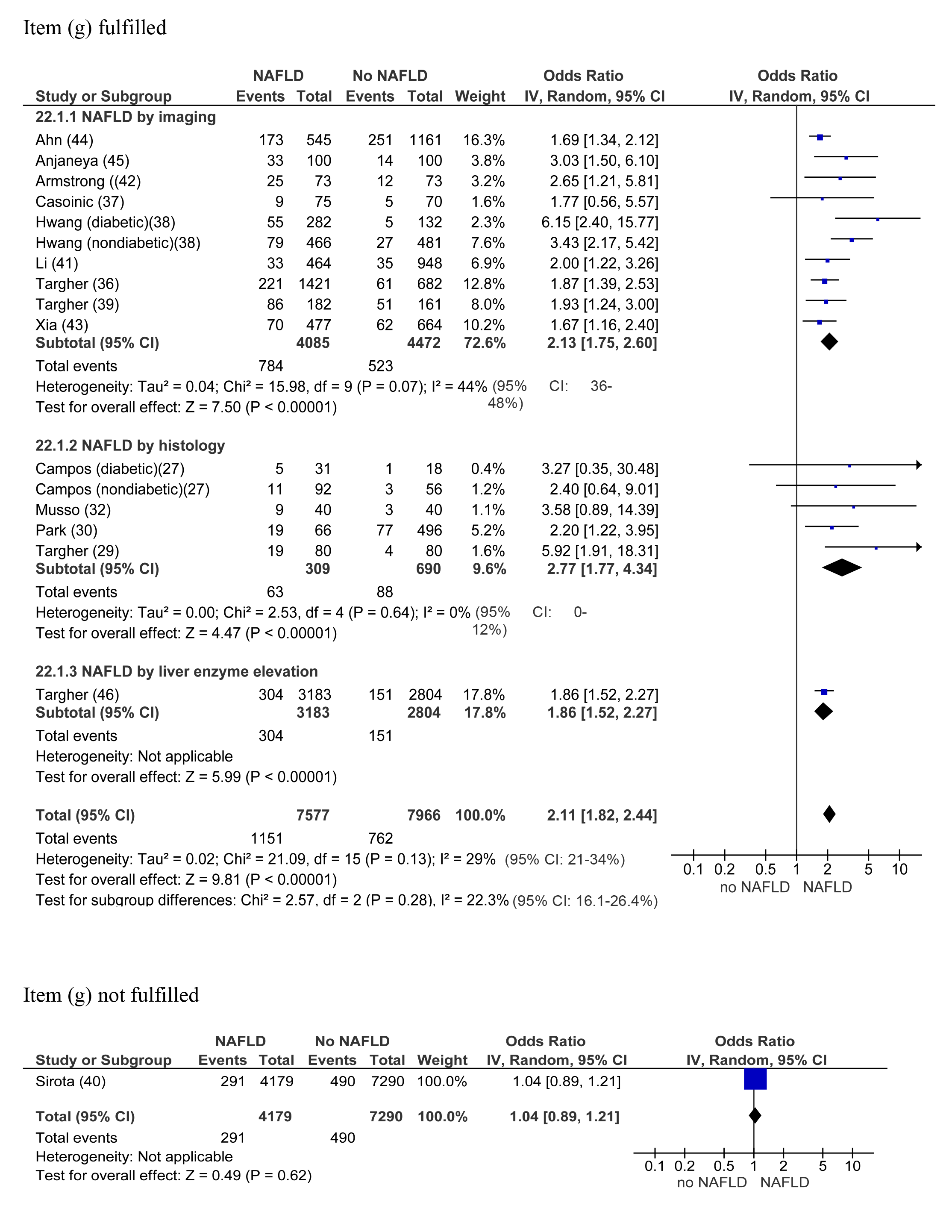
**

**Figure S11.** Forest plots of subgroup analyses for the outcome: prevalent chronic kidney disease (CKD) in cross-sectional studies. STROBE score item (p) fulfilment


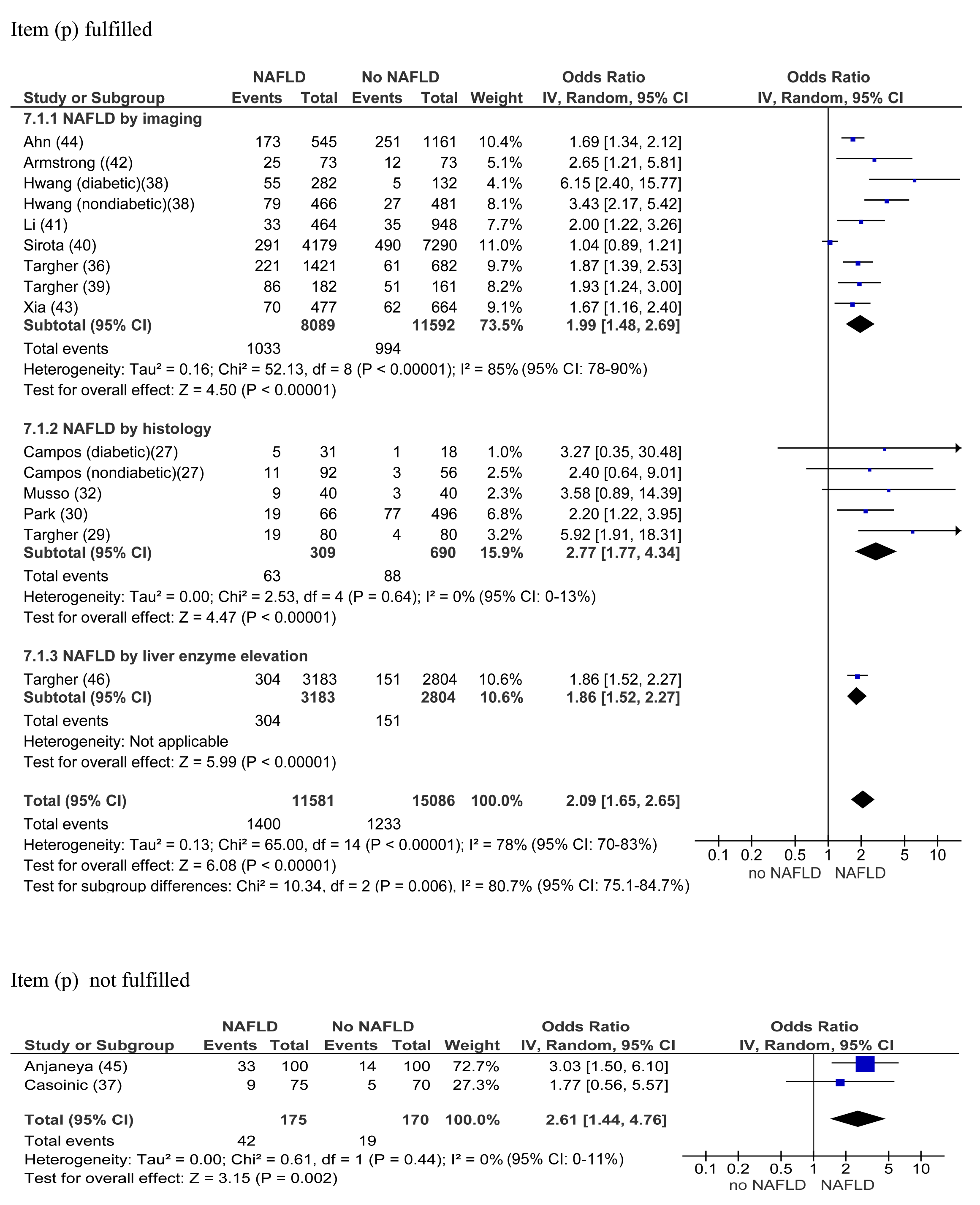


**Figure S12.** Forest plots of subgroup analyses for the outcome: prevalent chronic kidney disease (CKD) in cross-sectional studies. STROBE score item (s) fulfilment


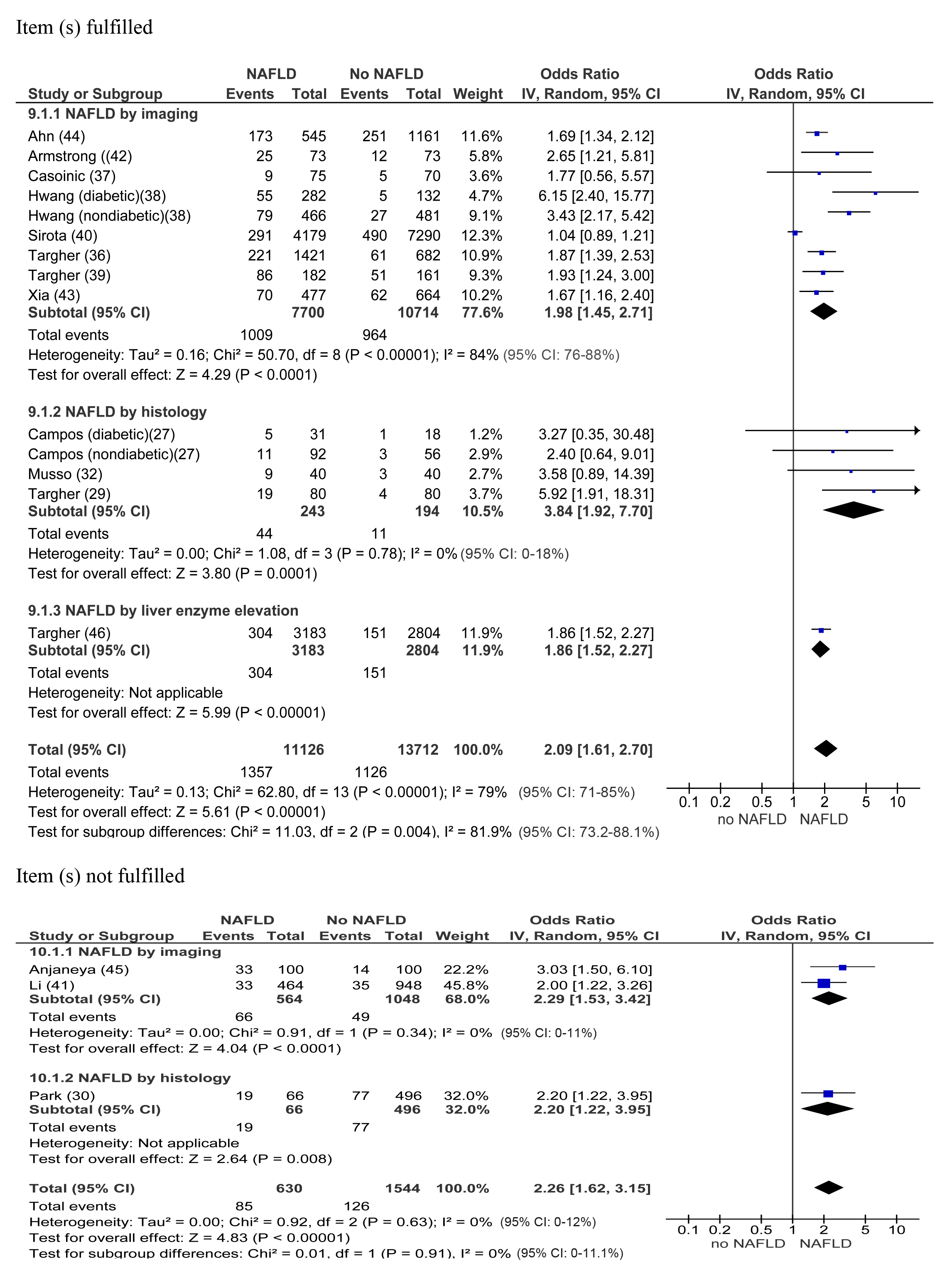


**Figure S13.** Forest plots of subgroup analyses for the outcome: prevalent chronic kidney disease (CKD) in cross-sectional studies. STROBE score item (t) fulfilment


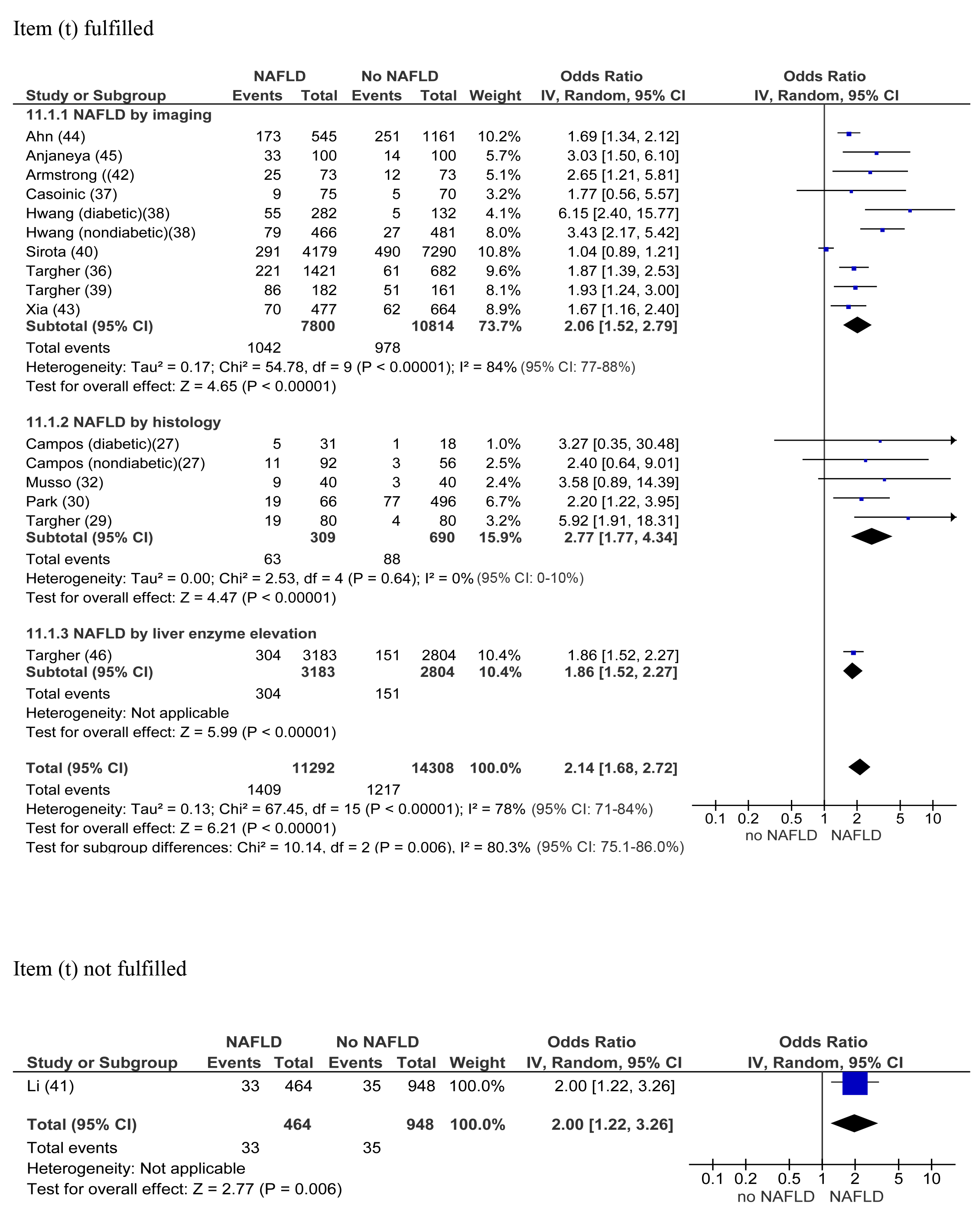


**Figure S14.** Forest plots of subgroup analyses for the outcome: prevalent chronic kidney disease (CKD) in cross-sectional studies. STROBE score item (v) fulfilment


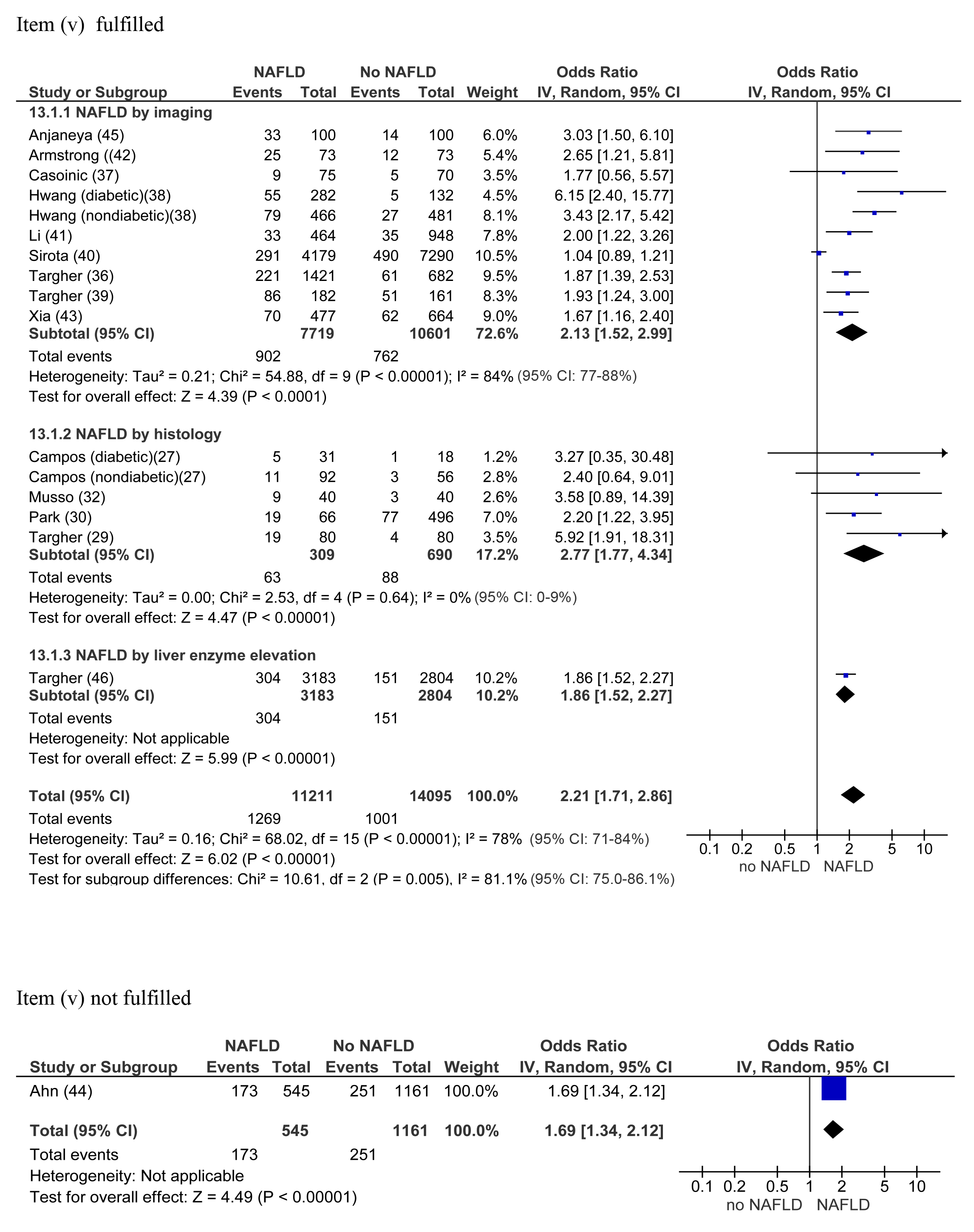


**Figure S15.** Forest plots of subgroup analyses for the outcome: prevalent chronic kidney disease (CKD) in cross-sectional studies. Presence of diabetes.


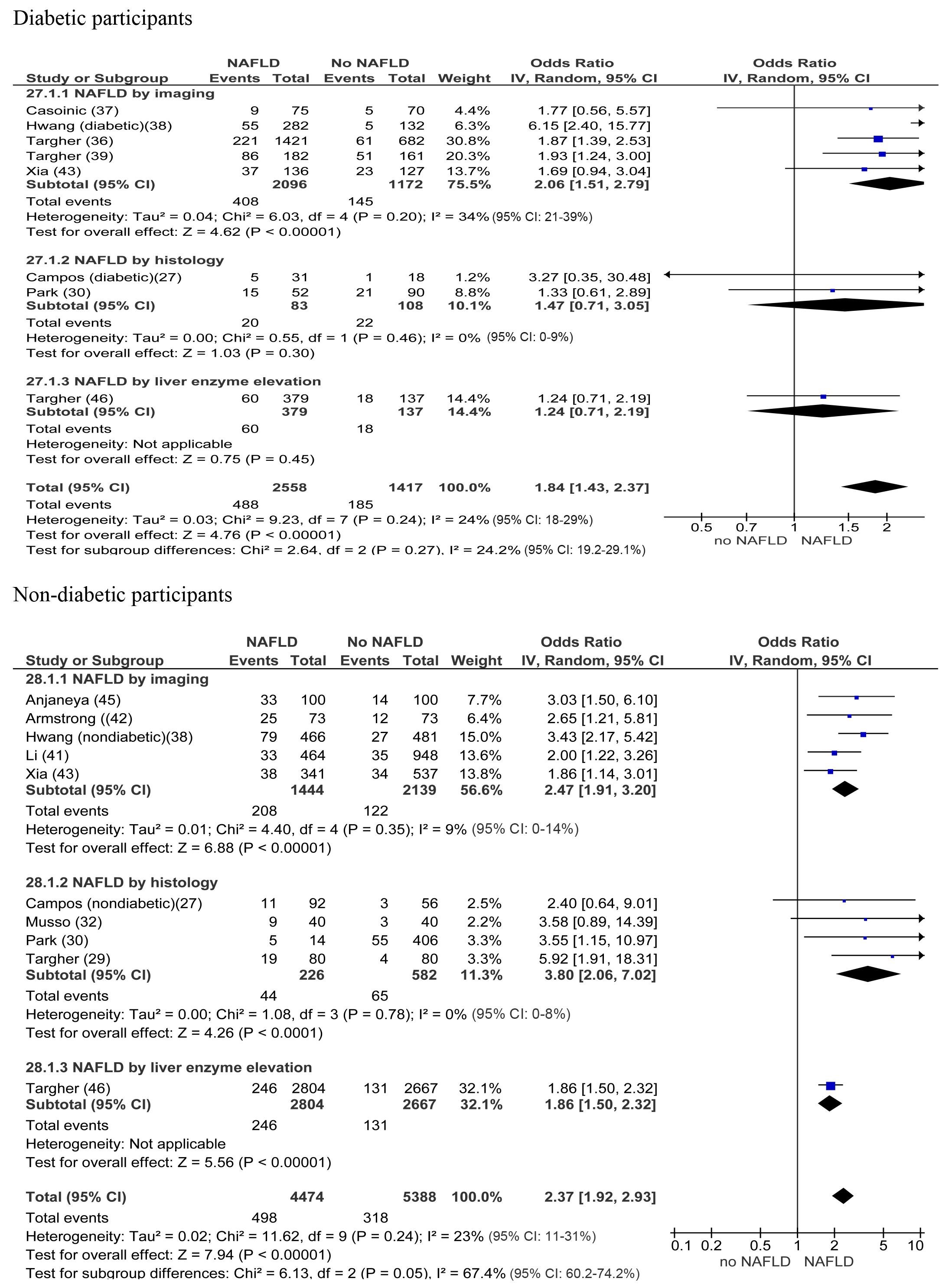


**Figure S16.** Forest plots of subgroup analyses for the outcome: prevalent chronic kidney disease (CKD) in cross-sectional studies. Adjustment for age and BMI and metabolic syndrome and hypertension and smoking.


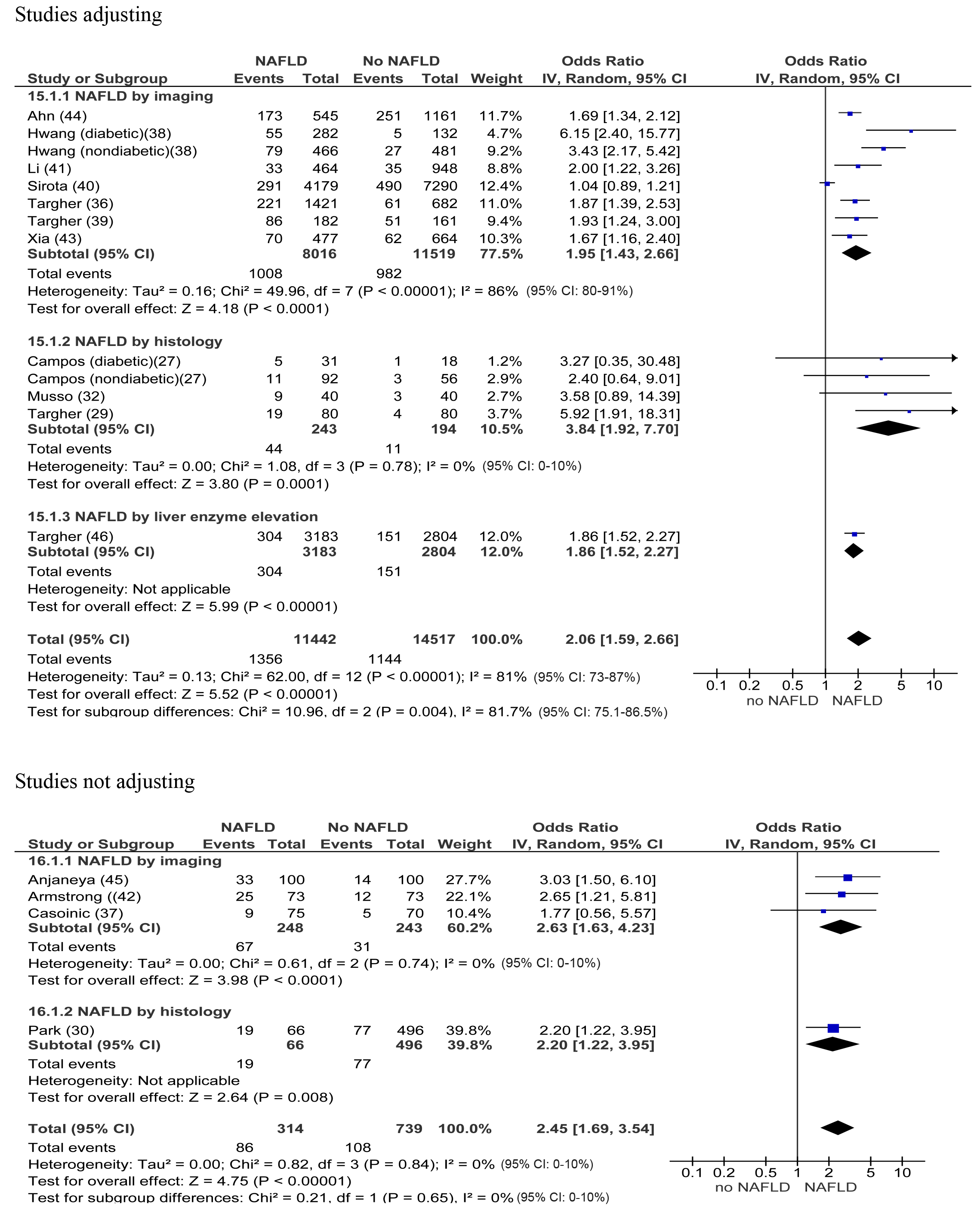


**Figure S17.** Forest plots of subgroup analyses for the outcome: prevalent chronic kidney disease (CKD) in cross-sectional studies. Study design: population-based vs. hospital-based


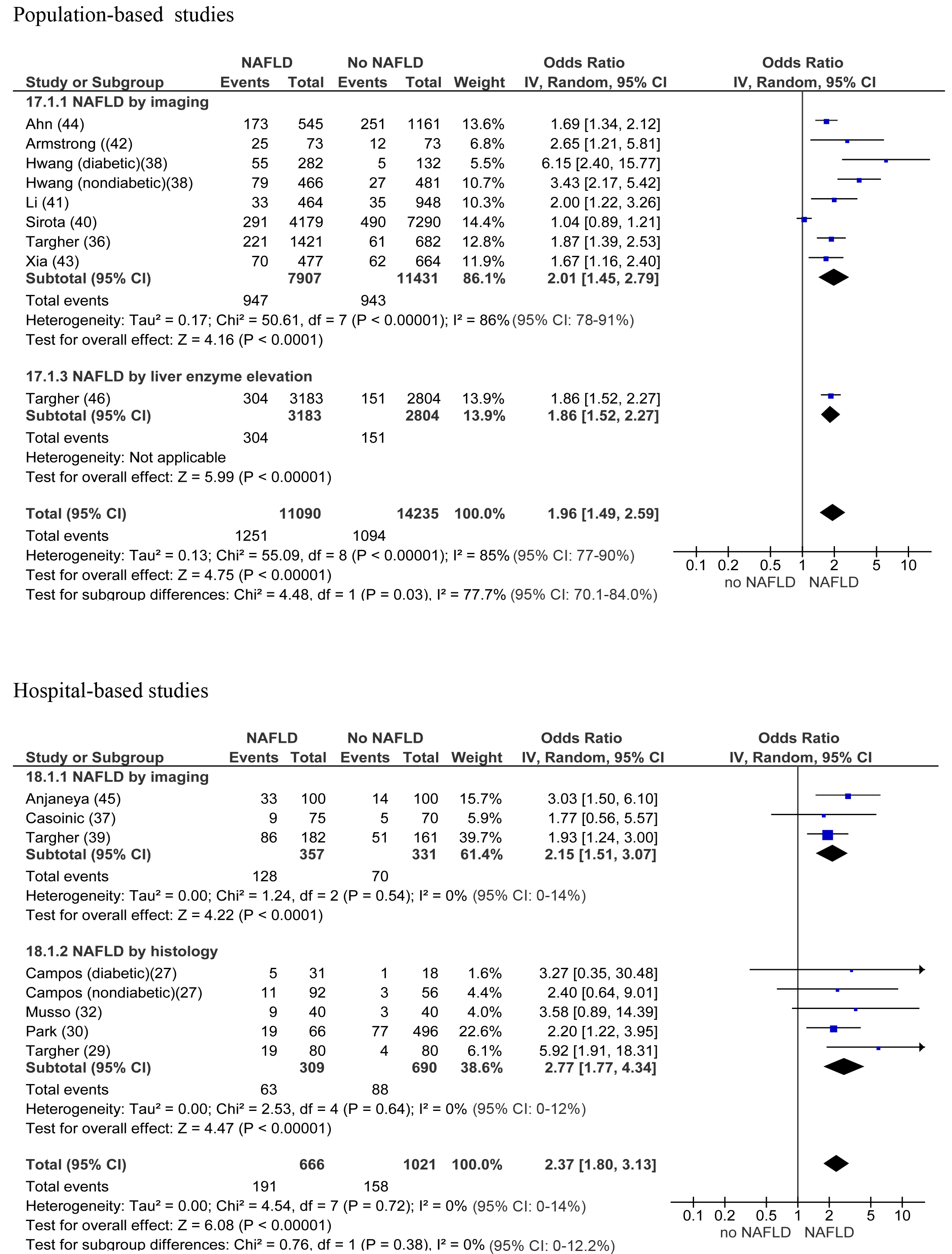


**Figure S18.** Forest plots of subgroup analyses for the outcome: prevalent chronic kidney disease (CKD) in cross-sectional studies. Ethnicity: Asian vs. non-Asian participants


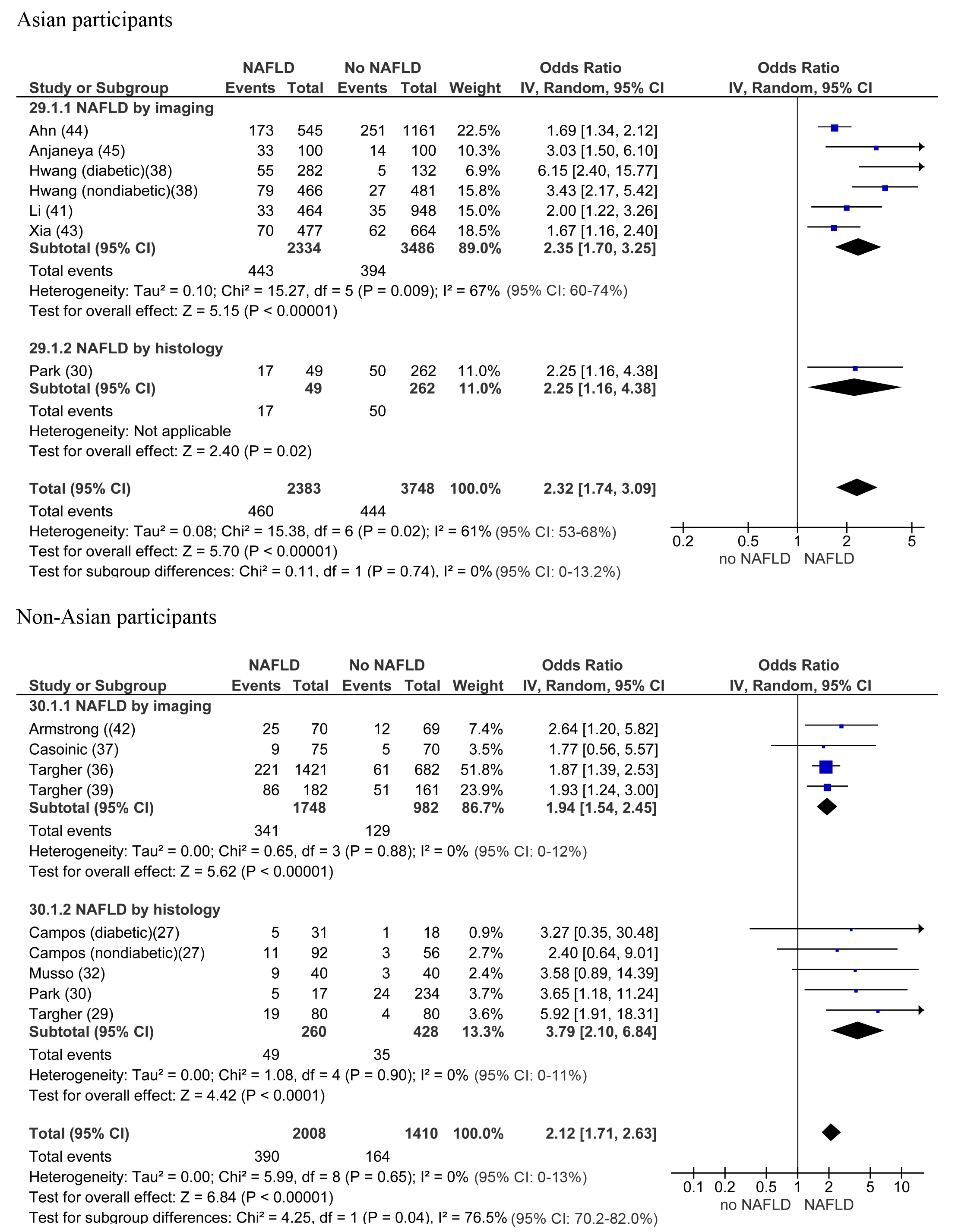


**Figure S19.** Forest plots of subgroup analyses for the outcome: prevalent chronic kidney disease (CKD) in cross-sectional studies. Presence of cirrhosis.


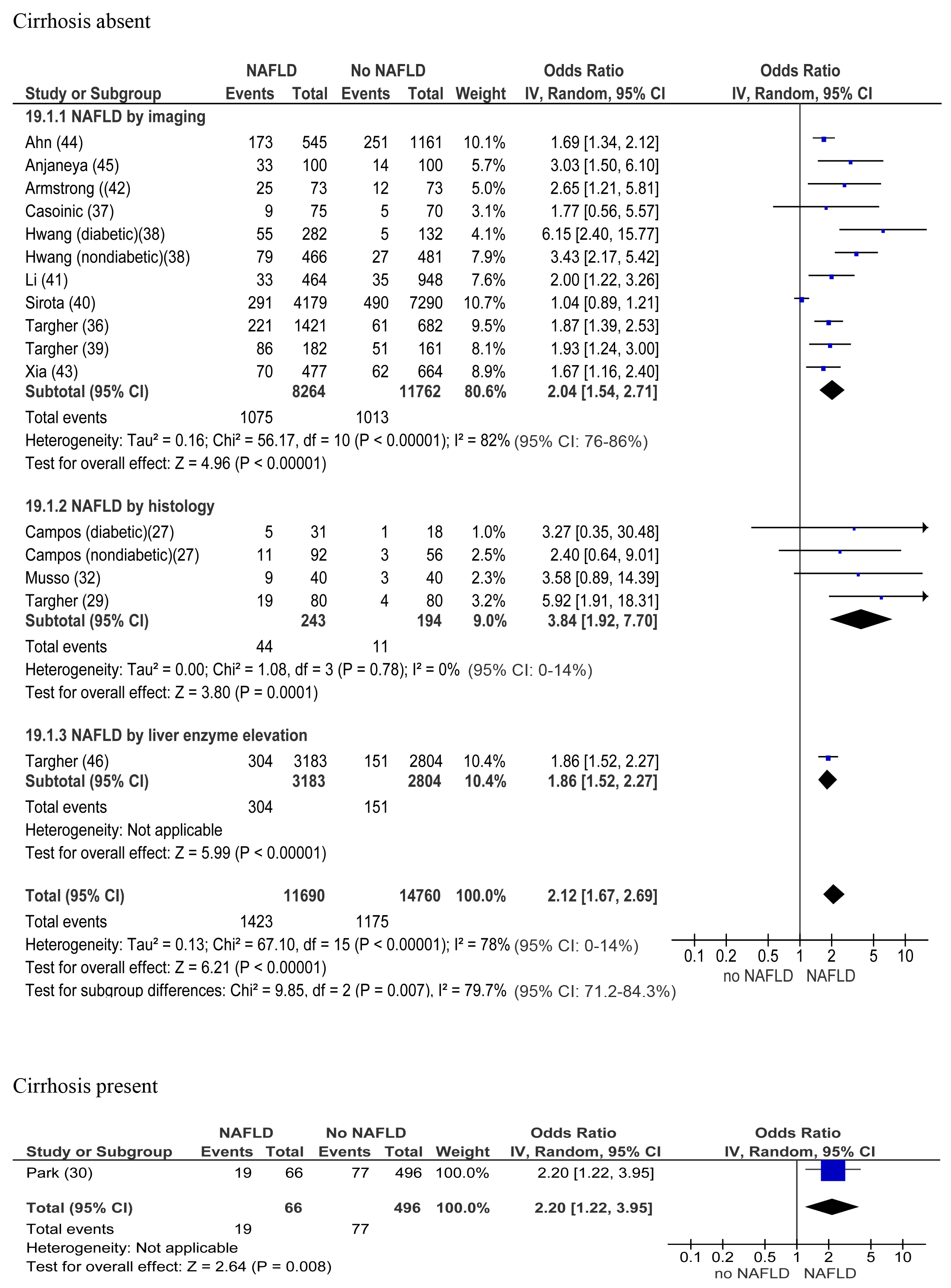


(95%CI:66-86%)

**Figure S20.** Forest plots of subgroup analyses for the outcome: prevalent chronic kidney disease (CKD) in cross-sectional studies. Equations used to estimate eGFR: MDRD versus CKD-EPI


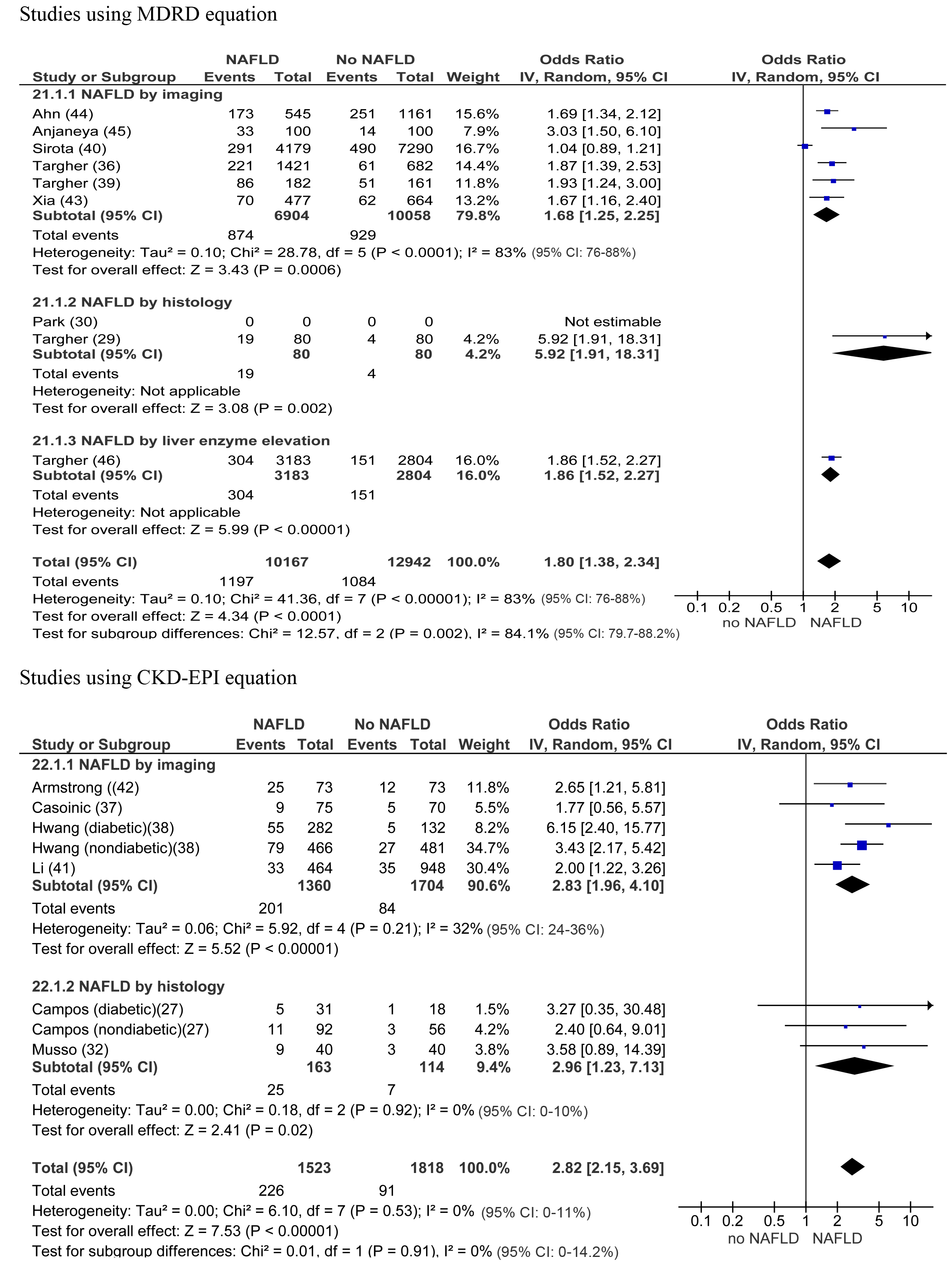


**Figure S21.** Forest plots of subgroup analyses for the outcome: prevalent chronic kidney disease (CKD) in cross-sectional studies. Outcomes related to CKD: both eGFR and proteinuria versus eGFR alone.


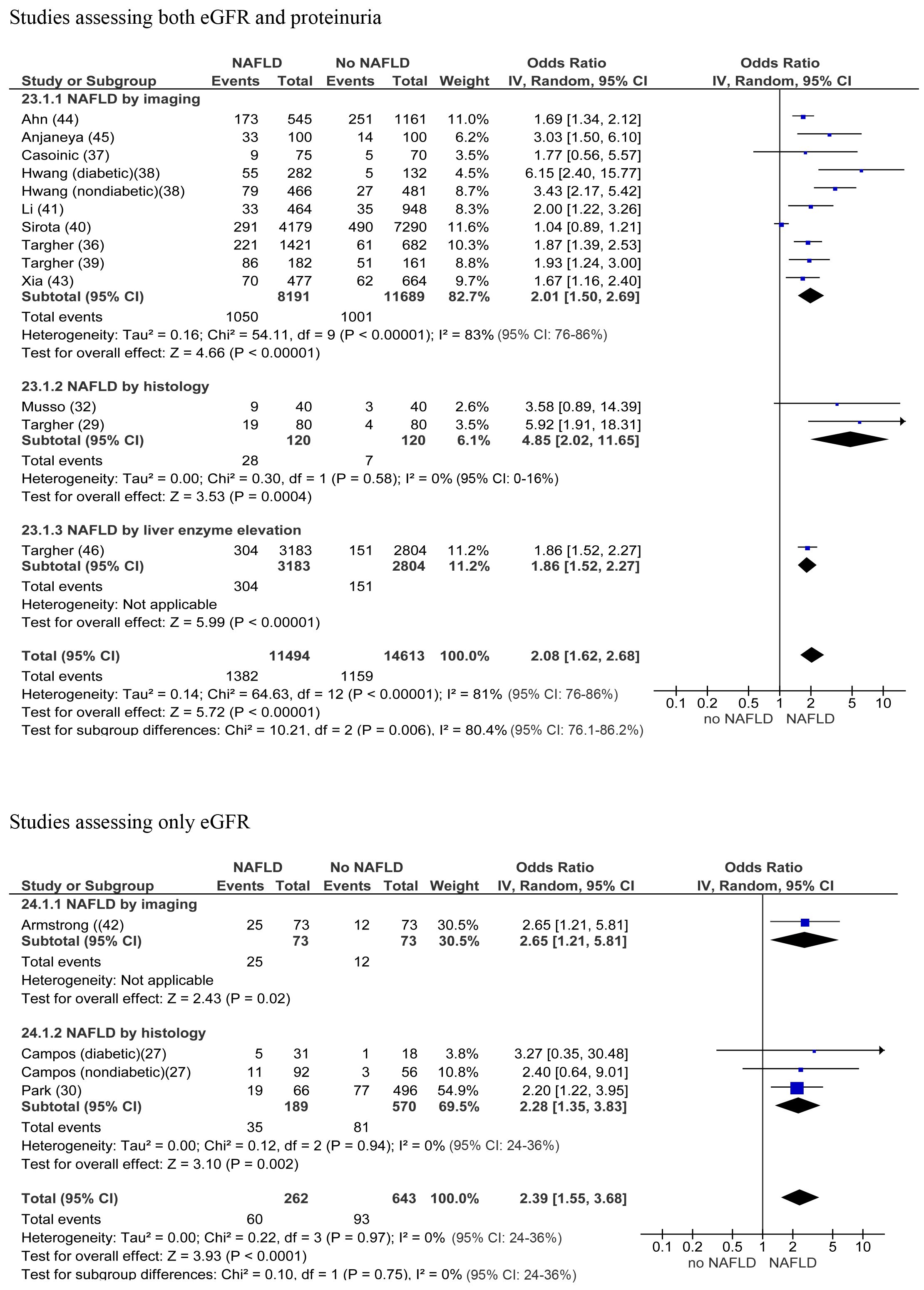


**Figure S22.** Forest plots of subgroup analyses for the outcome: prevalent chronic kidney disease (CKD) in cross-sectional studies. Data type: studies providing IPD vs. studies providing exclusively AD.


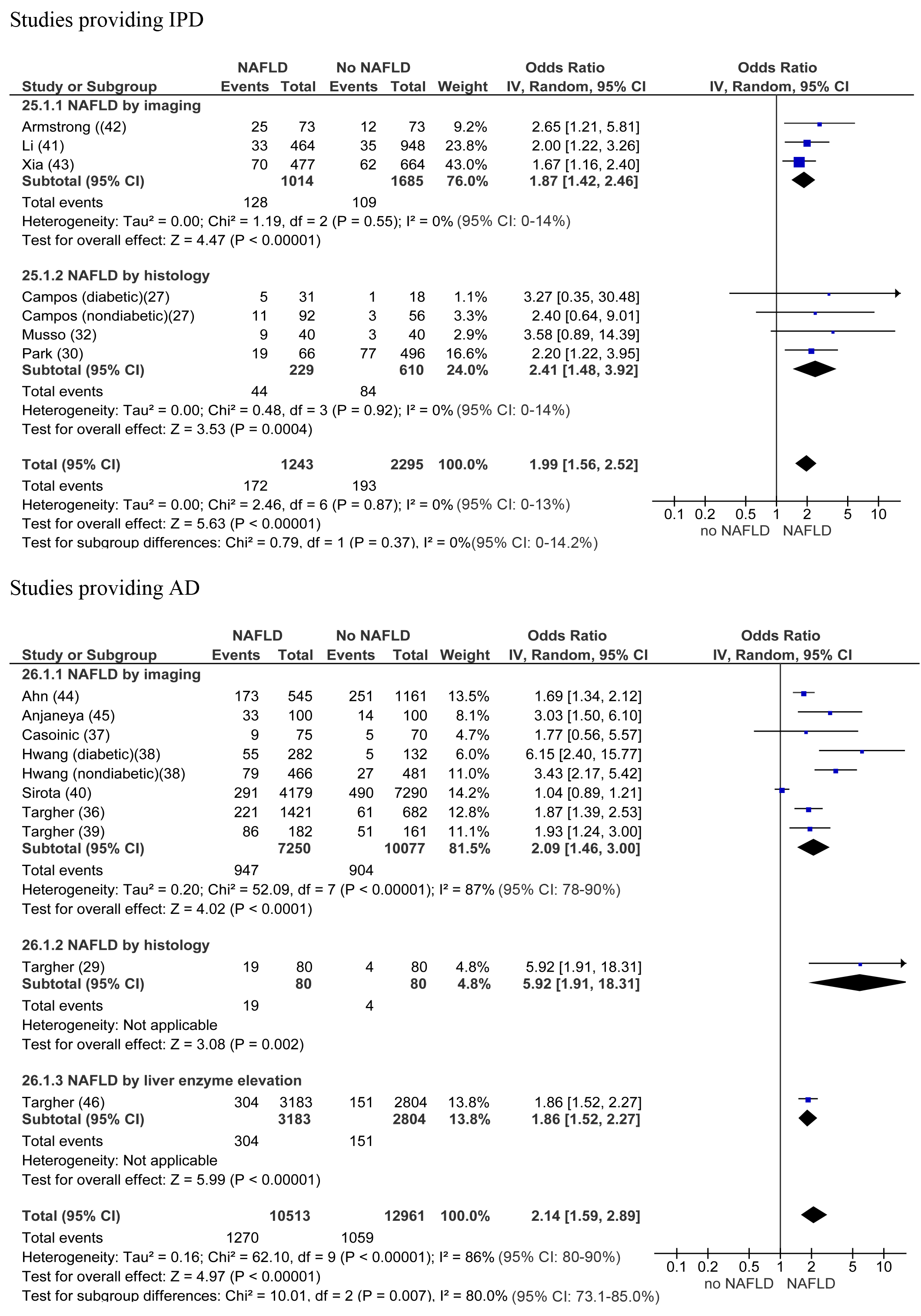


**Figure S23.** Forest plots of subgroup analyses for the outcome: incident chronic kidney disease (CKD) in

longitudinal studies. STROBE score item (p) fulfilment.


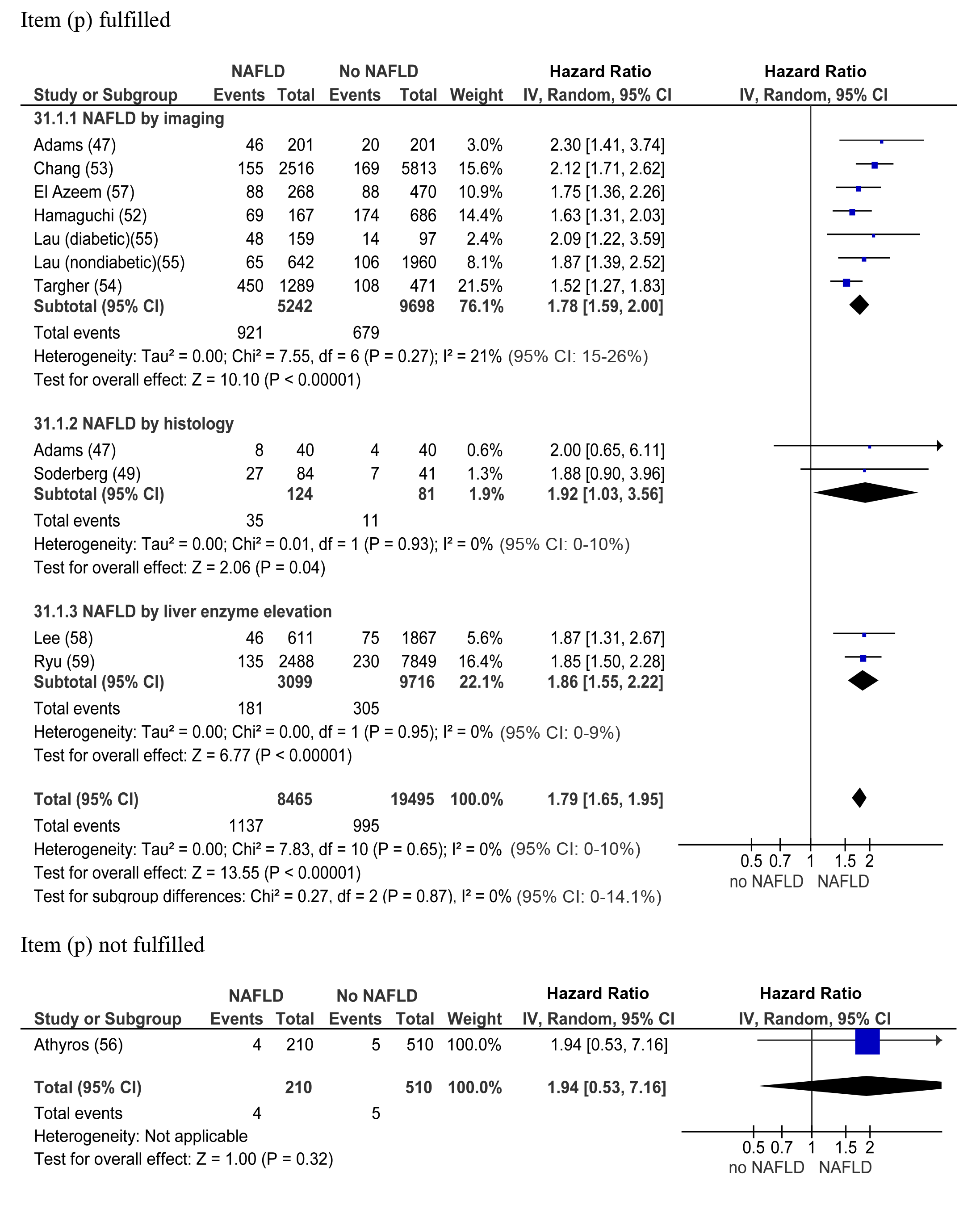


**Figure S24.** Forest plots of subgroup analyses for the outcome: incident chronic kidney disease (CKD) in

longitudinal studies. STROBE score item (s) fulfilment.


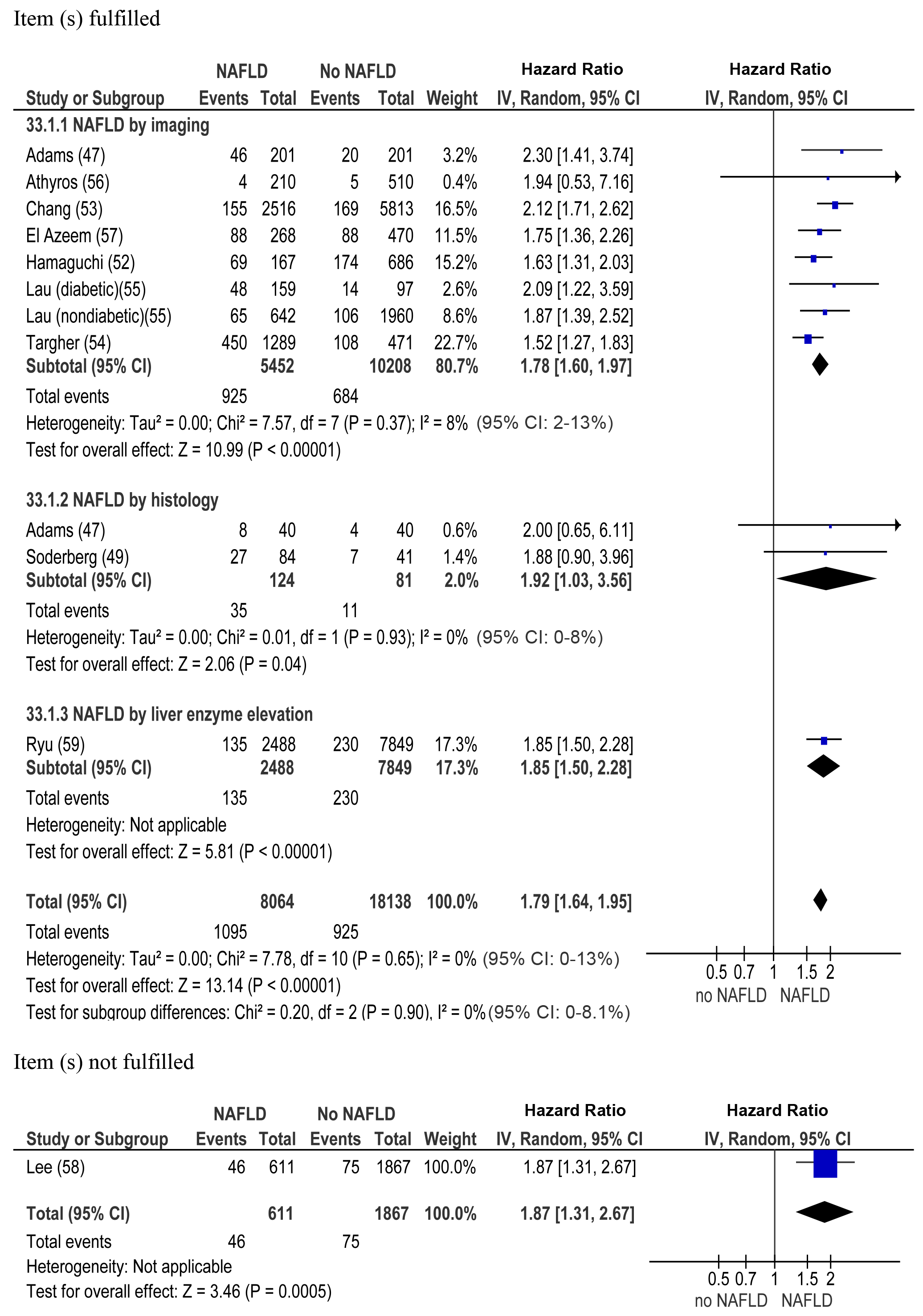


**Figure S25.** Forest plots of subgroup analyses for the outcome: incident chronic kidney disease (CKD) in

longitudinal studies. STROBE score item (t) fulfilment.


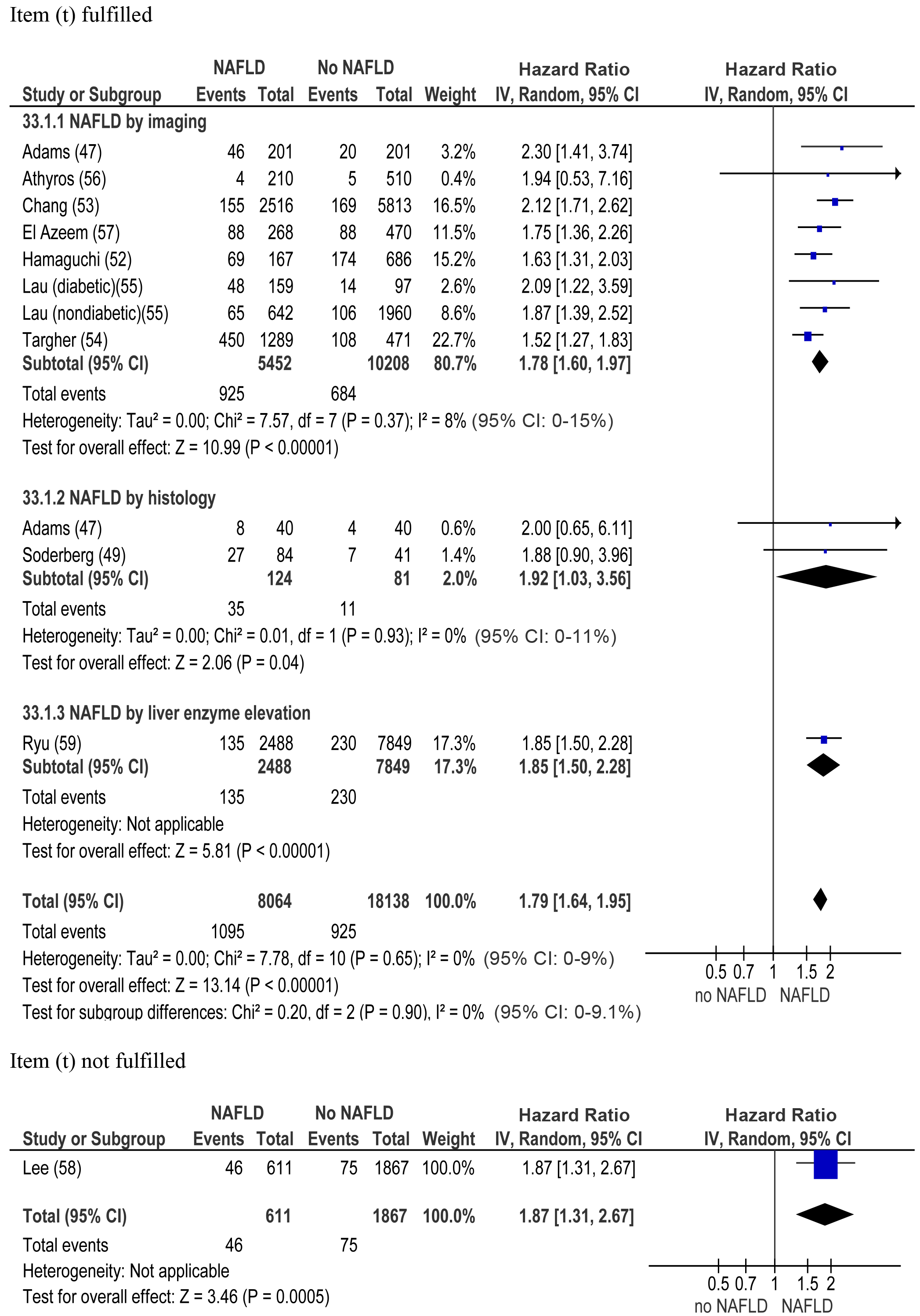


**Figure S26.** Forest plots of subgroup analyses for the outcome: incident chronic kidney disease (CKD) in

longitudinal studies. STROBE score item (v) fulfilment.


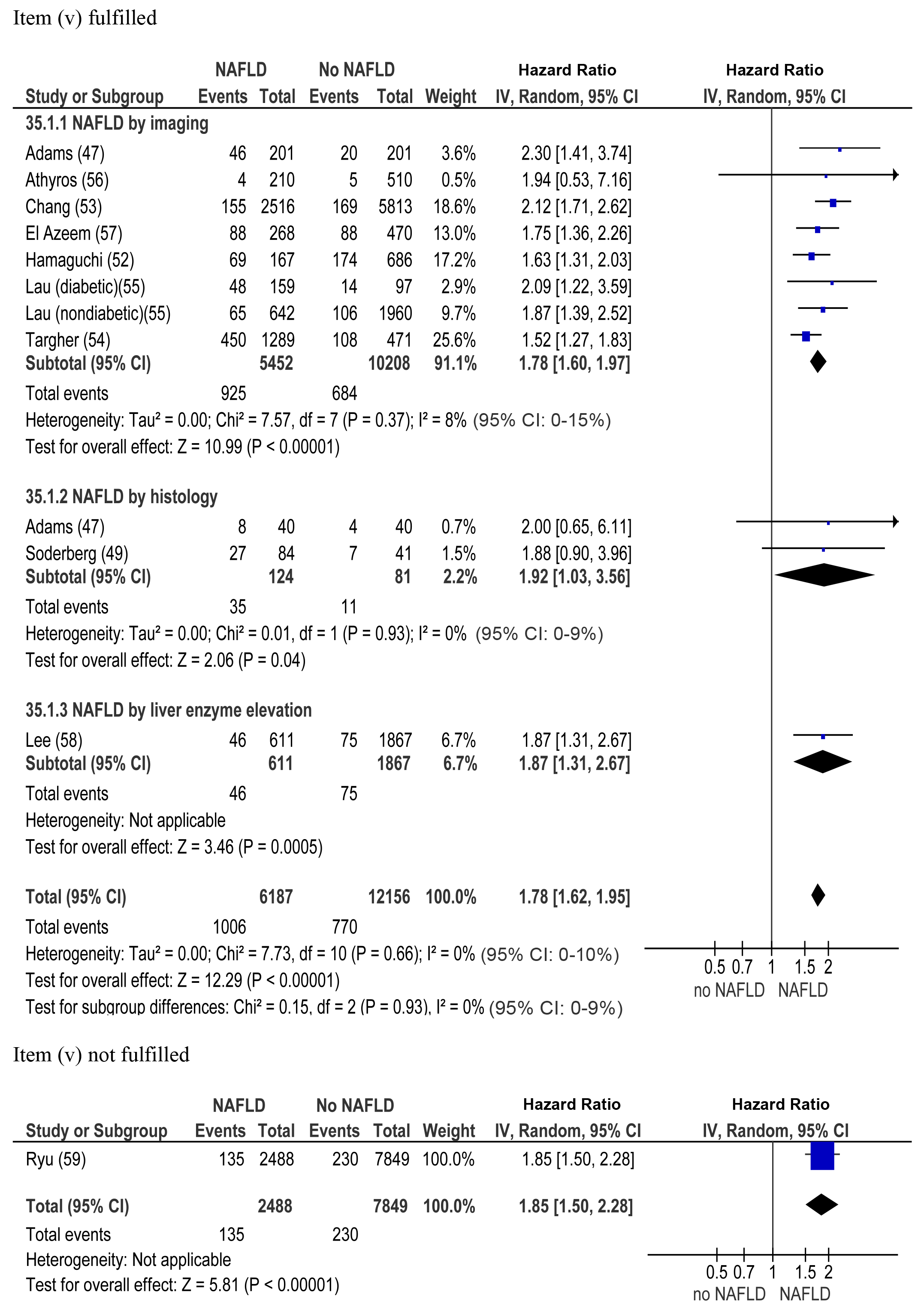


**Figure S27.** Forest plots of subgroup analyses for the outcome: incident chronic kidney disease (CKD) in

longitudinal studies. Presence of diabetes.


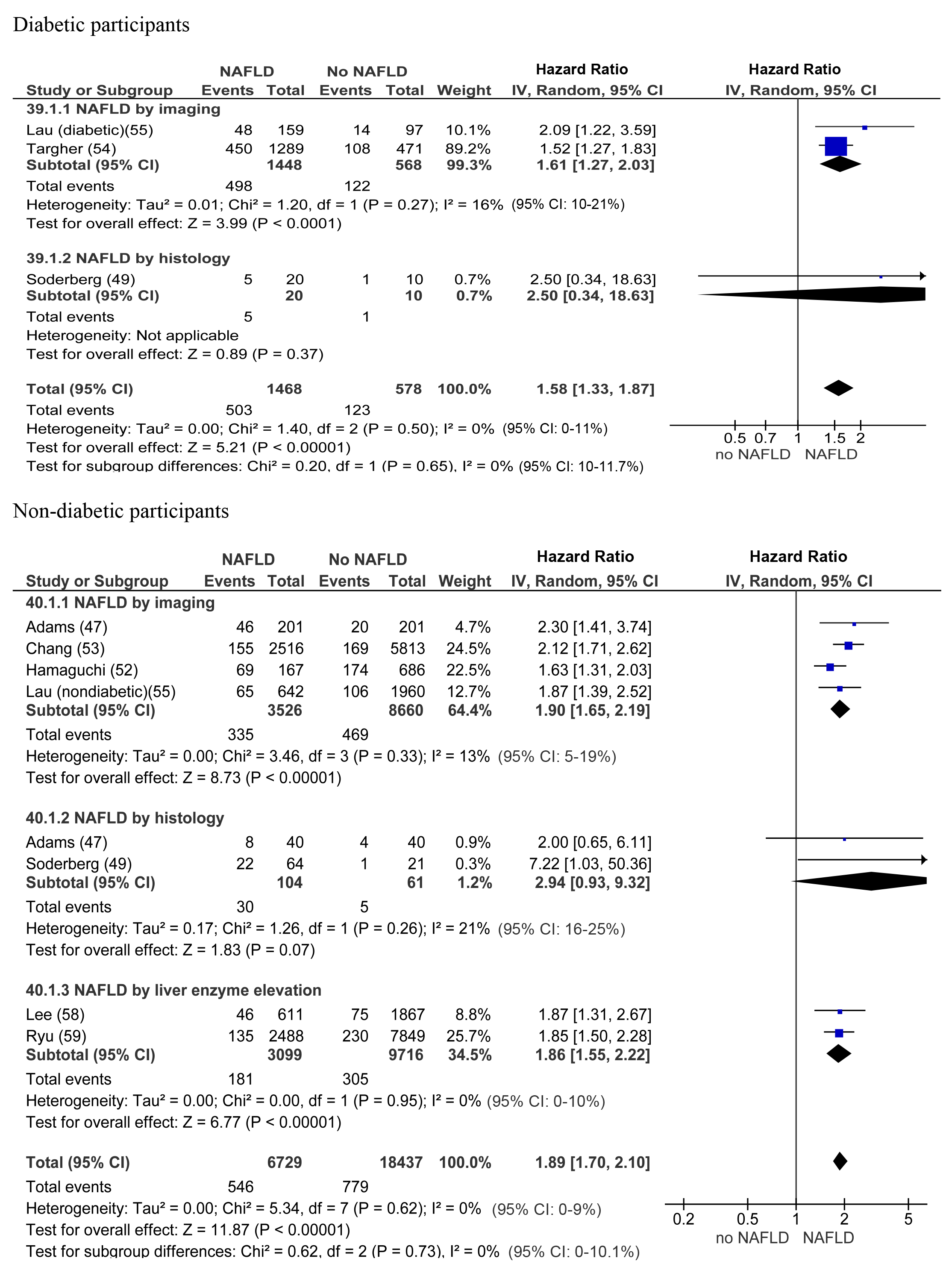


**Figure S28.** Forest plots of subgroup analyses for the outcome: incident chronic kidney disease (CKD) in

longitudinal studies. Adjustment for age and BMI and metabolic syndrome and hypertension and smoking status.


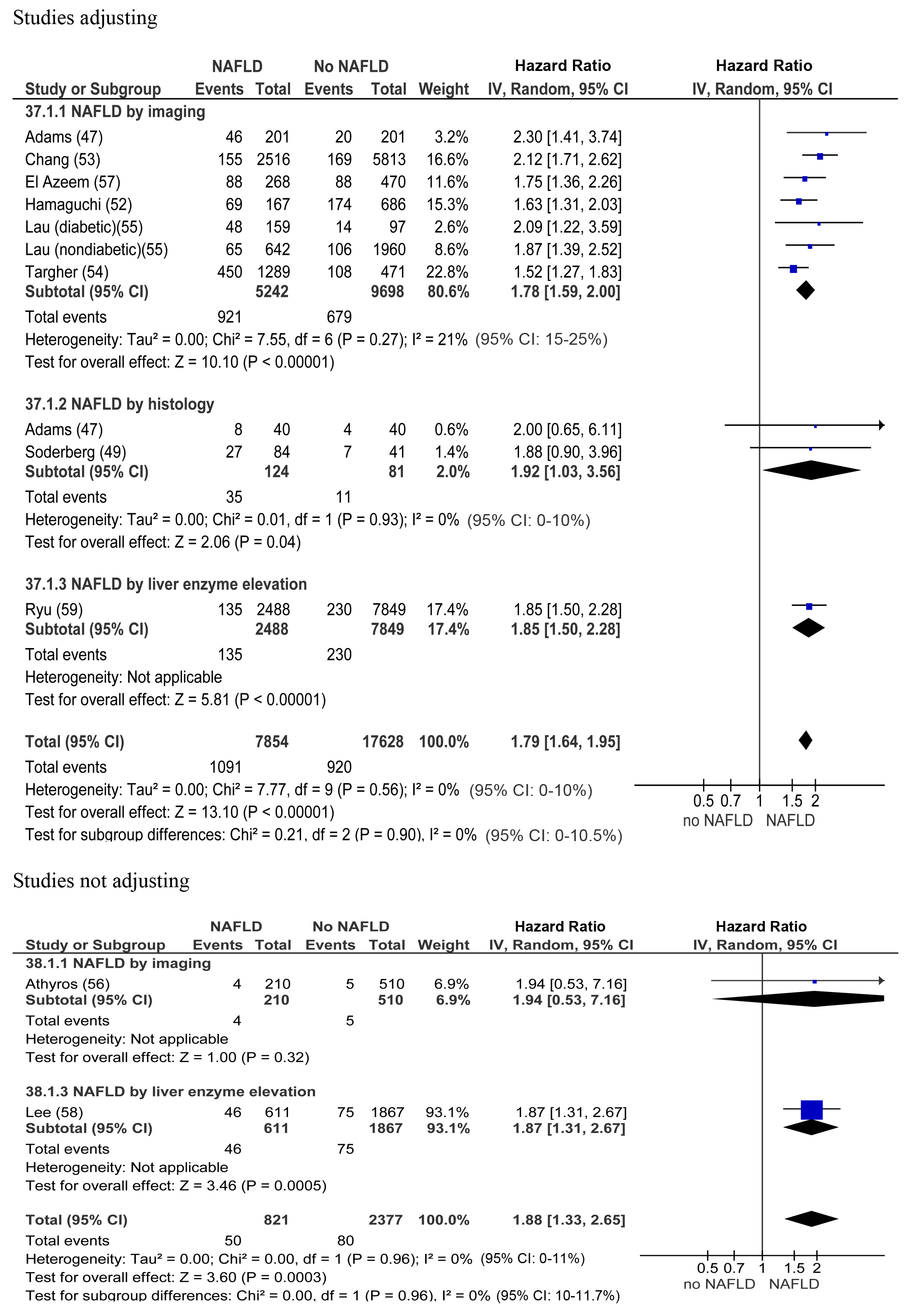


**Figure S29.** Forest plots of subgroup analyses for the outcome: incident chronic kidney disease (CKD) in

longitudinal studies. Study design: population-based vs. hospital-based)
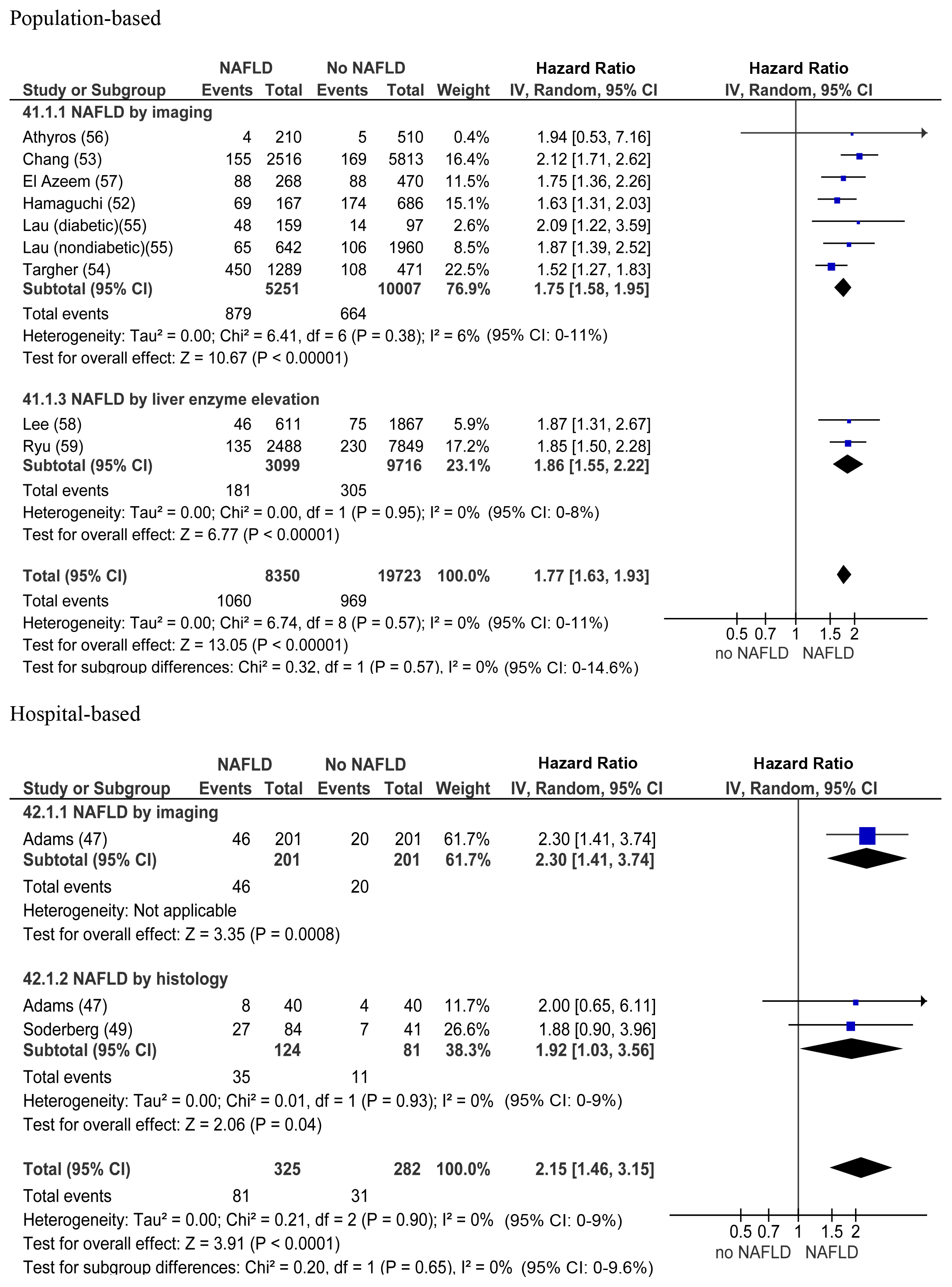


**Figure S30.** Forest plots of subgroup analyses for the outcome: incident chronic kidney disease (CKD) in

longitudinal studies. Ethnicity: Asian vs. non-Asian participants


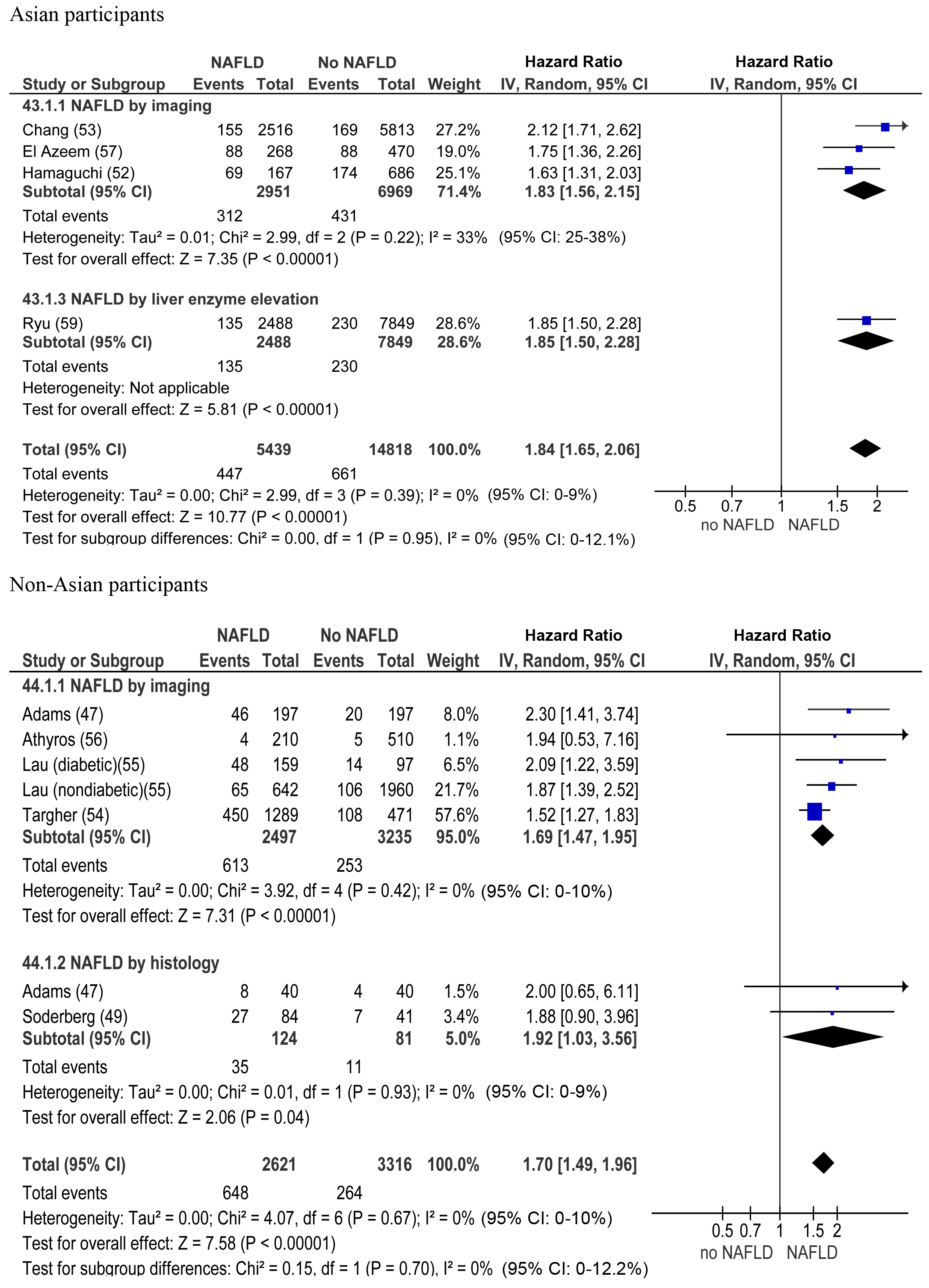


**Figure S31.** Forest plots of subgroup analyses for the outcome: incident chronic kidney disease (CKD) in

longitudinal studies. Equation used to estimate eGFR: MDRD versus CKD-EPI.


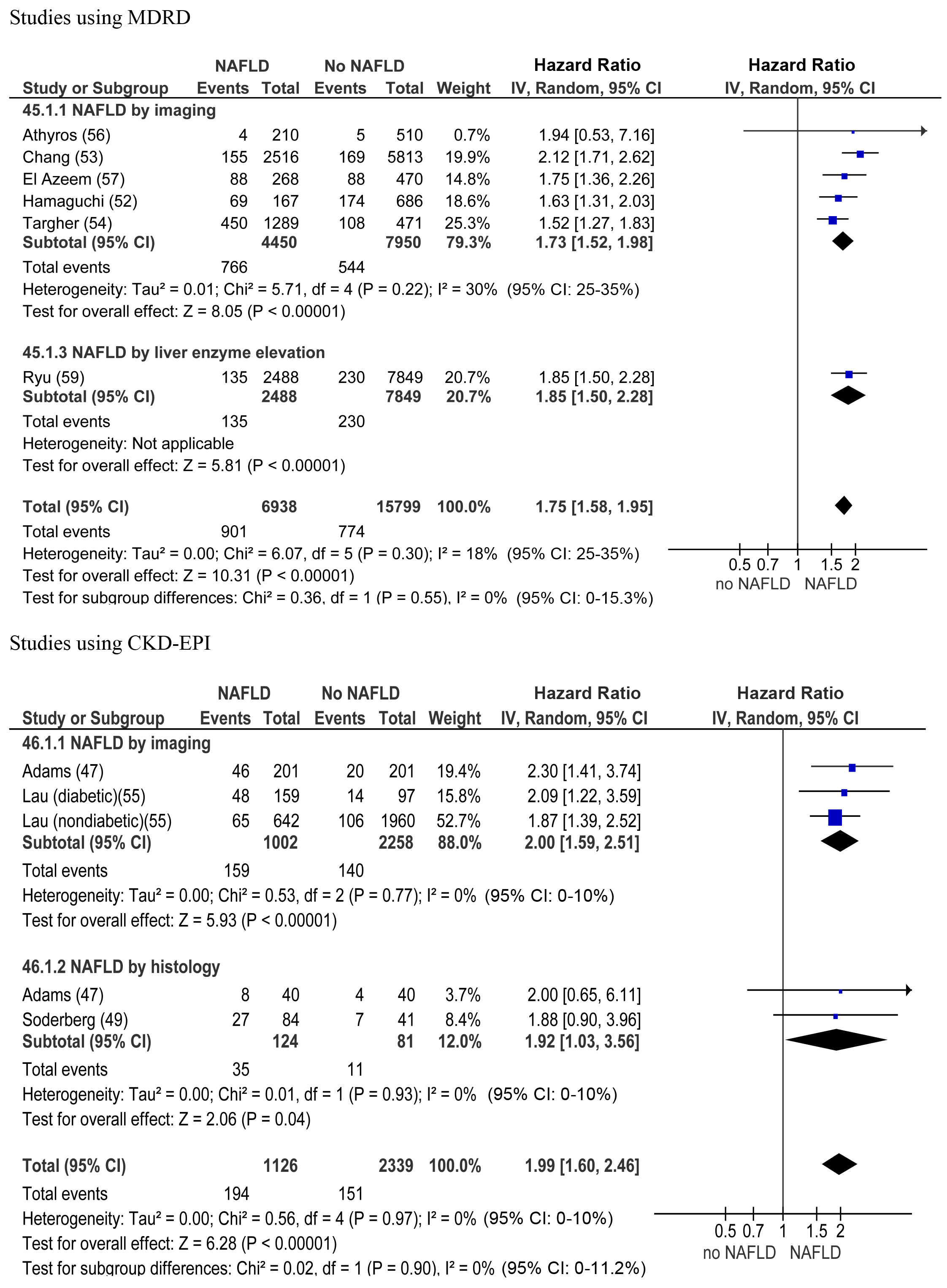


**Figure S32.** Forest plots of subgroup analyses for the outcome: incident chronic kidney disease (CKD) in

longitudinal studies. Outcomes related to CKD: both eGFR and proteinuria vs. eGFR alone vs. proteinuria alone.


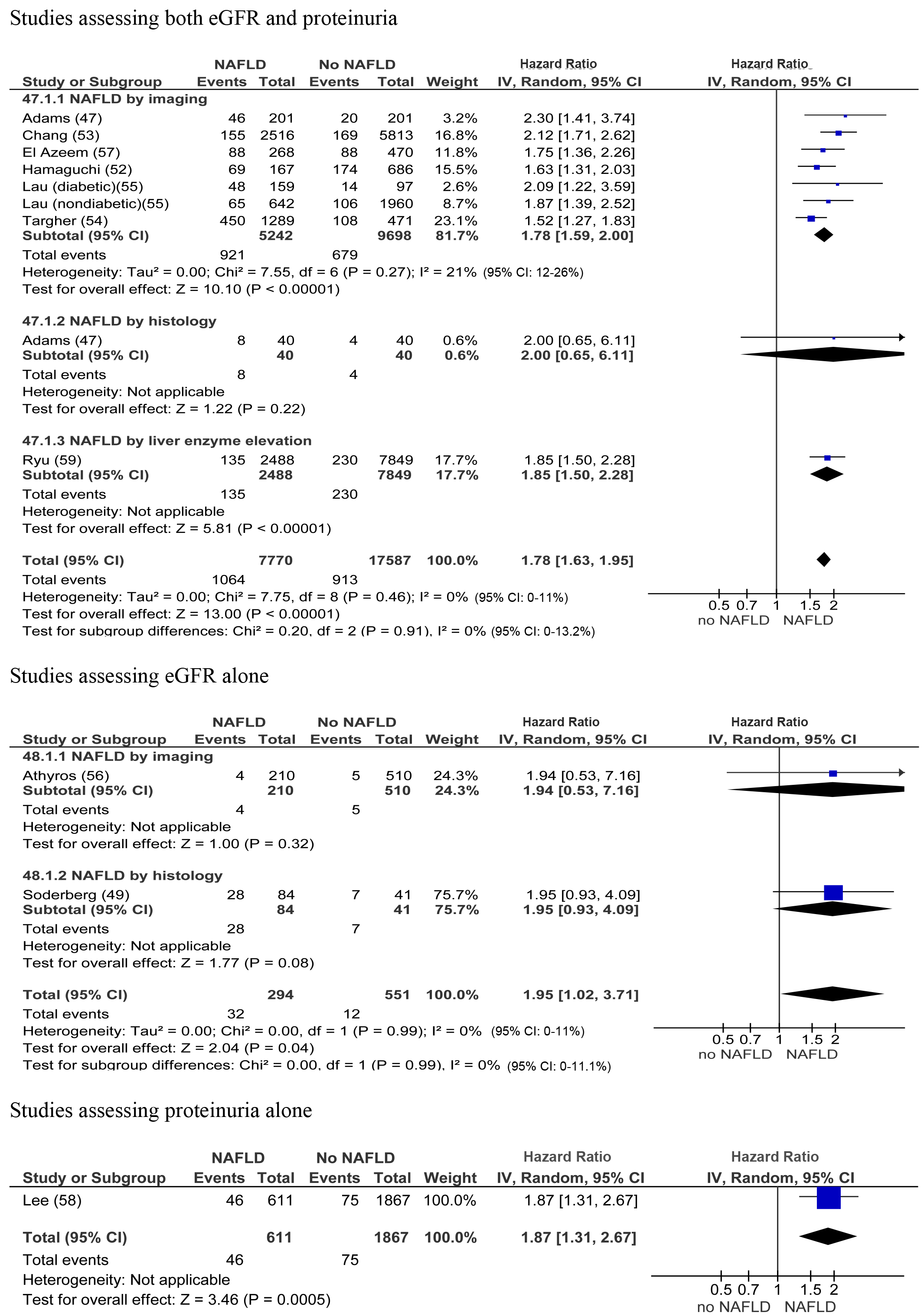


**Figure S33.** Forest plots of subgroup analyses for the outcome: incident chronic kidney disease (CKD) in

longitudinal studies. Data type: studies providing IPD versus studies providing exclusively AD.


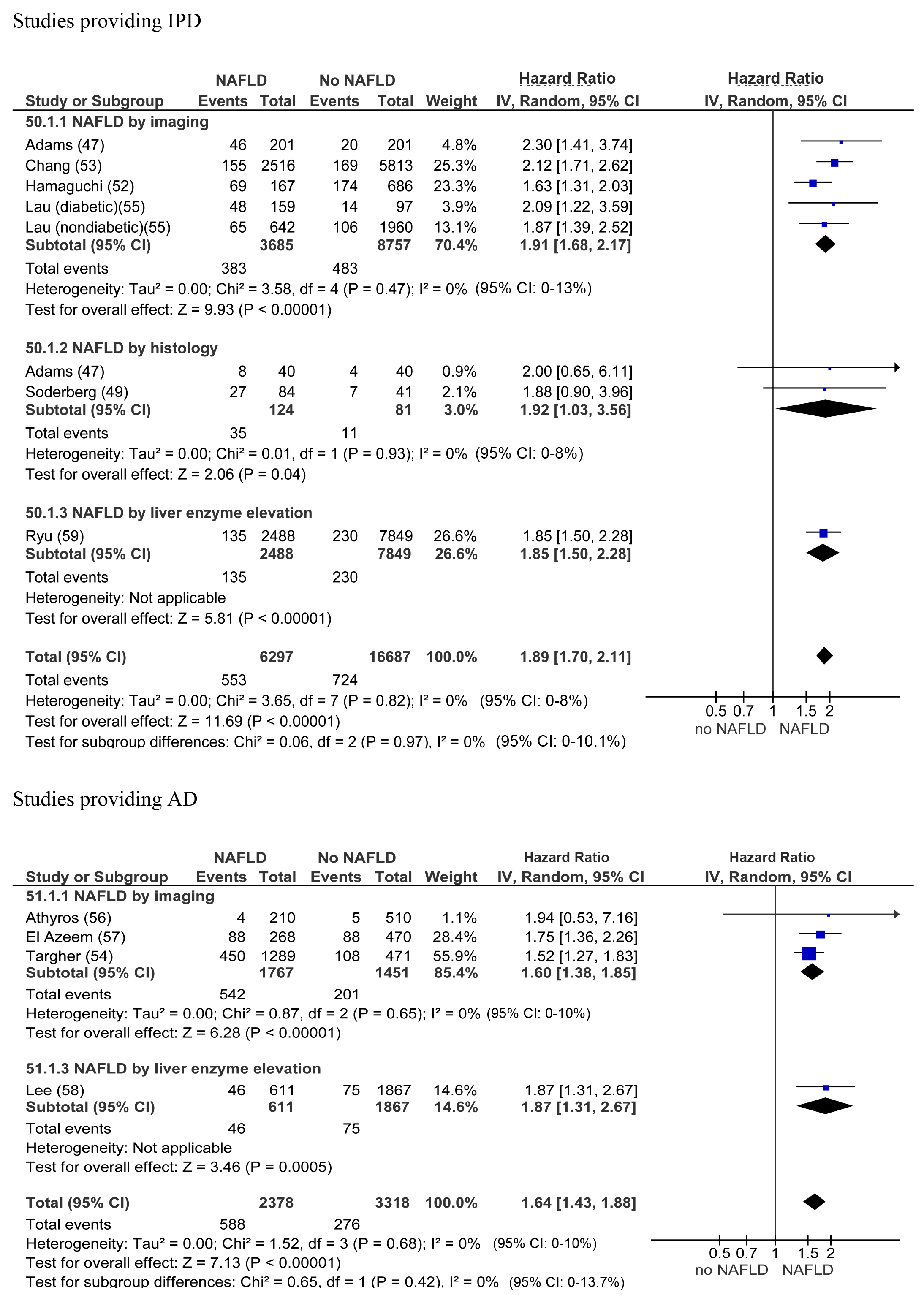


**Figure S34.** Forest plots of subgroup analyses for the outcome: prevalent chronic kidney disease (CKD) in NASH vs. simple steatosis in cross-sectional studies. STROBE score item (v) fulfilment.


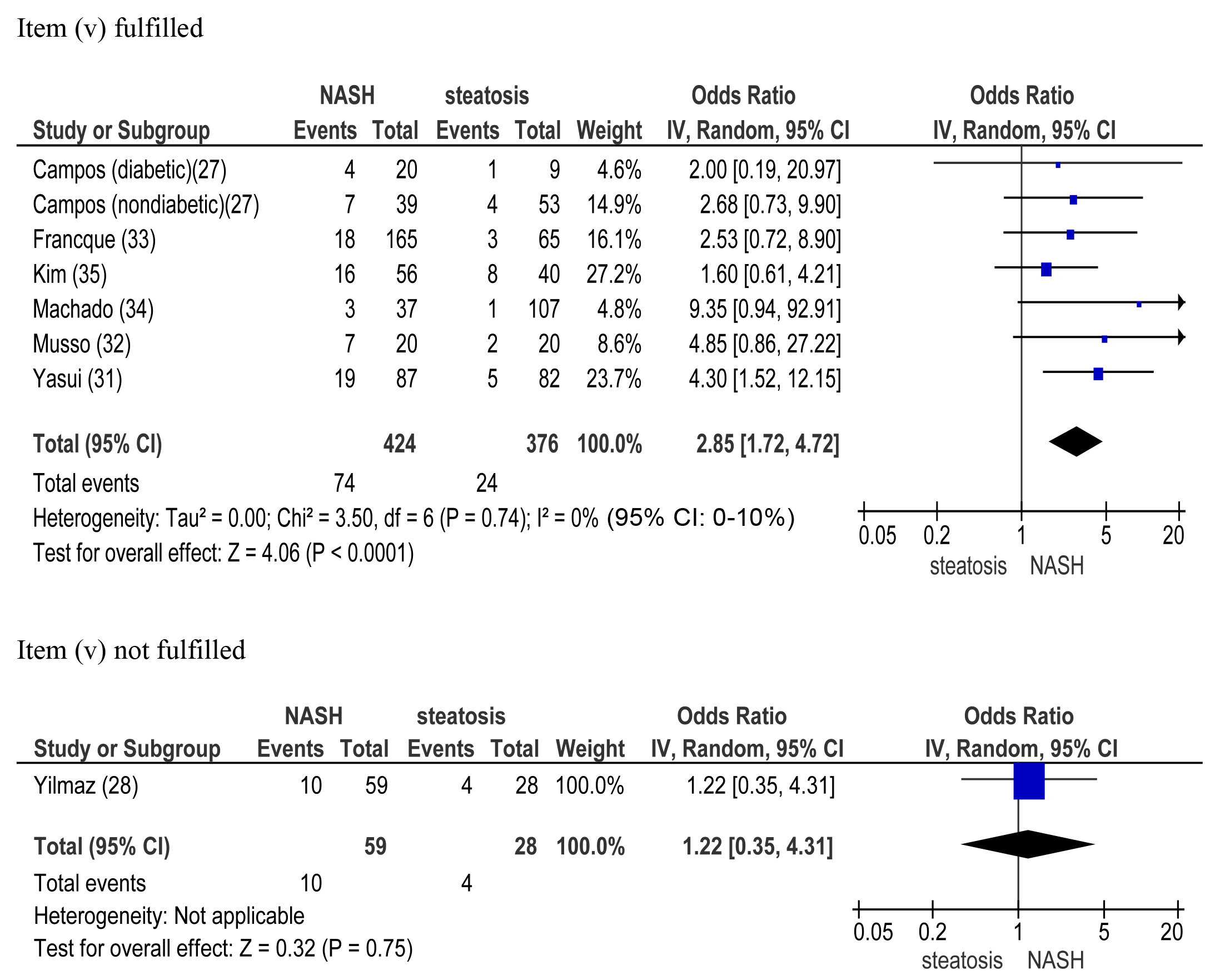


**Figure S35.** Forest plots of subgroup analyses for the outcome: prevalent chronic kidney disease (CKD) in NASH vs. simple steatosis in cross-sectional studies. Presence of diabetes.


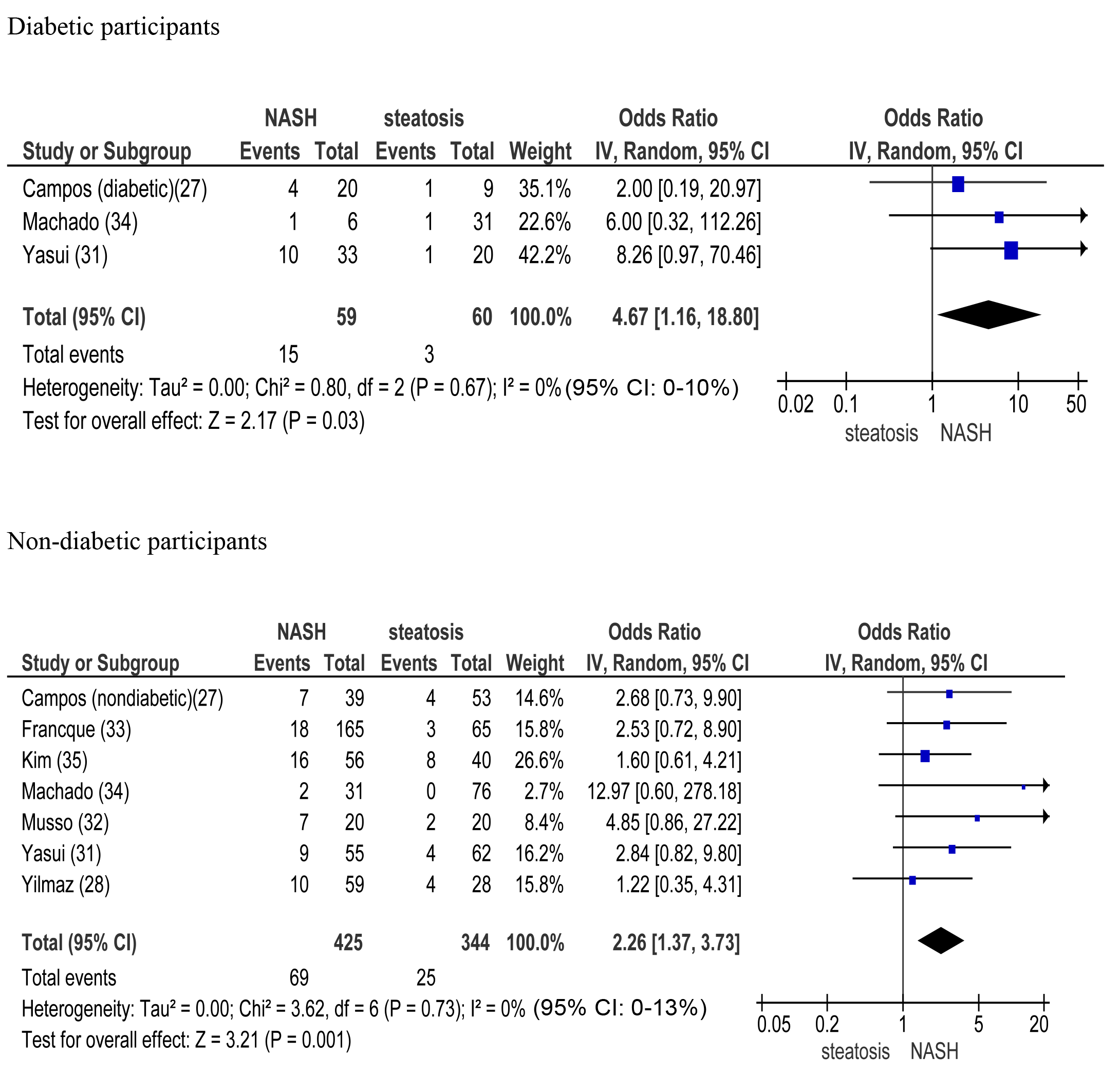


**Figure S36.** Forest plots of subgroup analyses for the outcome: prevalent chronic kidney disease (CKD) in NASH vs. simple steatosis in cross-sectional studies. Ethnicity: Asian vs. non-Asian participants.


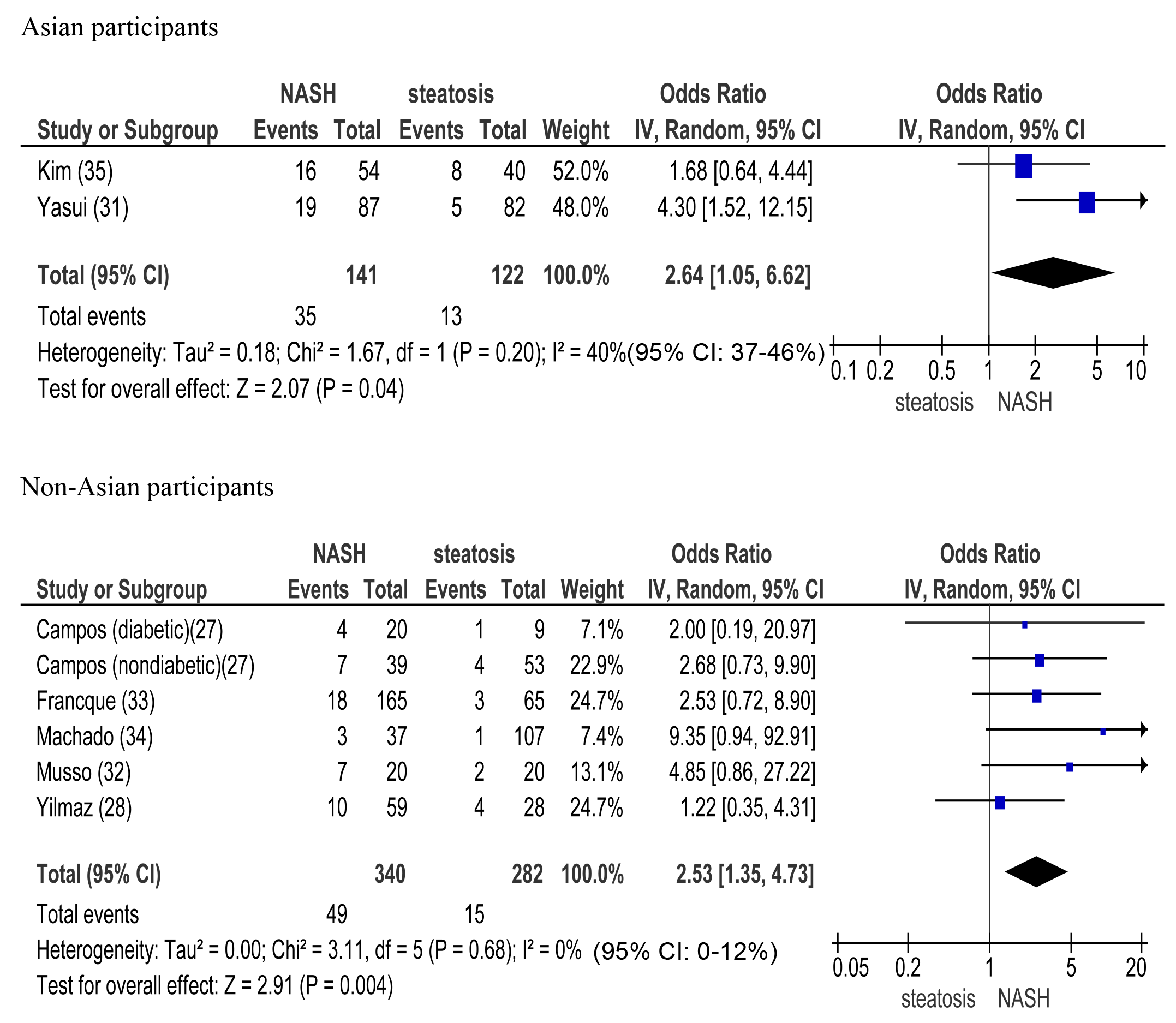


**Figure S37.** Forest plots of subgroup analyses for the outcome: prevalent chronic kidney disease (CKD) in NASH vs. simple steatosis in cross-sectional studies. Outcomes related to CKD: both eGFR and proteinuria vs. eGFR alone.


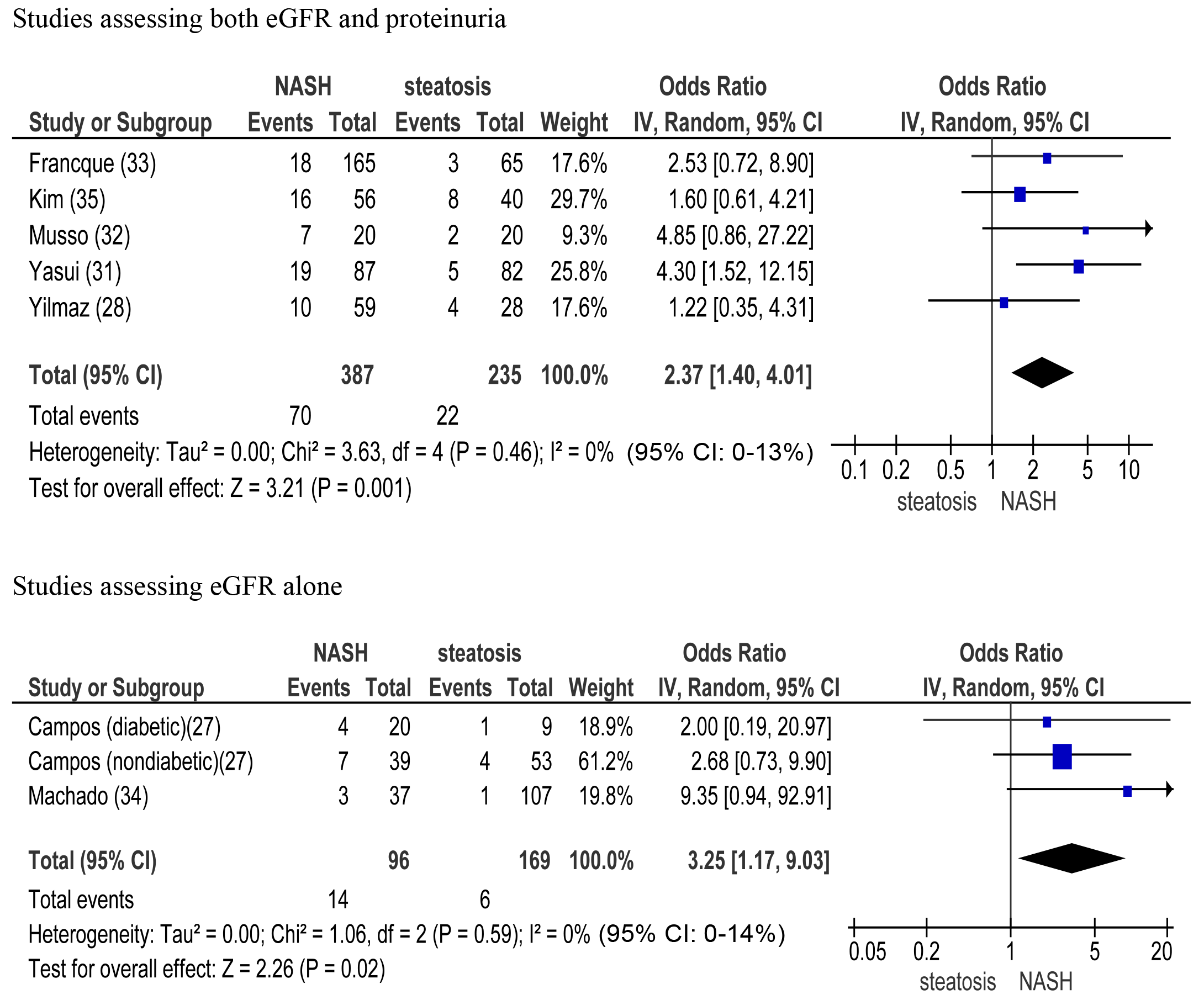


**Figure S38.** Forest plots of subgroup analyses for the outcome: prevalent chronic kidney disease (CKD) in advanced (stage F3) vs. non-advanced (stage F0-2) fibrosis in cross-sectional studies. STROBE score item (v) fulfilment.


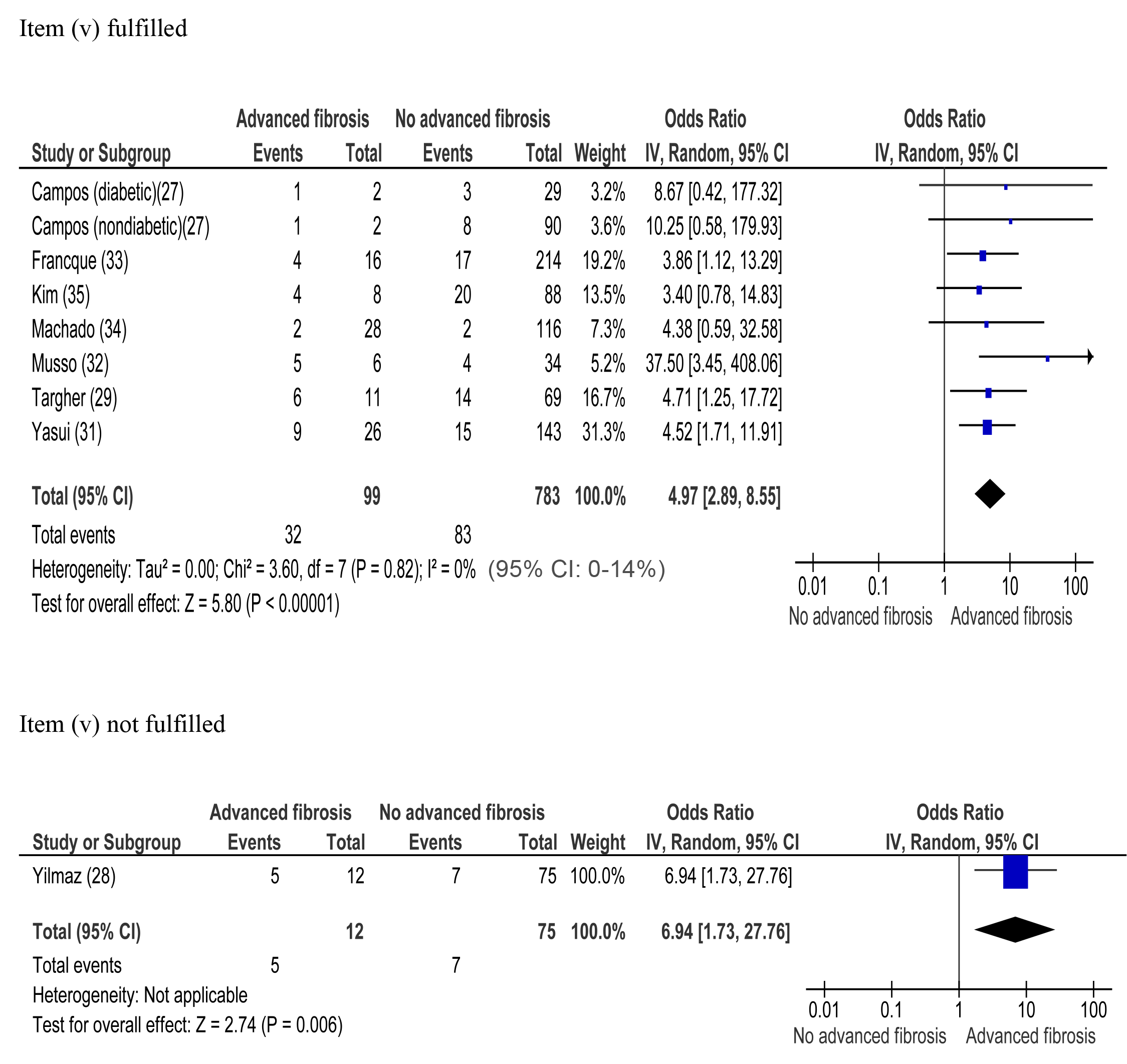


**Figure S39.** Forest plots of subgroup analyses for the outcome: prevalent chronic kidney disease (CKD) in advanced (stage F3) vs. non-advanced (stage F0-2) fibrosis in cross-sectional studies. Presence of diabetes.


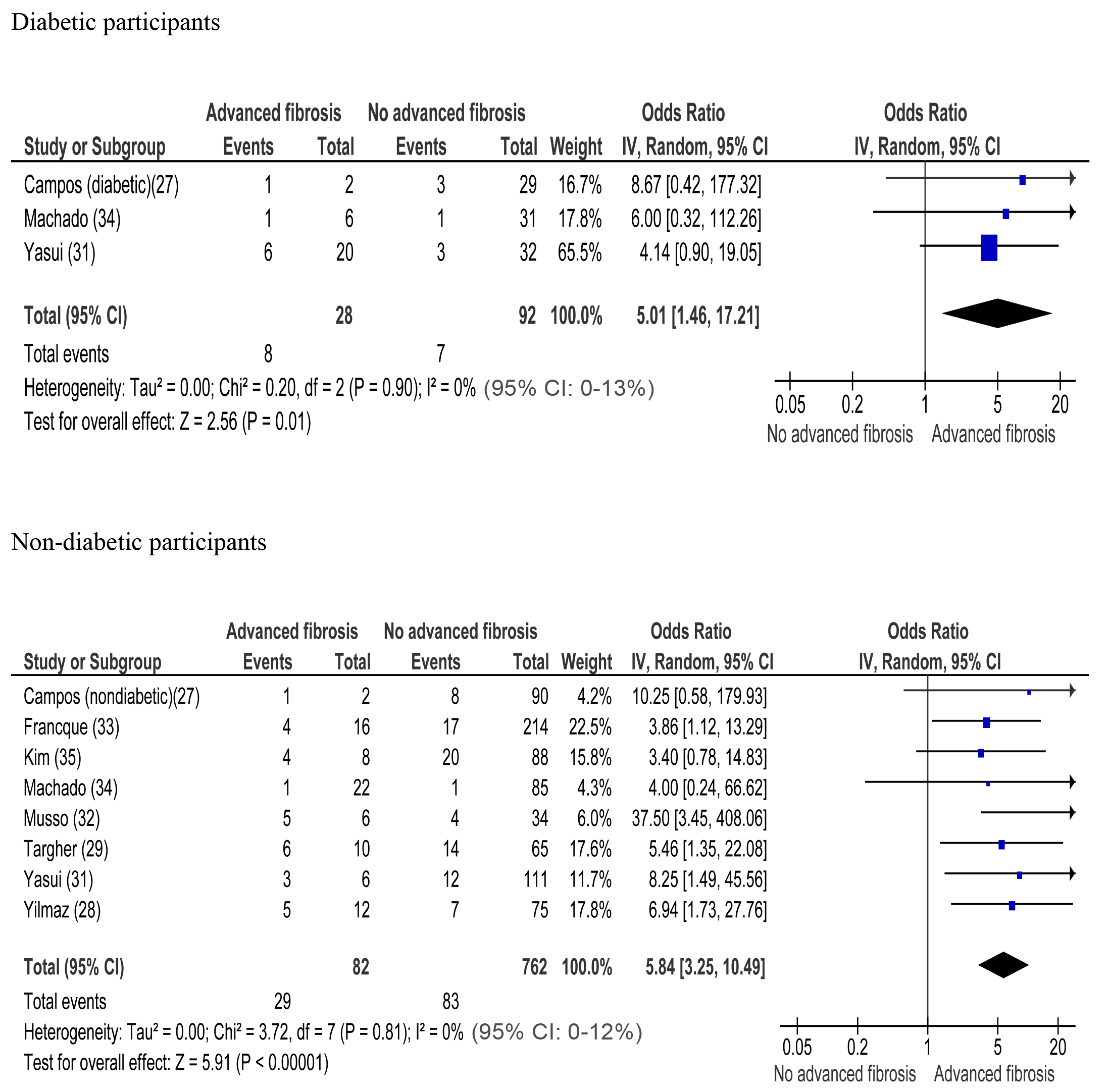


**Figure S40.** Forest plots of subgroup analyses for the outcome: prevalent chronic kidney disease (CKD) in advanced (stage F3) vs. non-advanced (stage F0-2) fibrosis in cross-sectional studies. Ethnicity: Asian vs. non-Asian.


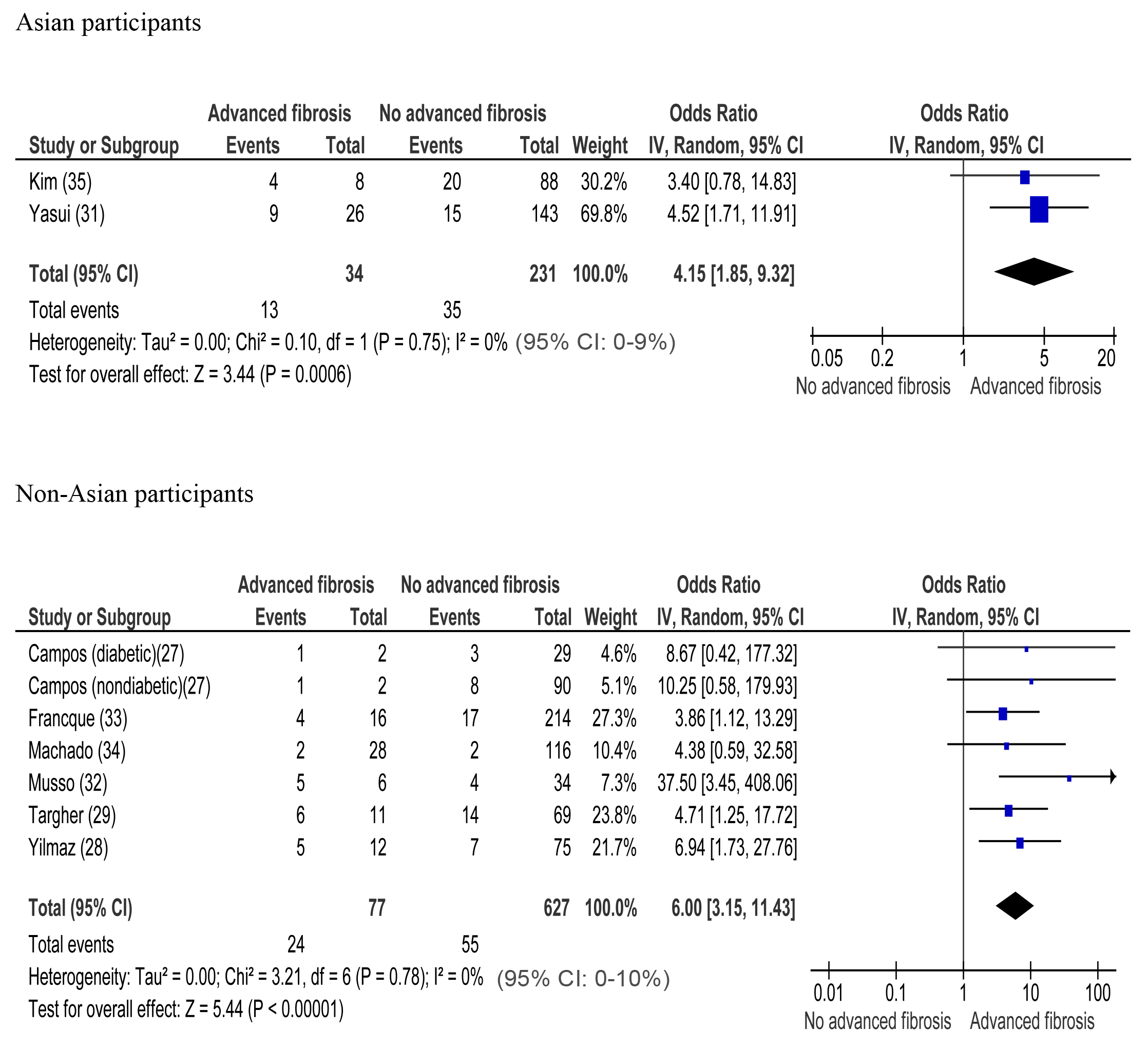


**Figure S41.** Forest plots of subgroup analyses for the outcome: prevalent chronic kidney disease (CKD) in advanced (stage F3) vs. non-advanced (stage F0-2) fibrosis in cross-sectional studies. Equation used to estimate eGFR: MDRD versus CKD-EPI.


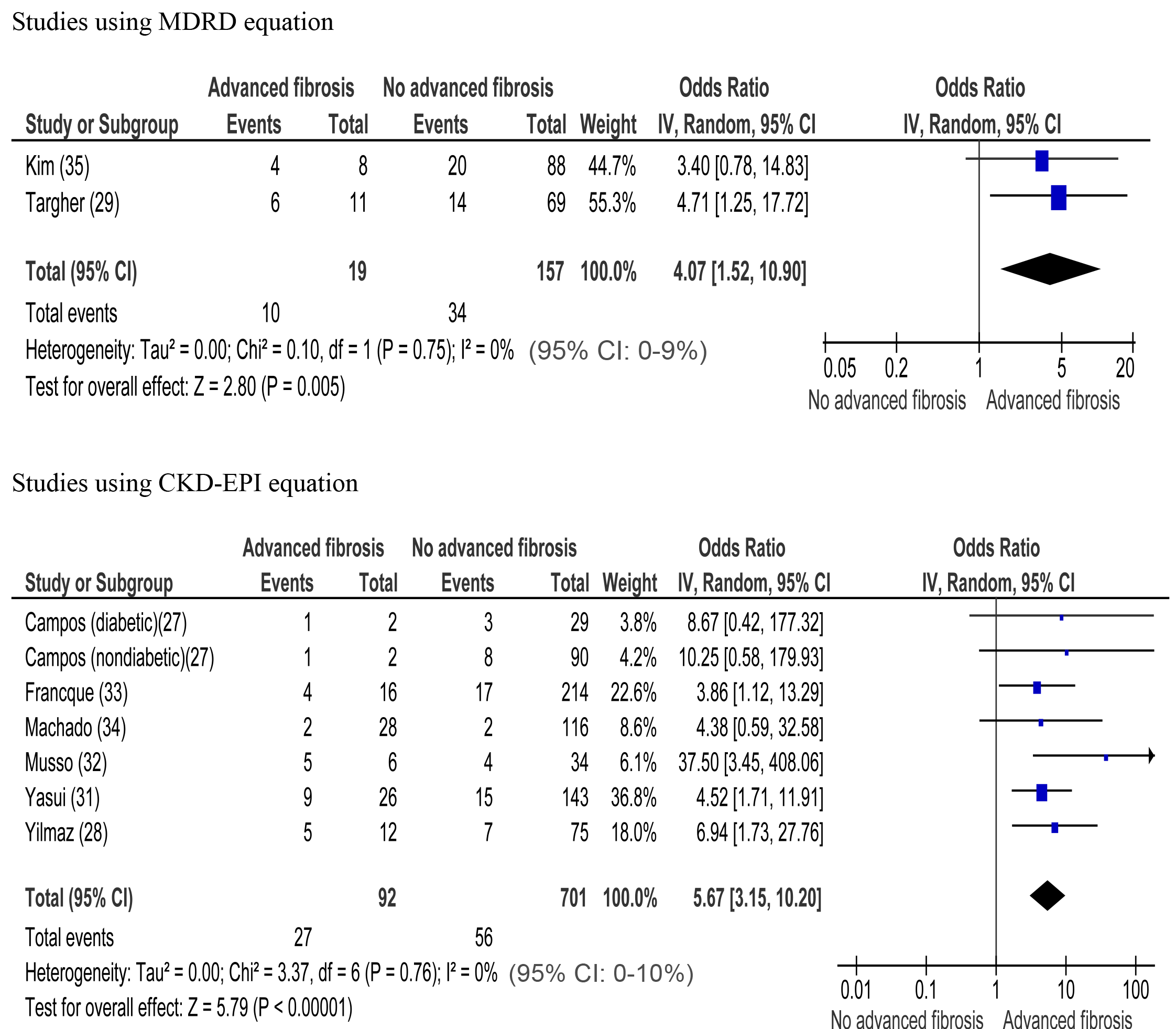


**Figure S42.** Forest plots of subgroup analyses for the outcome: prevalent chronic kidney disease (CKD) in advanced (stage F3) vs. non-advanced (stage F0-2) fibrosis in cross-sectional studies. Outcomes related to CKD: both eGFR and proteinuria versus eGFR alone.

Studies assessing both eGFR and proteinuria


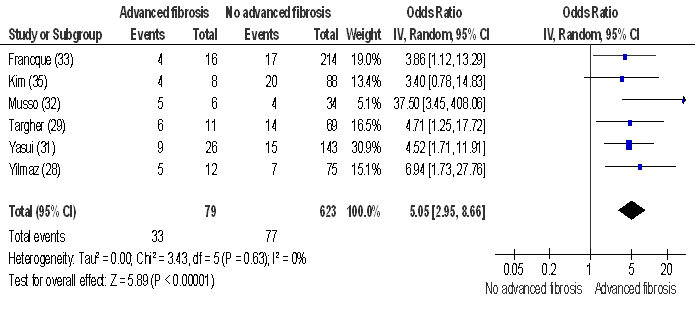


(95% CI: 0-9%)

Studies assessing only eGFR


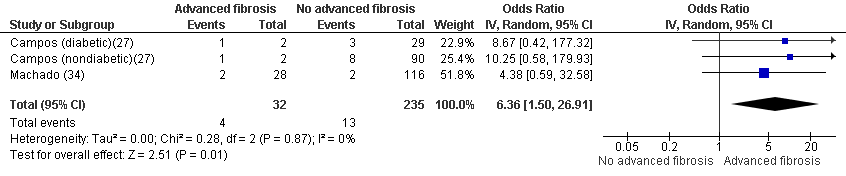


(95% CI: 0-10%)

**Figure S43.** Forest plots of subgroup analyses for the outcome: prevalent chronic kidney disease (CKD) in advanced (stage F3) vs. non-advanced (stage F0-2) fibrosis in cross-sectional studies. Data type: studies providing IPD vs. studies providing exclusively AD.


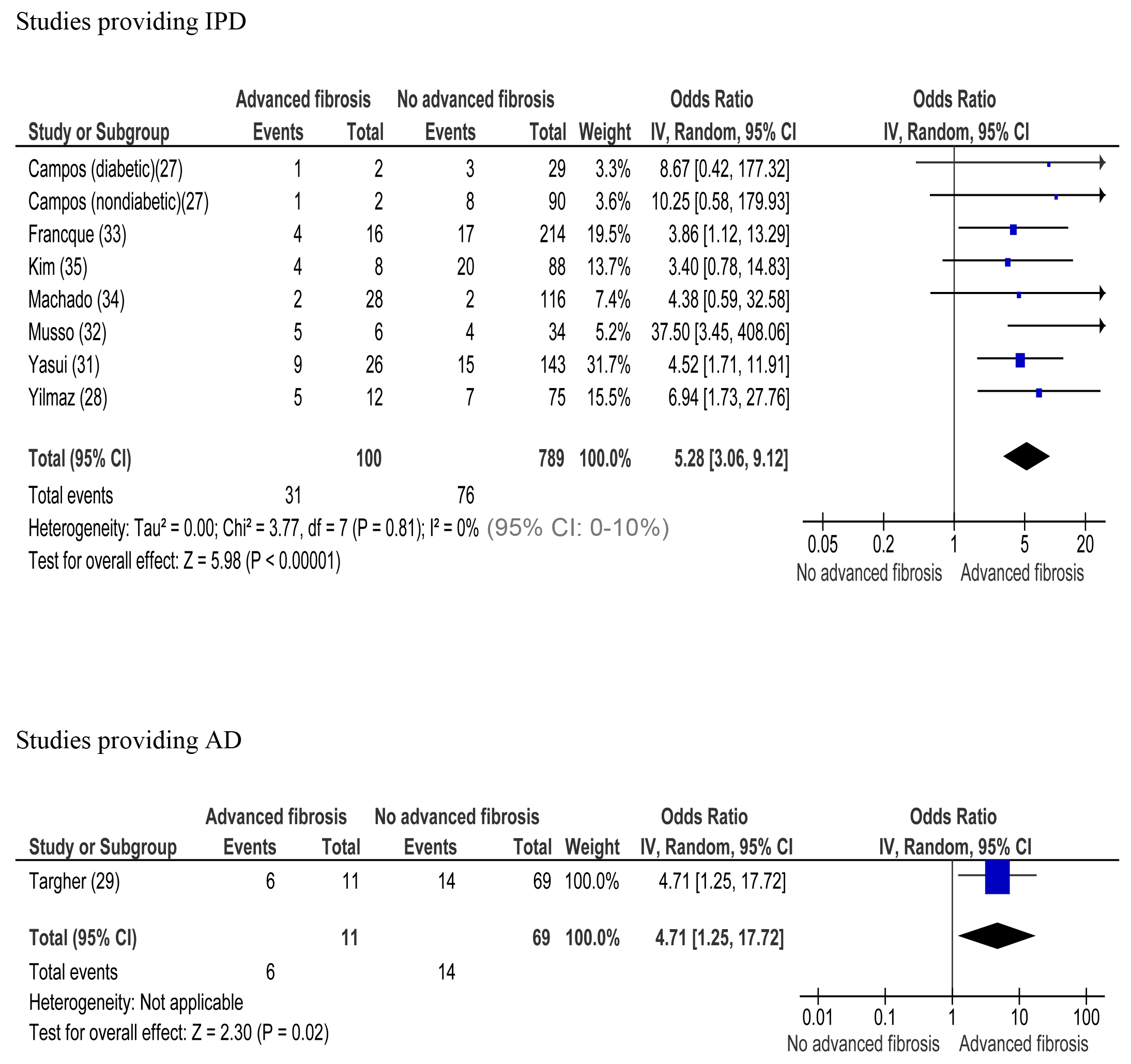


**Figure S44.** Forest plots of subgroup analyses for the outcome: incident chronic kidney disease (CKD) in

NASH vs. simple steatosis in prospective studies. Presence of diabetes.


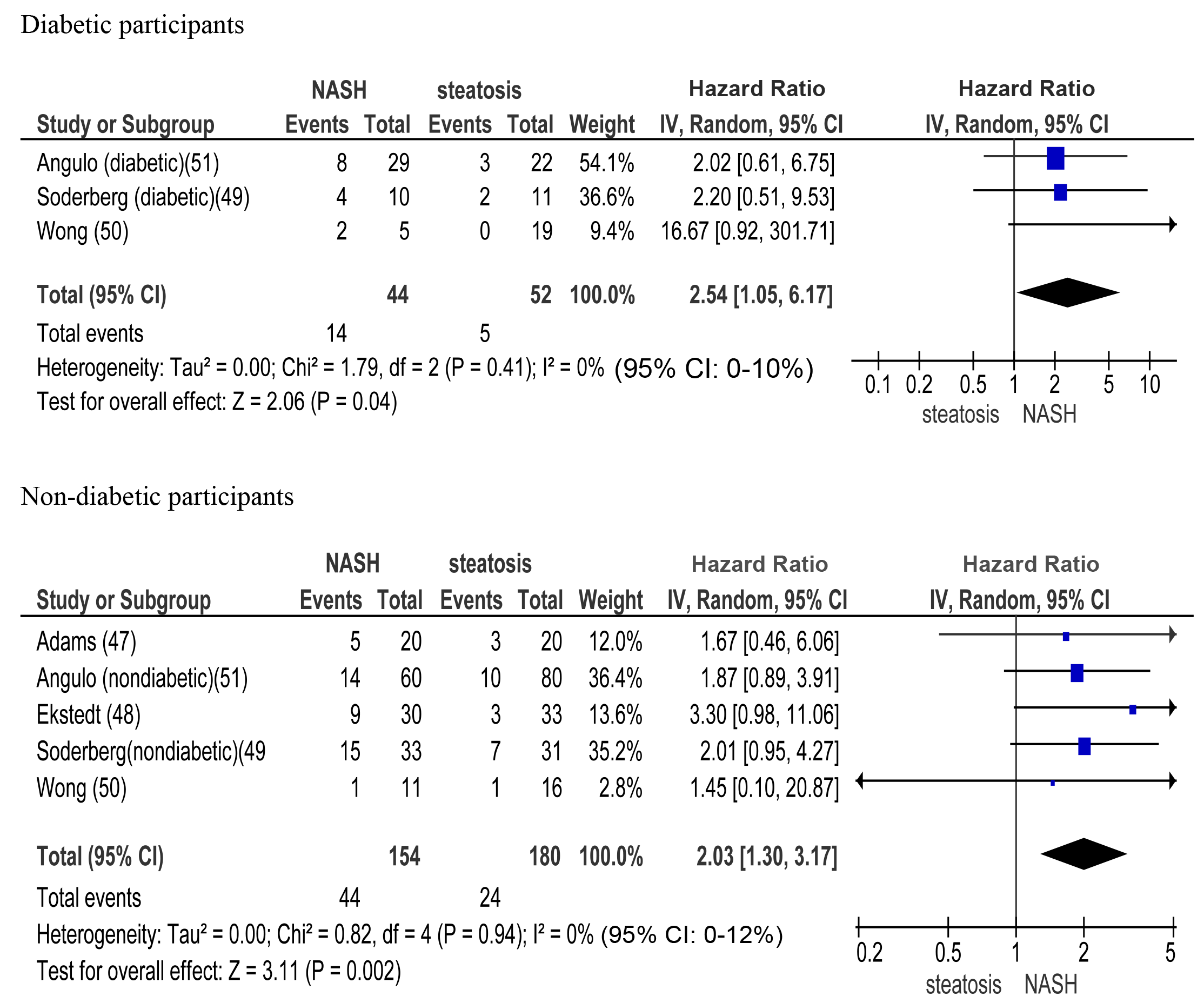


**Figure S45.** Forest plots of subgroup analyses for the outcome: incident chronic kidney disease (CKD) in

NASH vs. simple steatosis in prospective studies. Ethnicity: Asian vs. non-Asian participants.


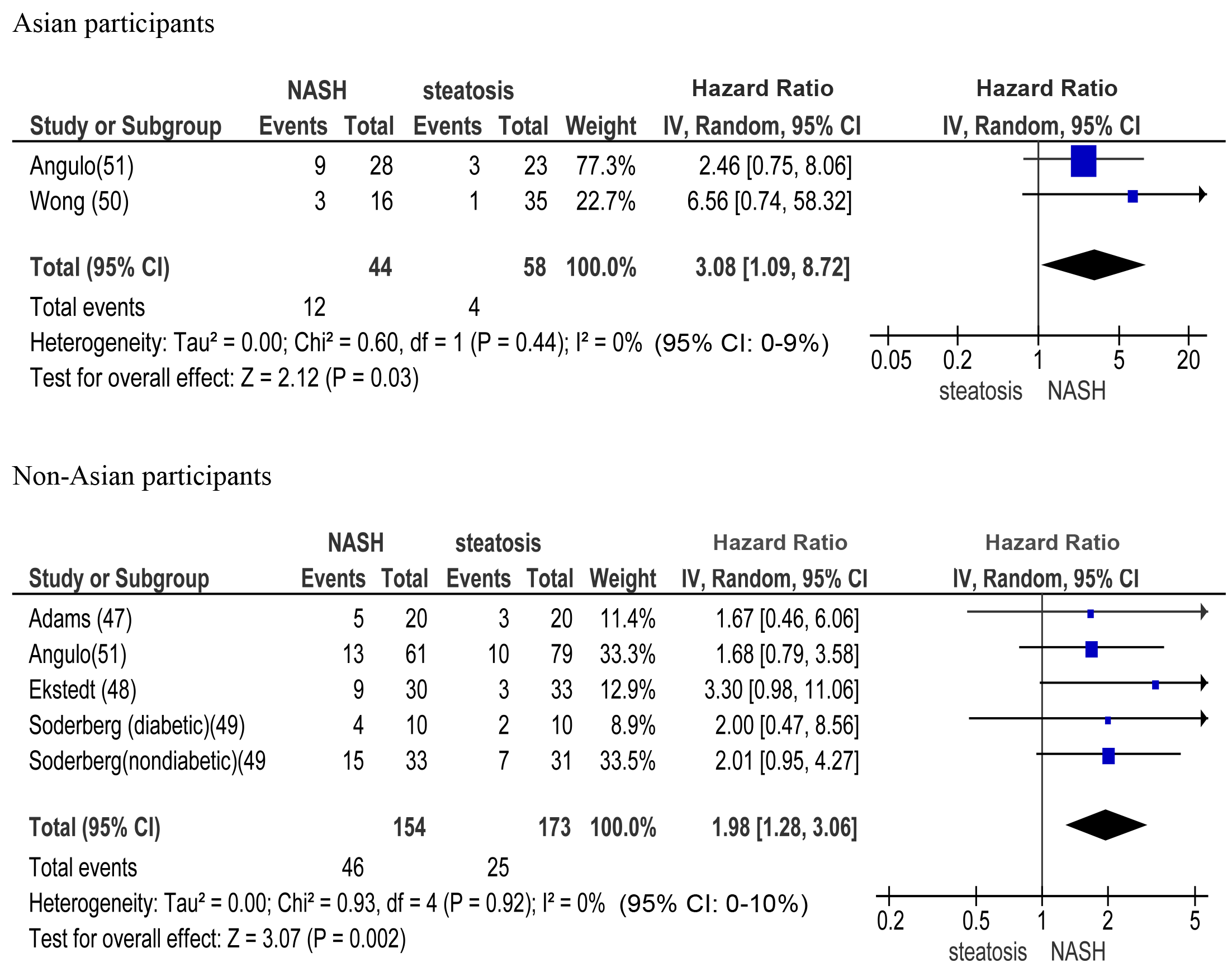


**Figure S46.** Forest plots of subgroup analyses for the outcome: incident chronic kidney disease (CKD) in

NASH vs. simple steatosis in prospective studies. Outcomes related to CKD: both eGFR and proteinuria versus eGFR alone.


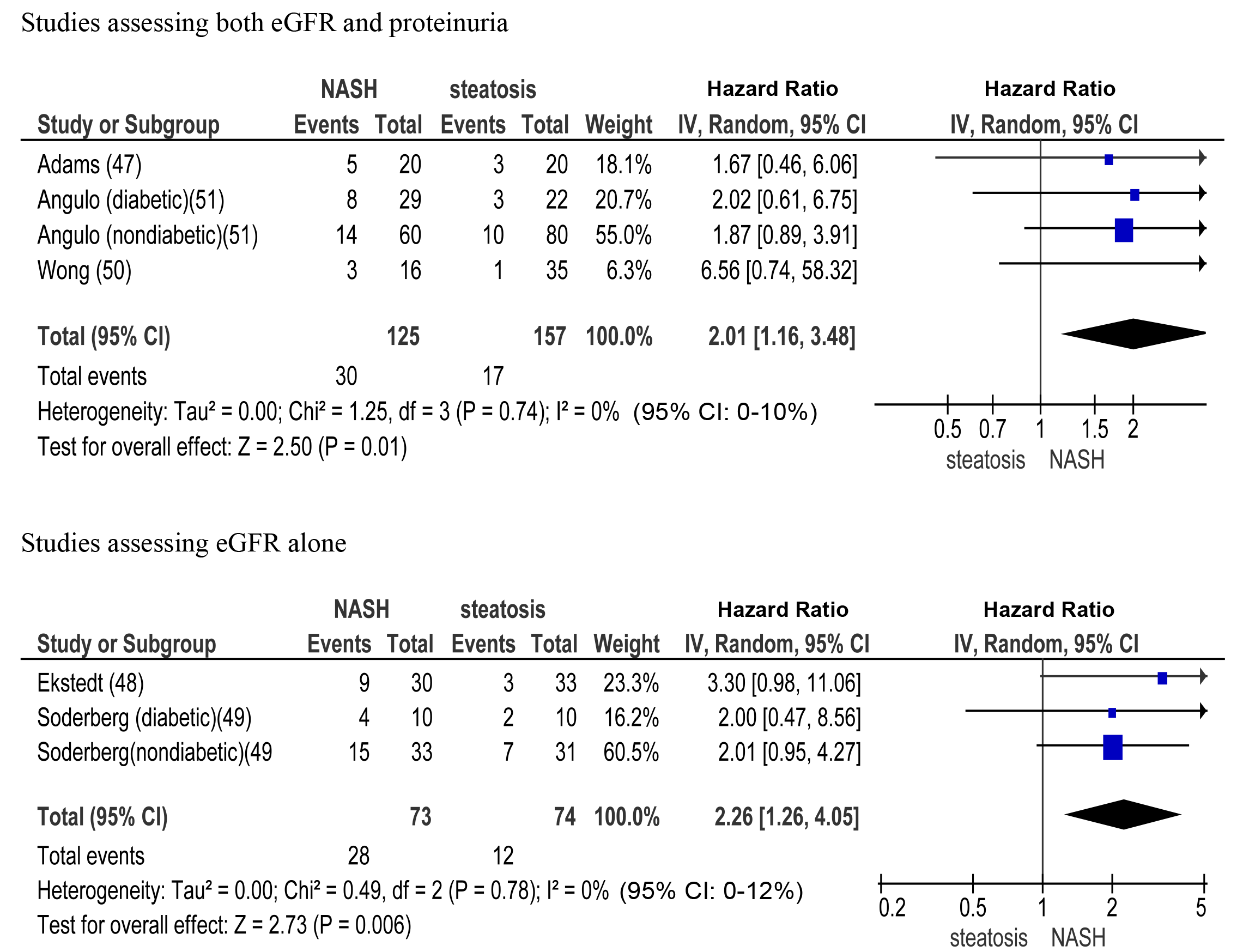


**Figure S47.** Forest plots of subgroup analyses for the outcome: incident chronic kidney disease (CKD) in

advanced (stage F3) vs. non-advanced (stage F0-2) fibrosis in prospective studies. Presence of diabetes.


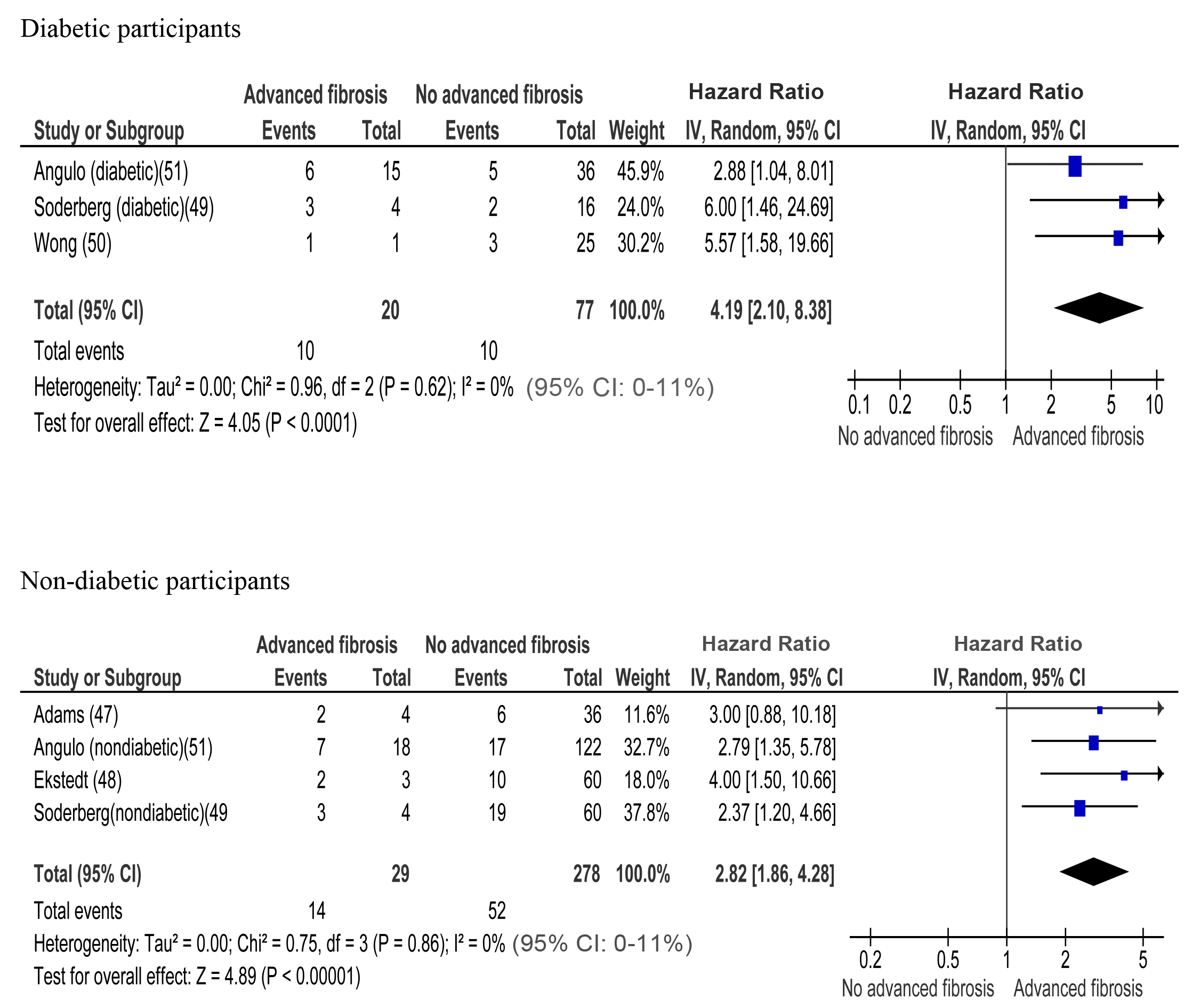


**Figure S48.** Forest plots of subgroup analyses for the outcome: incident chronic kidney disease (CKD) in

advanced (stage F3) vs. non-advanced (stage F0-2) fibrosis in prospective studies. Ethnicity: Asian vs. non-Asian participants.


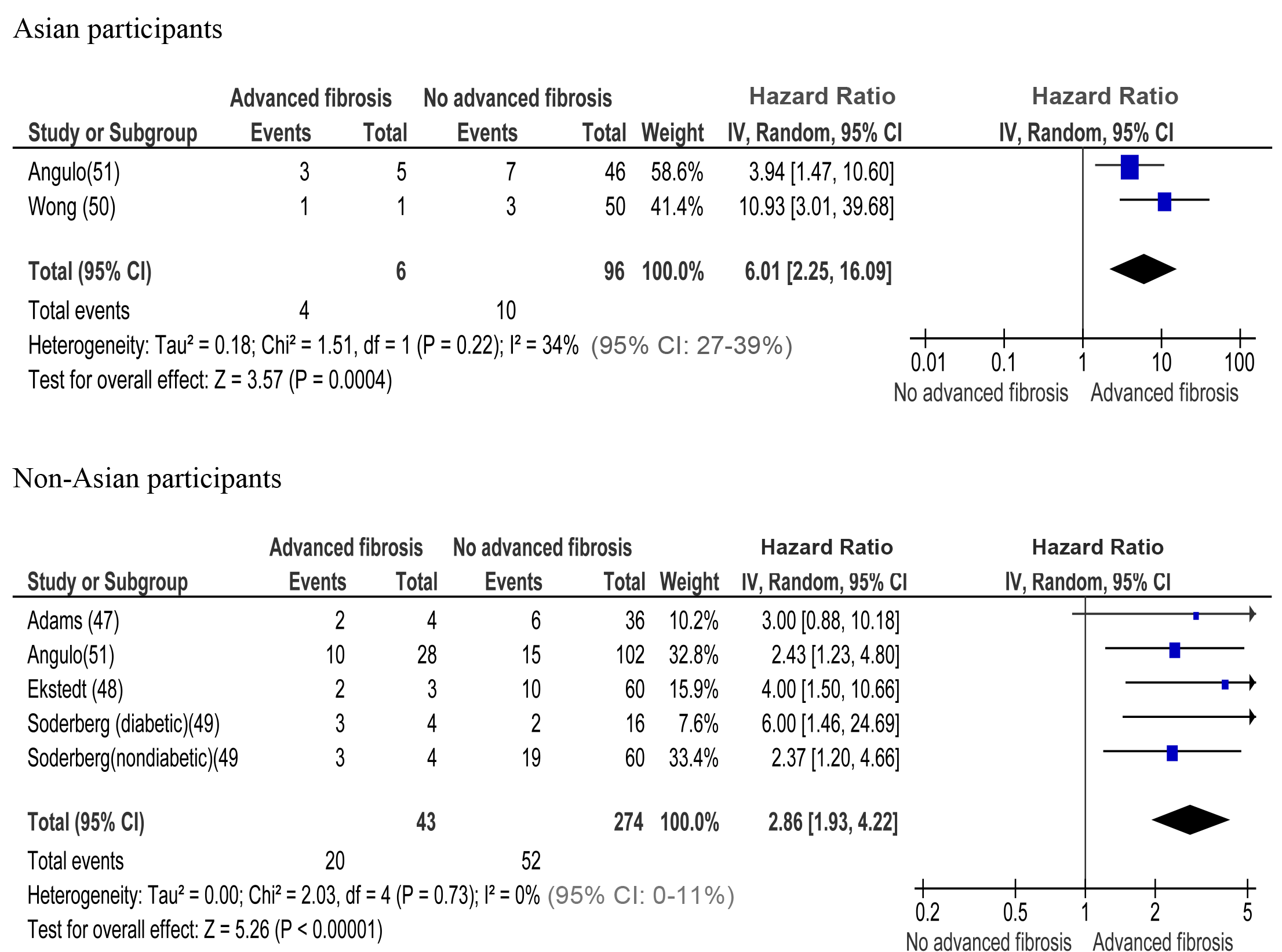


**Figure S49.** Forest plots of subgroup analyses for the outcome: incident chronic kidney disease (CKD) in

advanced (stage F3) vs. non-advanced (stage F0-2) fibrosis in prospective studies. Outcomes related to CKD: both eGFR and proteinuria versus eGFR alone


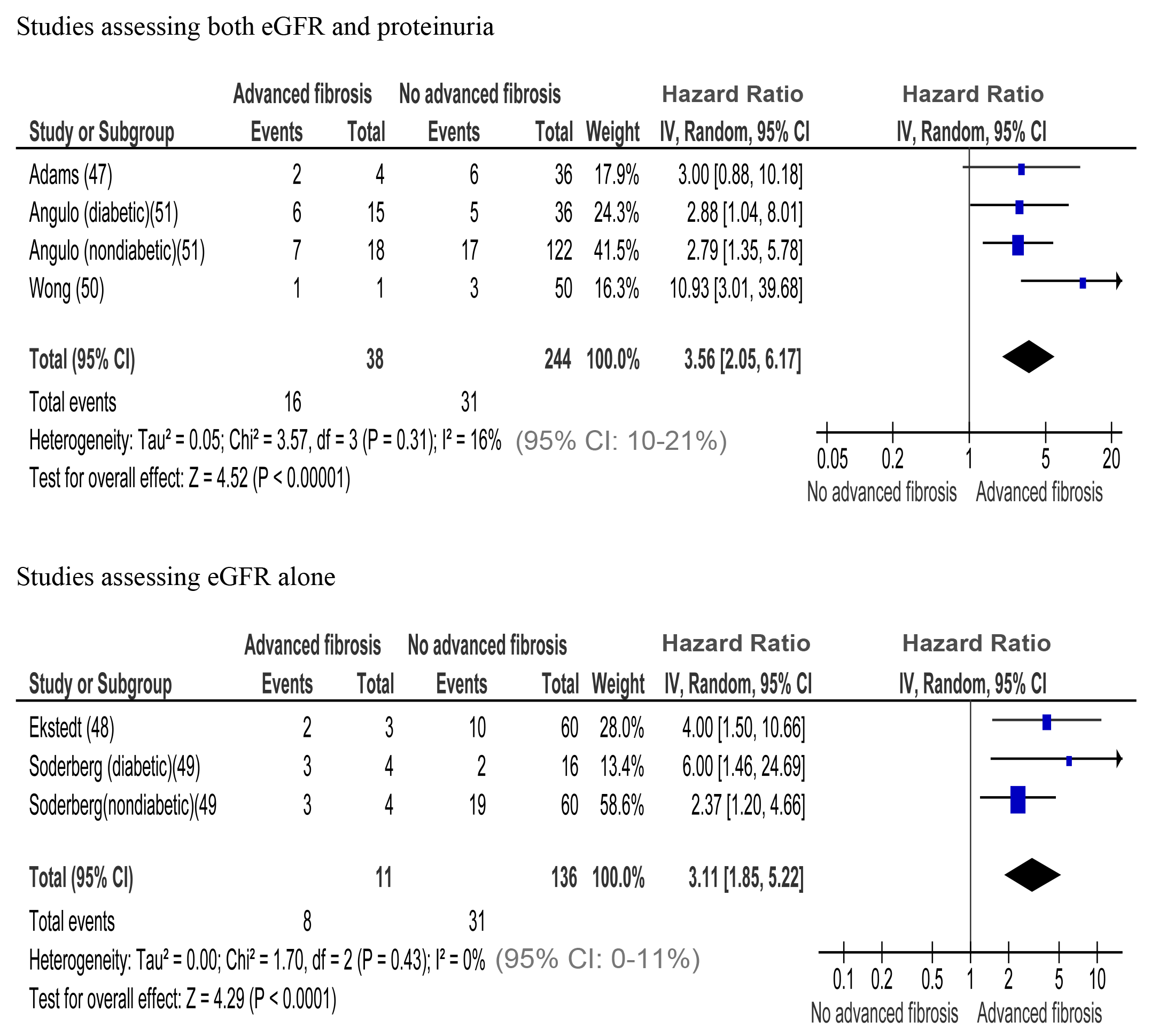

Supplement: Text S3 — Additional analyses. (DOC) [file pmed.1001680.s003.doc]
